# Structure and solution biospeciation on tricarbonylrhenium(I) complexes of mercaptopyrimidines with multifaceted biological activity

**DOI:** 10.1093/mtomcs/mfag002

**Published:** 2026-01-16

**Authors:** Uroš Rapuš, Tamás Pivarcsik, Orsolya Dömötör, Márta Nové, József Nyári, Anita Bogdanov, Gabriella Spengler, Iztok Turel, Jakob Kljun, Éva A Enyedy

**Affiliations:** Faculty of Chemistry and Chemical Technology, University of Ljubljana, Večna pot 113, SI-1000 Ljubljana, Slovenia; Department of Molecular and Analytical Chemistry, University of Szeged, Dóm tér 7-8., H-6720 Szeged, Hungary; Department of Molecular and Analytical Chemistry, University of Szeged, Dóm tér 7-8., H-6720 Szeged, Hungary; Department of Molecular and Analytical Chemistry, University of Szeged, Dóm tér 7-8., H-6720 Szeged, Hungary; Department of Medical Microbiology, TAlbert Szent-Györgyi Health Center and Albert Szent-Györgyi Medical School, University of Szeged, Semmelweis u. 6, H-6725 Szeged, Hungary; Department of Medical Microbiology, TAlbert Szent-Györgyi Health Center and Albert Szent-Györgyi Medical School, University of Szeged, Semmelweis u. 6, H-6725 Szeged, Hungary; Department of Medical Microbiology, TAlbert Szent-Györgyi Health Center and Albert Szent-Györgyi Medical School, University of Szeged, Semmelweis u. 6, H-6725 Szeged, Hungary; Department of Molecular and Analytical Chemistry, University of Szeged, Dóm tér 7-8., H-6720 Szeged, Hungary; Department of Medical Microbiology, TAlbert Szent-Györgyi Health Center and Albert Szent-Györgyi Medical School, University of Szeged, Semmelweis u. 6, H-6725 Szeged, Hungary; Faculty of Chemistry and Chemical Technology, University of Ljubljana, Večna pot 113, SI-1000 Ljubljana, Slovenia; Faculty of Chemistry and Chemical Technology, University of Ljubljana, Večna pot 113, SI-1000 Ljubljana, Slovenia; Department of Molecular and Analytical Chemistry, University of Szeged, Dóm tér 7-8., H-6720 Szeged, Hungary

## Abstract

In this study, we report the synthesis and detailed characterization of four novel bidentate (N, N) ligands incorporating a 2-(methylthio)pyrimidine moiety and their *fac*-tricarbonylrhenium(I) complexes (**ReB1**–**ReB4** and **ReB1Aq**) with the general formula *fac*-[Re(CO)_3_(N,N)X]^n+^, with X = Cl^−^ or H_2_O and *n* = 0 or 1. Designed to integrate biologically relevant functionalities, these complexes exhibited promising multifunctional bioactivity. Cytotoxicity assays demonstrated moderate activity (IC_50_ = 11–78 µM) on various human cancer cell lines, with certain derivatives showing notable selectivity toward the Colo205 line. Most of the chlorido complexes effectively inhibited the replication of *Herpes simplex* virus type 2, while **ReB4** displayed significant antibacterial activity against *Staphylococcus aureus*, including methicillin-resistant strains (MIC = 12.5–25 µM), and demonstrated biofilm inhibition. Aqueous stability of these organometallic complexes was thoroughly investigated, and complexes **ReB2** and **ReB3** containing a pyrimidine and a thiazole ring, respectively, gradually decompose in aqueous media, correlating with a decline in anticancer activity. Ligand-exchange processes were observed, in which the chlorido co-ligands were replaced by water, thus affecting the solubility and lipophilicity. The aqua complex **ReB1Aq** exhibited a low chloride affinity, and the p*K*_a_ of the coordinated water molecule was obtained to be ∼8. Its interaction with human serum albumin was investigated in detail and was found to be dominated by non-covalent interactions, indicating that no coordination bond formation occurs with the protein.

## Graphical Abstract

**Graphical Abstract:**
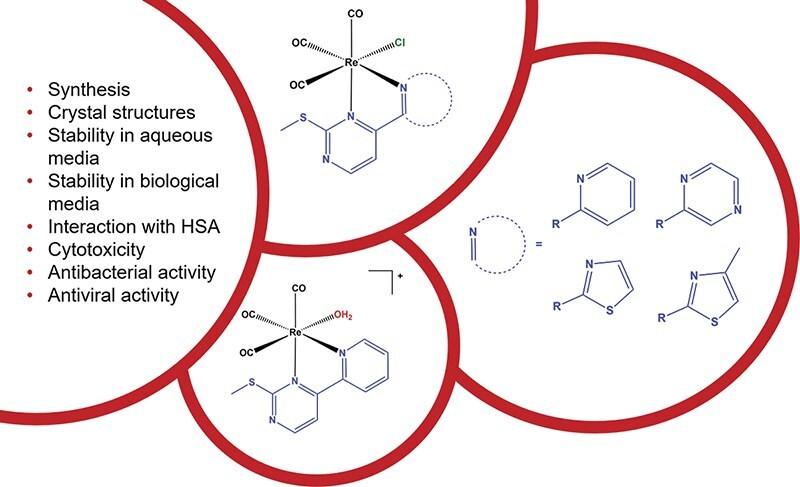

## Introduction

Rhenium(I) tricarbonyl complexes, *fac*-[Re(CO)_3_(N,N)L], represent a promising class of bioactive organometallics with wide therapeutic potential. Here, (N,N) is a bidentate nitrogen ligand, usually 2,2’-bipyridine (bpy) or its analogues, while L represents a (pseudo)halido, nitrogen heterocycle, phosphine, carbene, or thiolato co-ligand. Their unique coordination chemistry imparts exceptional pharmacological activity, positioning them as strong candidates for antimicrobial, antiviral, anticancer, and imaging applications [1–12]. Beyond their biological relevance, carbonyl-rhenium complexes exhibit versatile catalytic and photoactive properties, including applications in CO_2_ reduction, hydrogenation, nitrogen fixation, and in the design of functional metal-organic frameworks [13].

The *fac*-tricarbonylrhenium(I) core represents one of the most versatile building blocks in coordination chemistry, primarily due to its stability and predictable reactivity, as well as commercial availability of shelf-stable precursors [Re(CO)_5_X] (X = Cl^−^, Br^−^). These precursors, available at reasonable cost, typically undergo substitution with bidentate nitrogen ligands, releasing two CO groups to yield *fac*-[Re(CO)_3_(N,N)X]. Such complexes are explored as photoactivated CO-releasing molecules (photoCORMs) with therapeutic potential [14–16]. The halide ligand can be easily displaced using silver salts (e.g. triflate), enabling coordination with solvents (acetonitrile, water) or monodentate ligands (pyridine, 1-methylimidazole, etc.). Notably, derivatives of general formula *fac*-[Re(CO)_3_(N,N)(N)]^+^, containing (N) as a monodentate nitrogen donor, have recently demonstrated significant antimicrobial efficacy. Conjugation of coumarin fragments to either 2,2’-bipyridine or pyridine linkers has resulted in potent antibacterial complexes active against methicillin resistant *Stapylococcus aureus* (MRSA). Moreover, *fac*-[Re(CO)_3_(N,N)(N_azole_)]^+^ compounds, in which N_azole_ represents six different clinically used azole antifungals (e.g. clotrimazole, ketoconazole), have shown markedly enhanced antifungal activity compared to the parent azole ligands, with up to 32-fold decrease in MIC values [17,18].

The rational design of tricarbonylrhenium(I) complexes relies on modulating ligand lability, overall complex charge, and hydrophobic-lipophilic balance. This can be achieved by incorporating electron-withdrawing or electron-donating groups, addition of targeting or stimulus-responsive moieties and modification of the coordination sphere of the metal ion [19]. However, most reported derivatives lack structural diversity, as modifications are predominantly peripheral, leaving the coordination sphere around the rhenium(I) centre largely unchanged. Our research therefore aims to develop innovative ligand frameworks that directly modulate the coordination environment, thereby fine-tuning reactivity and physico-chemical properties of the metal complexes to enhance functionality and expand therapeutic potential.

**Figure 1:**
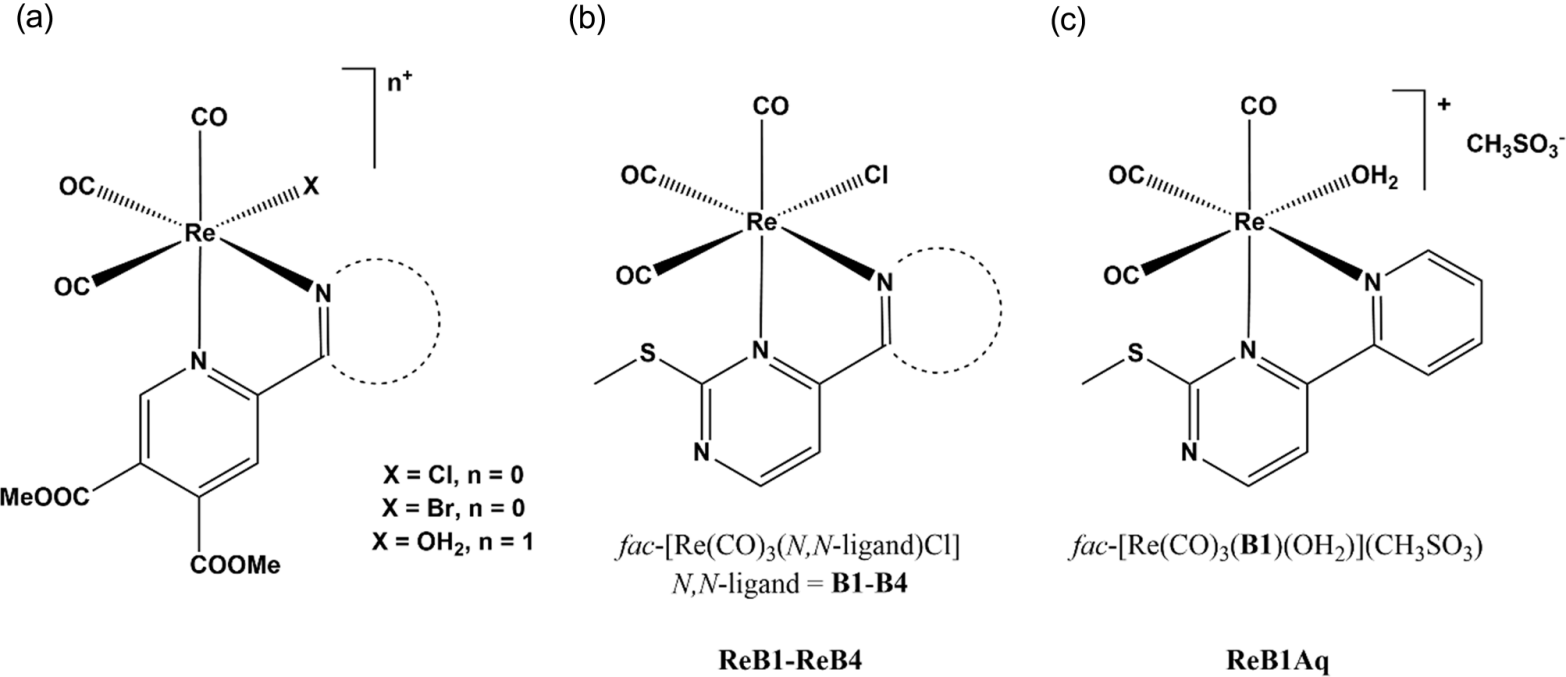
Structures of tricarbonylrhenium(I) complexes with (a) pyridine-4,5-dicarboxylate ester ligands [27], and the work presented in this article, tricarbonylrhenium(I) complexes of chelating methylthiopyrimidines with (b) chlorido or (c) aqua co-ligand.

**Scheme 1:**
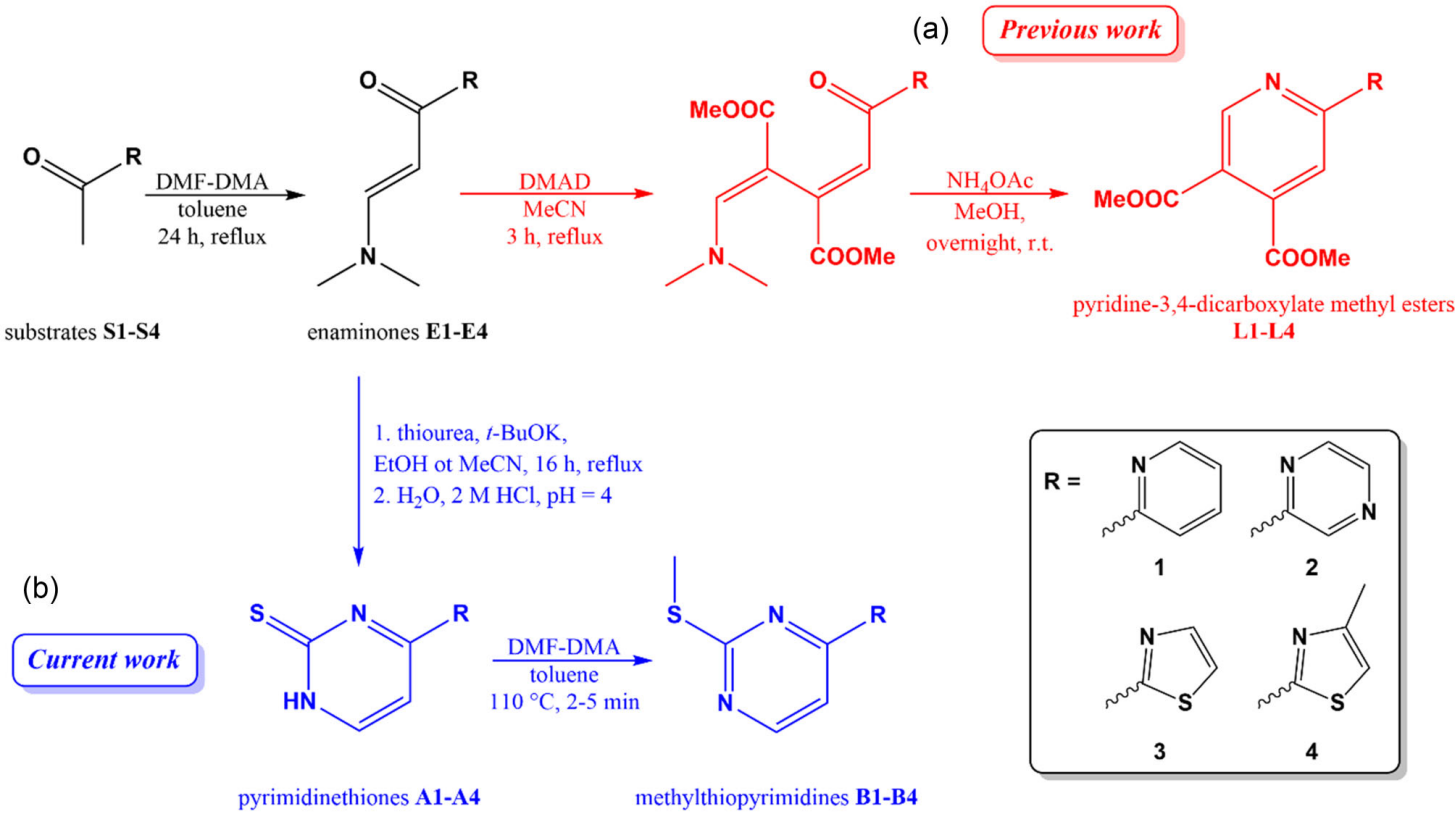
Comparison of (a) previous [27] and (b) current development of novel nitrogen donor chelators based on ring-closing reactions from heteroaromatic enaminone substrates. abbreviations: DMAD, dimethyl acetylenedicarboxylate; mecn, acetonitrile; NH_4_OAc, ammonium acetate; *t*-BuOK, potassium *terc*-butoxide; EtOH, ethanol.

Recognizing the biomedical promise of the tricarbonylrhenium(I)—2,2’bipyridine core, we previously synthesized chlorido-, bromido-, and aqua complexes (Fig. 1a) with four chelating pyridine-4,5-dicarboxylate methyl ester ligands (Scheme 1a) that were developed in our group and used to prepare bioactive metal compounds [20–27]. Speciation studies in aqueous solution revealed remarkable stability across broad pH ranges, with no carbonyl or bidentate ligand release, even in cell culture media and human blood serum. Partial substitution of the halido ligand by water occurred at moderate rates and was reversible in chloride-rich environments, thereby modulating aqueous solubility and lipophilicity together with the process of slow ester hydrolysis occurring on the ligand scaffolds. Deprotonation of the aqua ligand led to the formation of the appropriate hydroxido species, with a p*K*_a_ (H_2_O) ∼8. The chlorido complexes showed moderate cytotoxicity on two human adenocarcinoma cell lines (Colo205 and Colo320) with IC_50_ values in the 60–99 μM concentration range and moderate antiviral activity against *Herpes simplex* virus type 2 [27].

In this work, we report our recent advances in synthesizing a series of chelating mercaptopyrimidines, which were obtained via ring-closing reactions of heteroaromatic enaminone substrates with thiourea, initially affording pyrimidinethione derivatives **A1**–**A4**. Subsequent methylation of the thione sulphur atom then yielded the corresponding methylthiopyrimidine ligands **B1**–**B4**. (Scheme 1b) Pyrimidine-based compounds are well-documented for their broad and diverse range of pharmacological effects [28], and within this class, derivatives bearing sulfur functional groups have demonstrated particularly noteworthy biological activities [28–32].

The ring-closing reaction itself is well-known; however, to the best of our knowledge it has only rarely been applied to nitrogen-containing heteroaromatic substrates, and only in a single case to generate chelating molecules. The synthesis of ligand **A1** with the 2-pyridyl group was previously reported as well as its regioisomers, 3-pyridyl and 4-pyridyl which were prepared in nearly identical reaction conditions [33–36]. Compound **A2** is mentioned in a Chinese patent as an intermediate to the synthesis of the appropriate sodium sulfonate upon oxidation of the thione with peroxide [37]. Methylation reactions with *N, N*-dimethylformamide dimethyl acetal (DMF-DMA) selectively produce *S*-methylated compounds **B1**–**B4**, for which we slightly modified a procedure that Zhu *et al*. used to previously synthesize compound **B1** [38].

The metal-binding properties of the new series of compounds were further evaluated by preparing their respective tricarbonylrhenium(I)-chlorido complexes with the general formula *fac*-[Re(CO)_3_(N,N)Cl] where (N,N) = **B1**–**B4** (Fig. 1b). In the case of **ReB1**, the corresponding aqua complex (**ReB1Aq**) was also prepared (Fig. 1c).

In light of multifaceted biological activities reported for the broader family of rhenium tricarbonyl complexes, the synthesized compounds were subjected to a thorough pharmacological evaluation, including cytotoxicity assays as well as tests for antibacterial, and antiviral properties. In addition to the biological screening, we investigated the solution-phase behaviour of the complexes, with particular attention paid to their stability and speciation in aqueous environments. Moreover, interactions with human serum albumin (HSA) were also systematically examined, offering insights into their potential bioavailability, transport mechanisms, and pharmacokinetic profiles.

## Materials and methods

### Chemicals and general information

All starting materials for the synthesis were purchased from commercial sources (Strem Chemicals, Merck, Fluorochem) and were used as received. Solvents for the reaction were dried over sodium sulfate and molecular sieves (4 Å), while solvents for isolation of the compounds were used without further purification or drying. All other solvents were of analytical grade and used without further purification. KOH, 4-(2-hydroxyethyl)-1-piperazineethanesulfonic acid (HEPES), 2-(*N*-morpholino)ethanesulfonic acid (MES), HSA (A8763, essentially globulin free), D_2_O, Eagle’s Minimum Essential Medium (EMEM), 1-metyhlimidazole (MIM), *N*-acetyl-cysteine (NAC), doxorubicin, cisplatin, and human serum (from human male AB plasma) were Sigma–Aldrich or Merck products and were used without further purification. DMSO, KCl, KNO_3_, HCl, HNO_3_, *n*-octanol were products of Molar Chemicals. DMSO-*d*_6_ was purchased from VWR Chemicals. The reference complex *fac*-[Re(CO)_3_(bpy)Cl] was prepared as reported previously [39].

NMR spectroscopy was performed for the characterization of the compounds on Bruker Avance III 500 or Bruker Avance Neo 600 MHz spectrometer at room temperature. ^1^H NMR spectra were recorded at 500 MHz or 600 MHz. Chemical shifts (δ) are referenced to residual peaks of the deuterated solvent DMSO-*d*_6_ at 2.50 ppm or CDCl_3_ at 7.26 ppm. Chemical shifts and coupling constants (*J*) are given in ppm and Hz, respectively. All NMR data were processed using MestRe-Nova version 14.2.3. Infrared spectra were measured on a Perkin Elmer Spectrum Two with ATR, and IR data was processed using Spectragryph [40]. High-resolution mass spectra (HRMS) were recorded on an Agilent 6224 Accurate Mass TOF LC/MS instrument. Elemental analysis (CHN) for prepared compounds was carried out on a Perkin-Elmer 2400 II instrument. Melting points were determined with OptiMelt MPA 100.

### General procedure for the synthesis of enaminones E1–E4

Enaminones were synthesized according to previously published procedures [21,41]. Briefly, 10–20 mmol of starting 2-acetylheterocyle were mixed with 1.2 molar equivalents of DMF-DMA in ca. 20 mL of dry toluene and the reaction mixture was refluxed for 24 h. The respective enaminone was isolated by reducing the toluene content under reduced pressure and filtering the solid product with vacuum filtration. The products were washed with *n*-hexane and dried for 1 h at 45°C. The enaminones are shelf stable reagents and can be prepared in bulk with yields between 70% and 95%.

### General procedure for the synthesis of pyrimidinethiones A1–A4

Enaminones **E1**–**E4** were mixed with thiourea (1.1 mol. eq.), potassium *terc*-butoxide (*t*-BuOK, 1.1 mol. eq.) and 40 mL of ethanol (EtOH) or acetonitrile (MeCN). The reaction mixtures were refluxed for 16 h. The reaction mixtures were then cooled and 30 mL of solvent was removed under reduced pressure. The concentrated reaction mixture was transferred to a beaker and 30 mL of ice-cold distilled water was added. The pH of the reaction mixture was adjusted to 4 by dropwise addition of 2 M HCl. The precipitates were collected with vacuum filtration and dried overnight at 45°C with yields between 77% and 89%.

4-(pyridine-2-yl)pyrimidine-2(1*H*)-thione (**A1**)

2.286 g (13.0 mmol) of **E1**, 1.088 g (14.3 mmol) thiourea, ethanol, 1.653 g (14.7 mmol) of *t*-BuOK, product **A1**—yellow solid, m = 1.972 g; η = 80.3%. **^1^H NMR** (500 MHz, DMSO-*d*_6_): 13.91 (s, 1H), 8.78 (ddd, J = 4.7, 1.8, 0.9 Hz, 1H), 8.37 (dt, J = 7.9, 1.1 Hz, 1H). 8.14 (t, J = 6.1 Hz, 1H), 8.05 (td, J = 7.7, 1.8 Hz, 1H), 7.70 (d, J = 6.4 Hz, 1H), 7.64 (ddd, J = 7.6, 4.7, 1.2 Hz, 1H) ppm. **^13^C NMR** (126 MHz, DMSO-*d*_6_): 180.98, 165.27, 151.93, 149.86, 147.57, 137.73, 126.82, 122.32, 105.49 ppm. **IR** selected bands (ATR): ῡ = 3136, 3048, 2964, 2897, 2806, 2771, 1601, 1571, 1557, 1476, 1466, 1428, 1403, 1337, 1293, 1257, 1231, 1198, 1175, 1096, 1071, 1093, 994, 975, 912, 886, 836, 785, 749, 712, 645, 618 cm^−1^. **ESI-HRMS** (acetonitrile) for [M + H]^+^ C_9_H_8_N_3_S^+^ found: 190.0431 (calculated: 190.0439). **Elemental analysis** for C_9_H_7_N_3_S calculated (%): C: 57.12 H: 3.73 N: 22.21 found (%): C: 57.05 H; 3.88 N: 21.87. **Melting point:** decomposition at 199°C

4-(pyrazine-2-yl)pyrimidine-2(1*H*)-thione (**A2**)

1.368 g (7.72 mmol) of **E2**, 0.667 g (8.77 mmol) of thiourea, ethanol, 1.003 g (8.94 mmol) of *t*-BuOK, product **A2**—orange solid, m = 1.297 g; η = 88.3%. **^1^H NMR** (500 MHz, DMSO-*d*_6_): 14.04 (s, 1H), 9.46 (d, *J* = 1.3 Hz, 1H), 8.89 (d, *J* = 2.4 Hz, 1H), 8.85 (t, *J* = 2.0 Hz, 1H), 8.19 (d, *J* = 6.2 Hz, 1H), 7.63 (d, *J* = 6.4 Hz, 1H) ppm. **^13^C NMR** (126 MHz, DMSO-*d*_6_): 180.94, 164.13, 147.44, 147.10, 144.59, 143.43, 105.62 ppm. **IR** selected bands (ATR): ῡ = 3142, 3110, 3050, 2963, 2897, 2784, 2711, 2486, 2173, 1967, 1792, 1709, 1601, 1590, 1579, 1562, 1479, 1399, 1344, 1302, 1264, 1240, 1206, 1180, 1149, 1098, 1074, 1035, 1014, 975, 894, 872, 841, 813, 767, 732, 660, 605 cm^−1^. **ESI-HRMS** (acetonitrile) for [M + H]^+^ C_8_H_7_N_4_S^+^ found: 191.0385 (calculated: 191.0391). **Elemental analysis** for C_8_H_6_N_4_S calculated (%): C: 50.51 H: 3.18 N: 29.45 found (%): C: 50.56 H: 2.96 N: 29.31. **Melting point:** decomposition at 191°C

4-(thiazole-2-yl)pyrimidine-2(1*H*)-thione (**A3**)

3.468 g (19.0 mmol) of **E3**, 1.627 g (21.4 mmol) of thiourea, ethanol, 2.352 g (21.0 mmol) of *t*-BuOK, product **A3**—ochre solid, m = 2.858 g; η = 76.9%. **^1^H NMR** (500 MHz, DMSO-*d*_6_): 13.94 (s, 1H), 8.18 (d, *J* = 3.1 Hz, 1H), 8.17 (d, *J* = 3.1 Hz, 1H), 8.13 (t, *J* = 6.0 Hz, 1H), 7.41 (d, *J* = 6.4 Hz, 1H) ppm. **^13^C NMR** (126 MHz, DMSO-*d*_6_): 180.92, 164.95, 159.77, 148.15, 145.79, 127.11, 104.27 ppm. **IR** selected bands (ATR): ῡ = 3126, 3086, 3068, 2937, 2883, 2823, 2774, 2694, 1608, 1571, 1489, 1467, 1414, 1370, 1328, 1294, 1232, 1175, 1160, 1087, 1074, 1021, 979, 961, 888, 883, 796, 772, 670, 636, 608 cm^−1^. **ESI-HRMS** (acetonitrile) for [M + H]^+^ C_7_H_5_N_3_S_2_^+^ found: 196.0001 (calculated: 196.0003). **Elemental analysis** for C_7_H_5_N_3_S_2_ calculated (%): C: 43.06 H: 2.58 N: 21.52 found (%): C: 43.04 H: 2.16 N: 21.11. **Melting point:** decomposition at 201°C

4-(4-methylthiazole-2-yl)pyrimidine-2(1*H*)-thione (**A4**)

1.385 g (7.06 mmol) of **E4**, 0.593 g (7.80 mmol) of thiourea, acetonitrile, 0.916 g (8.17 mmol) of *t*-BuOK, product **A4**—golden-yellow solid, m = 1.311 g; η = 88.8%. **^1^H NMR** (500 MHz, DMSO-*d*_6_): 13.90 (s, 1H), 8.11 (t, *J* = 5.4 Hz, 1H), 7.75 (d, *J* = 1.0 Hz, 1H), 7.37 (d, *J* = 6.4 Hz, 1H), 2.49 (d, *J* = 1.0 Hz, 3H) ppm. **^13^C NMR** (126 MHz, DMSO-*d*_6_): 180.89, 163.90, 159.65, 155.46, 147.99, 121.98, 104.17, 16.93 ppm. **IR** selected bands (ATR): ῡ = 3127, 3101, 3072, 3024, 2952, 2890, 2832, 2778, 2704, 2635, 1779, 1637, 1609, 1553, 1474, 1448, 1386, 1368, 1326, 1293, 1248, 1201, 1148, 1119, 1090, 1062, 1032, 993, 973, 925, 898, 873, 817, 779, 746, 666, 626 cm^−1^. **ESI-HRMS** (acetonitrile) for [M + H]^+^ C_8_H_8_N_3_S_2_^+^ found: 210.0152 (calculated: 210.0160). **Elemental analysis** for C_8_H_7_N_3_S_2_ calculated (%): C: 45.91 H: 3.37 N: 20.08 found (%): C: 45.87 H: 3.07 N: 19.80. **Melting point:** decomposition at 183°C

### General procedure for the synthesis of methylthiopyrimidines B1–B4

Compounds **B1**–**B4** were synthesized in one step according to slightly modified literature procedures [42]. As a first step, **A1**–**A4** were weighed 50 mL two-neck flasks and dissolved in 20 mL of toluene. The reaction mixture was flushed with argon throughout the reaction. DMF-DMA (2 mol. eq.) was added with a syringe and the reaction mixture was placed in a preheated oil bath (120°C) until the mixture became almost clear (up to 5 min, not more) and then immediately cooled in an ice bath. The solvent was then removed under reduced pressure to produce a clear oily residue or solid precipitate. Twenty millilitre of hexane was added to the mixture, which was put in ultrasound bath for one minute to obtain a fine powder precipitate. If the precipitate did not form the solvent was removed under reduced pressure and the procedure was repeated up to twice more. The solid product obtained was dried overnight at 45°C. Reaction yields ranged from 81 to 96%.

2-(methylthio)-4-(pyridine-2-yl)pyrimidine (**B1**)

0.753 g (3.98 mmol) **A1**, 1.0 mL DMF-DMA, product **B1**—white solid, m = 0.778 g; η = 96.2%. **^1^H NMR** (500 MHz, DMSO-*d*_6_, Fig. S1): 8.78 (d, J = 5.1 Hz, 1H), 8.76 (ddd, *J* = 4.7, 1.7, 0.9 Hz, 1H), 8.44 (dt, *J* = 7.9, 1.1 Hz, 1H), 8.07–7.98 (m, 2H), 7.59 (ddd, *J* = 7.5, 4.7, 1.2 Hz, 1H), 2.61 (s, 3H) ppm. **^13^C NMR** (126 MHz, DMSO-*d*_6_): 171.53, 162.23, 158.93, 152.63, 149.75, 137.65, 126.12, 121.34, 112.27, 13.67 ppm. **IR** selected bands (ATR): ῡ = 3102, 3065, 2918, 2739, 2667, 1564, 1531, 1472, 1420, 1350, 1333, 1280, 1247, 1211, 1199, 1083, 1041, 994, 972, 896, 869, 859, 833, 803, 794, 763, 739, 712, 643, 619 cm^−1^. **ESI-HRMS** (acetonitrile) for [M + H]^+^ C_10_H_10_N_3_S^+^ found: 204.0589 (calculated: 204.0595). **Elemental analysis** for C_10_H_9_N_3_S calculated (%): C: 59.09 H: 4.46 N: 20.67 found (%): C: 58.84 H: 4.29 N: 20.86. **Melting point:** 42.5–43.8°C

2-(methylthio)-4-(pyrazine-2-yl)pyrimidine (**B2**)

0.757 g (3.98 mmol) **A2**, 1.0 mL DMF-DMA, product **B2**—yellow solid, m = 0.746 g; η = 91.8%. **^1^H NMR** (500 MHz, DMSO-*d*_6_, Fig. S2): 9.59 (d, *J* = 1.5 Hz, 1H), 8.86 (d, *J* = 2.5 Hz, 2H), 8.86–8.80 (m, 3H), 8.00 (d, *J* = 5.1 Hz, 1H), 2.64 (s, 3H) ppm. **^13^C NMR** (126 MHz, DMSO-*d*_6_): 171.85, 160.74, 159.30, 147.85, 146.99, 144.57, 142.83, 112.65, 13.72 ppm. **IR** selected bands (ATR): ῡ = 3671, 3075, 2988, 2973, 2928, 2902, 1615, 1587, 1558, 1547, 1523, 1474, 1417, 1401, 1356, 1309, 1280, 1261, 1211, 1192, 1159, 1101, 1080, 1016, 981, 963, 872, 834, 775, 744, 720, 658 cm^−1^. **ESI-HRMS** (acetonitrile) for [M + H]^+^ C_9_H_9_N_4_S^+^ found: 205.0543 (calculated: 205.0548). **Elemental analysis** for C_9_H_8_N_4_S calculated (%): C: 52.92 H: 3.95 N: 27.43 found (%): C: 52.50 H: 3.72 N: 27.19. **Melting point:** 122.4–123.8°C

2-(methylthio)-4-(thiazole-2-yl)pyrimidine (**B3**)

0.763 g (3.93 mmol) **A3**, 1.0 mL DMF-DMA, product **B3**—light brown solid, m = 0.735 g; η = 89.3%. **^1^H NMR** (500 MHz, DMSO-*d*_6_, Fig. S3): 8.79 (d, *J* = 5.1 Hz, 1H), 8.13 (d, *J* = 3.1 Hz, 1H), 8.08 (d, *J* = 3.1 Hz, 1H), 7.79 (d, *J* = 5.1 Hz, 1H), 2.59 (s, 3H) ppm. **^13^C NMR** (126 MHz, DMSO-*d*_6_): 171.95, 165.47, 159.26, 157.03, 145.23, 125.08, 110.81, 13.65 ppm. **IR** selected bands (ATR): ῡ = 3681, 3662, 3640, 3125, 3109, 3071, 3048, 3016, 2988, 2920, 2902, 2348, 2173, 1802, 1709, 1662, 1607, 1557, 1541, 1492, 1429, 1392, 1319, 1287, 1218, 1186, 1161, 1085, 1025, 967, 903, 827, 806, 780, 767, 723, 679, 669, 632, 603 cm^−1^. **ESI-HRMS** (acetonitrile) for [M + H]^+^ C_8_H_8_N_3_S_2_^+^ found: 210.0151 (calculated: 210.0160). **Elemental analysis** for C_8_H_7_N_3_S_2_ calculated (%): C: 45.91 H: 3.37 N: 20.08 found (%): C: 45.95 H: 3.20 N: 20.06. **Melting point:** 95.6–96.9°C

2-(methylthio)-4-(4-methylthiazole-2-yl)pyrimidine (**B4**)

1.134 g (5.42 mmol) **A4**, 1.25 mL DMF-DMA, product **B4**—yellow solid, m = 0.985 g; η = 81.4%. **^1^H NMR** (500 MHz, DMSO-*d*_6_, Fig. S4): 8.76 (d, *J* = 5.1 Hz, 1H), 7.73 (d, *J* = 5.1 Hz, 1H), 7.64 (q, *J* = 0.9 Hz, 1H), 2.57 (s, 3H), 2.47 (d, *J* = 1.0 Hz, 5H) ppm. **^13^C NMR** (126 MHz, DMSO-*d*_6_): 171.86, 164.44, 159.13, 156.92, 154.81, 119.71, 110.63, 16.91, 13.61 ppm. **IR** selected bands (ATR): ῡ = 3414, 3164, 3095, 3068, 2959, 2920, 2869, 1660, 1638, 1604, 1557, 1540, 1524, 1467, 1433, 1408, 1369, 1323, 1292, 1256, 1234, 1214, 1187, 1147, 1118, 1086, 1062, 1037, 968, 911, 885, 864, 844, 794, 780, 745, 666, 655, 606 cm^−1^. **ESI-HRMS** (acetonitrile) for [M + H]^+^ C_9_H_10_N_3_S_2_^+^ found: 224.0307 (calculated: 224.0316). **Elemental analysis** for C_9_H_9_N_3_S_2_ calculated (%): C: 48.41 H: 4.06 N: 18.82 found (%): C: 48.16 H: 3.92 N: 18.68. **Melting point:** 72.4–74.1°C

### General procedure for the synthesis of tricarbonylrhenium(I) complexes ReB1–ReB4

Complexes were obtained in one step according to the literature procedure [27]. In a high-pressure tube equimolar amounts of ligand (**B1–B4**), rhenium precursor [Re(CO)_5_Cl] and 10–13 mL of toluene were combined and then heated to 120°C for one hour. The reaction mixtures were cooled and precipitates collected with vacuum filtration. The precipitates were washed with hexane and dried over night at 45°C with yields between 65 and 89%.

*fac*-[Re(CO)_3_(B1)Cl] (**ReB1**)

71.1 mg (0.197 mmol) [Re(CO)_5_Cl]; 40.2 (0.198 mmol) **B1**, product **ReB1**—orange solid, m = 79.6 mg; η = 79.6%. **^1^H NMR** (500 MHz, DMSO-*d*_6_, Fig. S5): 9.16–9.10 (m, 2H), 9.12 (d, *J* = 5.3 Hz, 2H), 8.86 (dt, *J* = 8.3, 1.0 Hz, 1H), 8.45 (d, *J* = 5.3 Hz, 1H), 8.39 (td, *J* = 7.9, 1.5 Hz, 1H), 7.85 (ddd, *J* = 7.7, 5.5, 1.3 Hz, 1H), 2.69 (s, 3H) ppm. **IR** selected bands (ATR): ῡ = 3904, 3788, 3116, 3071, 3045, 2989, 2939, 2902, 2017, 1888, 1606, 1578, 1544, 1480, 1452, 1411, 1341, 1302, 1264, 1218, 1173, 1117, 1094, 1060, 1028, 1000, 970, 907, 859, 829, 798, 766, 728, 698, 658, 648, 631 cm^−1^. **ESI-HRMS** (acetonitrile) for [M-Cl]^+^ C_13_H_9_N_3_O_3_ReS^+^ found: 473.9910 (calculated: 473.9922), for [M-Cl + CH_3_CN]^+^ C_15_H_12_N_4_O_3_ReS^+^ found: 515.0178 (calculated: 515.0188). **Elemental analysis** for C_13_H_9_ClN_3_O_3_ReS_2_ calculated (%): C: 30.68 H: 1.78 N: 8.26; found (%): C: 31.05 H: 1.75 N: 8.08.

*fac*-[Re(CO)_3_(B2)Cl] (**ReB2**)

142,4 mg (0,394 mmol) [Re(CO)_5_Cl]; 80,4 (0,394 mmol) **B2**, product **ReB2**—dark red solid, m = 131,2 mg; η = 65,6%. **^1^H NMR** (500 MHz, DMSO-*d*_6_, Fig. S6): 10.10 (d, *J* = 1.3 Hz, 1H), 9.22–9.16 (m, 2H), 9.03 (d, *J* = 3.0 Hz, 1H), 8.60 (d, *J* = 5.2 Hz, 1H), 2.71 (s, 3H) ppm. **IR** selected bands (ATR): ῡ = 3923, 3680, 3662, 3075, 2988, 2973, 2940, 2902, 2024, 1952, 1926, 1897, 1576, 1537, 1468, 1432, 1412, 1384, 1334, 1317, 1283, 1218, 1190, 1164, 1116, 1081, 1051, 991, 944, 913, 876, 832, 777, 740, 675, 649, 616 cm^−1^. **ESI-HRMS** (acetonitrile) for [M + NH_4_]^+^ C_12_H_12_ClN_5_O_3_ReS^+^ found: 527.9888 (calculated: 527.9907). **Elemental analysis** for C_12_H_8_ClN_4_O_3_ReS_2_ calculated (%): C: 28.26 H: 1.58 N: 10.99; found (%): C: 28.12 H: 1.44 N: 10.58.

*fac*-[Re(CO)_3_(B3)Cl] (**ReB3**)

70,2 mg (0,194 mmol) [Re(CO)_5_Cl]; 40,6 (0,194 mmol) **B3**, product **ReB3**—red solid, m = 75,7 mg; η =75,7%. **^1^H NMR** (500 MHz, DMSO-*d*_6_, Fig. S7): 9.07 (d, *J* = 5.0 Hz, 1H), 8.46–8.36 (m, 2H), 8.25 (d, *J* = 5.1 Hz, 1H), 2.69 (s, 3H) ppm. **IR** selected bands (ATR): ῡ = 3677, 3662, 3098, 3036, 2978, 2928, 2902, 2018, 1890, 1578, 1537, 1500, 1425, 1344, 1325, 1304, 1223, 1182, 1165, 111, 1097, 1067, 1001, 973, 904, 865, 801, 790, 777, 760, 738, 705, 677, 646, 633, 605 cm^−1^. **ESI-HRMS** (acetonitrile) for [M-Cl]^+^ C_11_H_7_N_3_O_3_ReS_2_^+^ found: 479.9475 (calculated: 479.9486), for [M-Cl + CH_3_CN]^+^ C_13_H_10_N_4_O_3_ReS_2_^+^ found: 520.9742 (calculated: 520.9752). **Elemental analysis** for C_11_H_7_ClN_3_O_3_ReS_2_ calculated (%): C: 25.66 H: 1.37 N: 8.16; found (%): C: 25.44 H: 1.23 N: 8.04.

*fac*-[Re(CO)_3_(B4)Cl] (**ReB4**)

137,2 mg (0,380 mmol) [Re(CO)_5_Cl]; 84,6 (0,380 mmol) **B4**, product **ReB4**—red-brown solid, m = 123,3 mg; η = 61,7%. **^1^H NMR** (500 MHz, DMSO-*d*_6_, Fig. S8): 9.03 (d, *J* = 5.1 Hz, 1H), 8.20 (d, *J* = 5.1 Hz, 1H), 8.10 (d, *J* = 1.1 Hz, 1H), 2.70 (d, *J* = 0.9 Hz, 6H), 2.70 (d, *J* = 0.9 Hz, 3H), 2.69 (s, 3H) ppm. **IR** selected bands (ATR): ῡ = 3922, 3678, 3661, 3125, 3101, 2988, 2972, 2920, 2902, 2116, 2021, 1985, 1906, 1579, 1528, 1434, 1408, 1380, 1338, 1319, 1288, 1215, 1181, 1145, 1108, 1066, 1007, 968, 867, 818, 794, 761, 733, 676, 651, 627 cm^−1^. **ESI-HRMS** (acetonitrile) for [M-Cl]^+^ C_12_H_9_N_3_O_3_ReS_2_^+^ found: 493.9627 (calculated: 493.9637), for [M-Cl + CH_3_CN]^+^ C_14_H_12_N_4_O_3_ReS_2_^+^ found: 534.9892 (calculated: 534.9908). **Elemental analysis** for C_12_H_9_ClN_3_O_3_ReS_2_ calculated (%): C: 27.25 H: 1.71 N: 7.94; found (%): C: 27.14 H: 1.36 N: 7.40.

### Synthesis of the complex with aqua co-ligand, ReB1Aq (*fac*-[Re(CO)_3_(B1)(OH_2_)]CH_3_SO_3_∙2H_2_O)

The complex **ReB1Aq** was prepared according to the published procedure for our rhenium(I)-aqua complexes with pyridine dicarboxylate ligands [27]. The chlorido complex **ReB1** was reacted with silver methanesulfonate in acetone. The reaction was carried out in the dark overnight at room temperature. Formed AgCl was removed by vacuum filtration over celite. The solvent was evaporated and residue was dissolved in mixture of methanol and water in ratio of 99:1 and left to slowly evaporate. Product was obtained after two days when all the solvent evaporated.

*fac*-[Re(CO)_3_(B1)(OH_2_)] (**ReB1Aq**)

120 mg (0.236 mmol) **ReB1**; 53,2 mg (0.262 mmol) silver methanesulfonate, product **ReB1Aq**—orange solid, m = 117.2 mg; η = 84.4%. **^1^H NMR** (600 MHz, CDCl_3_, Fig. S9) δ 9.21 (dt, *J* = 5.4, 1.3 Hz, 1H), 8.83 (d, *J* = 5.1 Hz, 1H), 8.23 (dd, *J* = 8.2, 1.2 Hz, 1H), 8.14 (td, *J* = 7.9, 1.6 Hz, 1H), 7.72 (d, *J* = 5.1 Hz, 1H), 7.66 (ddd, *J* = 7.6, 5.4, 1.3 Hz, 1H), 2.71 (s, 3H), 2.68 (s, 3H) ppm. **IR** selected bands (ATR): ῡ = 3089, 2027, 1908, 1582, 1542, 1479, 1450, 1409, 1344, 1272, 1220, 1180, 1148, 1115, 1059, 1010, 960, 847, 828, 791, 759, 649, 631 cm^−1^. **Elemental analysis** for C_14_H_14_N_3_O_7_ReS_2_ calculated (%): C: 28.67 H: 2.41 N: 7.16; found (%): C: 28.33 H: 2.75 N: 6.81.

### Crystallization and structural characterization by single-crystal X-ray diffraction analysis

Single crystals of **ReB1** and **ReB4** were obtained by dissolving *ca*. 5 mg of compounds in 10 mL of acetone/water 5:1 (*v: v*) solvent mixture. The solutions were left to slowly evaporate overnight. Single crystals of **ReB2** were obtained by dissolving ∼5 mg of compounds in 10 mL of acetone. The solution was left to slowly evaporate overnight. Crystals of **ReB1Aq** were obtained by slow evaporation of the mother solution.

Single crystal X-ray diffraction data was collected at 150 K on a SuperNova diffractometer with Atlas detector using CrysAlis software with monochromated Mo Kα (0.71 073 Å) [43]. The initial structural models were solved with direct methods implemented in SHELXT using the *Olex*2 graphical user interface [44]. A full-matrix least-squares refinement on *F*^2^ magnitudes with anisotropic displacement parameters for all non-hydrogen atoms using *Olex*2 or *SHELXL*-2018/3 was performed [45]. All non-hydrogen atoms were refined anisotropically, while hydrogen atoms were placed at calculated positions and treated as riding on their parent atoms. Details on the crystal data, data acquisition and refinement are presented in Tables S1–2. Mercury [46] was used for the preparation of the figures. CCDC deposition numbers 2509118-2509121 contains the supplementary crystallographic data for compounds **ReB1, ReB3, ReB4**, and **ReB1Aq**, respectively.

### Stock solutions and sample preparation

Milli-Q water or DMSO was used for the preparation of stock and sample solutions for the solution phase studies. Stock solutions of the chlorido complexes were obtained by dissolving an exact amount in DMSO. For **ReB3** complex, water could also be used, while stock solutions of **ReB1Aq** were also prepared in water. Their concentrations were calculated based on a weight-in-volume basis. Stock solutions made in DMSO were diluted with aqueous medium up to maximum 10% (*v/v*) DMSO/H_2_O for sample preparation. HSA stock solutions were prepared in HEPES buffer. The residual citrate content of HSA was removed by repeated ultrafiltration of the protein stock solution, and its concentration was calculated from its UV absorption: *λ*_280 nm_ (HSA) = 36 850 M^−1^ cm^−1^ [47]. A 4-fold dilution of human serum was carried out with HEPES buffer for the stability measurements.

### UV-Visible spectrophotometry

An Agilent Cary 3500 spectrophotometer was utilized to obtain UV-visible (UV-vis) spectra in the wavelength range 200–1100 nm. The path length (*ℓ*) was 1 cm in most cases (the actual *ℓ* is always indicated in the legends of the figures). The concentrations of the complexes were between 50 and 80 µM. The computer program HypSpec [48] was used to calculate the equilibrium constants, using similar approaches to determine p*K*_a_ and log*K*’(H_2_O/Cl^−^) as reported in our previous work [27].

### Determination of distribution coefficients and aqueous thermodynamic solubility

The traditional shake-flask method was used to obtain distribution coefficients of the complexes in *n*-octanol/buffered aqueous solution (20 mM HEPES, pH = 7.4) using an Agilent Cary 3500 spectrophotometer for the analysis. The chlorido complexes were dissolved in the presaturated *n*-octanol. The *n*-octanolic solutions were then gently mixed with the buffered aqueous solution for 24 h using 1:50 *n*-octanol-to-H_2_O ratio, followed by phase separation and UV-vis spectrophotometric analysis. Triplicates were always used. The distribution coefficients (*D*_7.4_) were calculated as in our previous work [27]. In another set of experiments, complex **ReB3**, possessing slightly better solubility, was dissolved in water buffered at pH 7.4 to prepare the initial stock solution, which was then mixed with 20 volumes of *n*-octanol. The measurements were conducted without added chloride ions and with the addition of chloride ions at concentrations 4, 24, or 100 mM.

Thermodynamic solubility (*S*_7.4_) of the chlorido complexes was assessed by measuring the saturation levels in water at pH = 7.4 (10 mM HEPES buffer, 0.1 M KCl) at 25.0 ± 0.1°C as described in our previous work [27].

### Solution phase studies using ^1^H NMR spectroscopy

A Bruker Avance III HD Ascend 500 Plus instrument was used for the solution speciation NMR studies. Spectra were recorded with a WATERGATE water suppression pulse scheme in the presence of 10% (*v/v*) D_2_O in most cases. In some cases, 5–10% (*v/v*) DMSO-*d_6_*/H_2_O solvent mixture was also used. DSS internal standard was added to samples to obtain reference peaks.

### Spectrofluorometry

Fluorescence measurements were carried out on a Fluoromax (Horiba Jobin Yvon) spectrofluorometer using a 1 cm × 1 cm quartz cuvette. Samples contained 1 μM HSA or/and 1 μM site marker, and the complex concentration was varied between 0 and 100 or 200 μM. Spectroscopic measurements were carried out on individually prepared samples after 24 h. The excitation wavelength was 295 nm for Trp-214 quenching, 310 nm for WF displacement and 335 nm for DG displacement measurements. The calculated conditional stability constants for HSA—complex species (with 1:1 stoichiometry) were obtained using the computer program HypSpec [48]. Calculations were always based on data obtained from at least two independent measurements. Self-absorbance and the inner filter effect had to be taken into account [49], and corrections were made as described in our previous works [50,51].

### Membrane ultrafiltration

Milli-pore, Amicon Ultra-0.5 membrane filters (10 kDa) were used to separate samples containing 25 μM HSA and 50 μM complex into low and high molecular mass fractions, as described in our previous work [52]. UV-vis spectrophotometry was used to determine the concentration of the non-bound compounds in the low molecular mass fractions by comparing spectra to that of an ultrafiltered reference sample without the protein.

### Equilibrium dialysis

Equilibrium dialysis experiments were conducted in HEPES (pH = 7.4) medium using modified Rapid Equilibrium Dialysis (RED) inserts of Thermo Scientific™. Briefly, the receiver compartment of the RED insert was removed in order to fit the dialysis bag into a regular 1 cm quartz cuvette. The dialysis bag (donor compartment) contained 300 µL aqueous solution containing the complex and the HSA using 1:1 and 1:2 HSA-to-complex concentration ratios or the metal complex alone (*c* = 100 µM), the cuvette was the acceptor/receiver compartment containing HEPES buffer (2.00 mL). Two parallel samples were always prepared. The whole device was capped with aluminium foil and the buffer in the cuvette was continuously stirred; no stirring was applied in the RED insert. On-line monitoring of the receiver compartment was carried out with an Agilent Cary 3500 eight-channel UV-vis spectrophotometer. The concentration of the unbound complex in the cuvette was determined applying external calibration series. The total concentration of the metal complex and HSA in the donor phase was also determined, taking advantage of the fact that the UV-vis spectra of HSA and the metal complex are barely sensitive to the binding (they are practically additive). Donor phase spectra were deconvoluted into the spectra of albumin and the metal complex with MS Excel Solver.

### *In vitro* cytotoxicity assays

#### Cell lines and culture conditions

Human colon adenocarcinoma cell lines Colo205 doxorubicin-sensitive (ATCC-CCL-222), Colo320/MDR-LRP multidrug resistant expressing ABCB1 (MDR1)-LRP (ATCC-CCL-220.1), MCF-7 breast cancer and A549 (CCL-185) lung adenocarcinoma cell lines were purchased from LGC Promochem (Teddington, UK). The CCD-19Lu (CCL-210™) human normal fibroblast cell line was purchased from the American Type Culture Collection (ATCC). The Colo205 and Colo320 cells were cultured in RPMI 1640 medium supplemented with 10% heat-inactivated fetal bovine serum, 2 mM L-glutamine, 1 mM Na-pyruvate and 100 mM HEPES. The MCF-7, A549, and CCD-19Lu cell lines were cultured in EMEM supplemented with a non-essential amino acid (NEAA) mixture (Sigma–Aldrich, a selection of vitamins, 10% heat-inactivated fetal bovine serum (FBS), 2 mM l-glutamine (Sigma–Aldrich), 1 mM Na-pyruvate (Sigma–Aldrich), nystatin (Sigma–Aldrich) and a penicillin—streptomycin mixture (Sigma–Aldrich) in concentrations of 100 U/L and 10 mg/L, respectively. Cell lines were incubated at 37°C, in a 5% CO_2_, 95% air atmosphere. The semi-adherent human colon cancer cells were detached with Trypsin-Versene (EDTA, Sigma) solution for 5 min at 37°C.

#### MTT assay on the used human cancer and CCD-19Lu cells

The tested compounds were dissolved in DMSO to prepare 10 mM stock solutions, which were diluted in complete culture medium, to study the effect of compounds on cell growth of human colon adenocarcinoma cell lines (doxorubicin-sensitive Colo205 and the multidrug resistant Colo320 colon adenocarcinoma cells). Doxorubicin (Merck) was used as a positive control. The cells were treated with Trypsin-Versene (EDTA) solution. They were adjusted to a density of 1 × 10^4^ cells in 100 μL of the appropriate culture medium and were added to each well, with the exception of the medium control wells. Except for the semi-adherent Colo205 and Colo320 cell lines, the other adherent cells were seeded 24 h prior to the assay. Then stock solutions were diluted in the appropriate culture medium, and two-fold serial dilutions of compounds were prepared in 100 μL of the medium, horizontally. The final volume of the wells containing compounds and cells was 200 μL. The plates containing the cancer cells were incubated at 37°C for 72 h; at the end of the incubation period, 20 μL of MTT solution (from a stock solution of 5 mg mL^−1^) were added to each well. After incubation at 37°C for 4 h, 100 μL of SDS solution (10% in 0.01 M HCI) were added to each well, and the plates were further incubated at 37°C overnight. Cell growth was determined by measuring the optical density (OD) at 540/630 nm with a Multiscan EX ELISA reader (Thermo Labsystems, Cheshire, WA, USA). Inhibition of the cell growth (expressed as IC_50_: inhibitory concentration that reduces by 50% the growth of the cells exposed to the tested compounds) was determined from the sigmoid curve where 100 − [(OD_sample_ − OD_medium control_)/(OD_cell control_ − OD_medium control_)] × 100 values were plotted against the logarithm of compound concentrations. Curves were fitted by GraphPad Prism software (2021, Graphpad Software, San Diego, CA, USA) [53] using the sigmoidal dose-response model (comparing variable and fixed slopes). The IC_50_ values were obtained from at least 3 independent experiments.

### Antibacterial activity assay

Wild-type *Escherichia coli* K-12 AG100 strain ATCC 25922 [argE3 thi-1 rpsL xyl mtl Δ(gal-uvrB) supE44] expressing the AcrAB TolC EP at its basal level Gram-negative strain were studied in the experiments in addition to *Klebsiella pneumoniae* ATCC 700603. *Enterococcus faecalis* ATCC 29212, *Staphylococcus aureus* ATCC 25923 and its methicillin-resistant (MRSA) ATCC 43300) variant strains were also used as Gram-positive bacteria in the assays. MIC values of the complexes were determined in 96-well plates based on the Clinical and Laboratory Standard Institute guidelines (CLSI guidelines) [54]. The stock solutions of the compounds (dissolved in DMSO or 20% (*v/v*) DMSO/H_2_O mixture in 5 mM concentration) were diluted in 100 μL of Mueller Hinton Broth. Then 10^−4^ dilution of an overnight bacterial culture in 100 μL of medium was added to each well, with the exception of the medium control wells. The plates were further incubated at 37°C for 18 h; at the end of the incubation period, the MIC values of tested compounds were determined by visual inspection.

### Inhibition of biofilm formation

The anti-biofilm effect of the tested compounds against bacteria strains was evaluated using the crystal violet (CV) assay. This dye [0.1% (*v*/*v*)] is used to detect the total biofilm biomass formed. Overnight cultures were diluted to OD of 0.1 at 600 nm in medium. The bacterial cultures were then added to 96-well microtiter plates, and the compounds were added at sub-inhibitory concentration (1/3 MIC). The final volume was 200 μL in each well. The microtiter plates were incubated at 30°C for 48 h with gentle agitation (100 rpm). After the incubation period, medium was discarded, and the plates were washed with tap water to remove unattached cells. Then 200 μL CV was added to the wells and incubated for 15 min at room temperature. Then, CV was removed from the wells and the plates were washed again with tap water, and 200 μL of 70% (*v*/*v*) ethanol was added to the wells. Finally, the biofilm formation was determined by measuring the OD at 600 nm using Multiscan EX ELISA plate reader (Thermo Labsystems, Cheshire, WA, USA). The anti-biofilm effect of compounds was expressed as a percentage (%) reduction in biofilm formation. The biofilm inhibition (%) was calculated based on the mean of absorbance values.

### Cell cultivation and MTT assay of Vero cells used as host for the evaluation of antiviral activity

Vero cells (ATCC) were placed into the 96-well plate (Sarstedt at a density of 4 × 10^6^ cells/plate. The cells at density of 4 × 10^4^ cells per well were in 100 μL of minimal essential medium (MEM) (Sigma) with Earle salts supplemented with 25 μg mL^−1^ gentamycin, 10% heat-inactivated FBS (Gibco), 8 mM HEPES, 2 mM l-glutamine, 1 × non-essential amino acids, and 1 µg mL^−1^ fungisone. The cells were incubated for 60 min at room temperature to avoid the edge effect and then for 24 h at 37°C, 5% CO_2_ that secure a 90% confluent cell layer [55].

MTT assay was used to determine the maximum non-toxic concentration of the complexes on Vero cells. The cells were grown in 96 well plate at density of 4 × 10^4^ cells per well were in 100 µL of MEM with Earle salts supplemented with 25 µg mL^−1^ gentamycin, 10% heat-inactivated FBS, 8 mM HEPES, 2 mM l-glutamine, 1 × non-essential amino acids, and 1 µg mL^−1^ fungisone. The cells were incubated for 1 h at room temperature and then for overnight at 37°C, 5% CO_2_. When the cell layer reached around 90% confluency the medium was complemented with the serial 2-fold dilutions of complexes. Three parallels were applied for each concentration in the range of 100–0.048 μM for each complex. 10 µL of the MTT labelling reagent was added into wells at 0.5 mg mL^−1^ final concentration. The plate was incubated for 240 min at 37°C, 5% CO_2_ and then 100 µL of the solubilisation solution (10% SDS in 1 M HCl) was added into each well. Next day the absorbance of the wells was determined by a microtiter plate reader (Labsystems Multiskan Ex 355, Thermo Fisher Scientific). The absorbance of the formazan product was measured at 540 nm.

### Antiviral activity assay

#### Cultivation and quantification of Herpes simplex virus type 2

HSV-2 strain was (gift from Dr Ilona Mucsi, University of Szeged, Hungary) grown in Vero (ATCC) cells and the infectivity was determined by using the plaque titration method [56]. The virus titre was expressed as plaque forming units (PFU) [57].

#### Investigation of the impact of complexes on HSV-2 growth in Vero cells

The Vero cells were transferred into the wells of the 96-well plate at a density of 4 × 10^4^ cells/well in 100 μL of Dulbecco’s Modified Eagle’s Medium (DMEM) (Sigma) containing 100 U mL^−1^ penicillin, 100 mg mL^−1^ streptomycin sulphate, 5% FBS, 0.25 g mL^−1^ amphotericin B and 0.14% NaHCO_3_. Prior to infection, the cells were washed with PBS, then were incubated with HSV-2 (multiplicity of infection (MOI): 0.1) for 60 min at 37°C under a 5% CO_2_ atmosphere. Then the cells were washed with PBS again and the culture medium was complemented with the serial 2-fold dilutions of the complexes was added to triplicate wells in the concentration range of 100–0.078 μM. The plates were incubated at 37°C, 5% CO_2_ for 24 h. The plates were evaluated with qPCR at room temperature.

#### Cell lysis and direct quantitative PCR

The supernatants of infected cells were removed and washed with PBS twice and then 100 µL of sucrose-phosphate-glutamic acid buffer (SPG) was added to wells at the end of 24 h infection. The cells were subjected to two freeze-thaw cycles. Templates for qPCR reactions were derived from mixed cell lysates. Process of PCR was described previously [58]. Briefly, we used 5 × HOT FIREPol^®^ EvaGreen^®^ qPCR Supermix (Solis Solis BioDyne) and HSV-2 gD2 gene specific primer in a Bio-Rad CFX96 real time PCR system for qPCR reaction. Sequences of the gD2 gene specific primer pair were the following: gD2-F: 5`-TCAGCGAGGATAACCTGGGA-3`, gD2-R 5`-GGGAGAGCGTACTTGCAGGA-3`. Annealing-extension temperature was 69°C. Cycle where the amplification curve stepped over the baseline can be corresponded to Ct value of a given sample.

## Results and discussion

### Synthesis and characterization

The synthesis of the chelating methylthiopyrimidine ligands **B1**–**B4** consists of three steps. First, the heteroaromatic methyl ketone substrates are converted to their respective enaminones, which are versatile substrates for heterocycle formation. The reactions are scalable to multigram amounts and the products readily precipitate when concentrating and cooling the mother solution. The second step consists of the ring-closing reaction with thiourea under basic conditions. Again, this step is easily performed at gram scale with the main practical issue being the precipitation of large amounts of potassium salts (as *t*-BuOK is used as base) of respective deprotonated pyrimidinethiones **A1**–**A4** during the reaction which might result in difficult stirring of the dense suspensions. The suspensions were thus often diluted slightly during reaction to facilitate effective stirring. Due to a lower reaction yield in the case of compound **A4** ethanol was substituted with isopropanol or acetonitrile. The latter produced the highest yield and purity of the product as determined by CHN elemental analysis. In this second step, the formation of intensely coloured by-products (red, orange or brown) was occasionally observed; however, they were present in such low amounts that they could not be detected by ^1^H NMR analysis and did not affect results of the CHN elemental analysis. Recrystallization from hot ethanol yielded pale orange, pale red or pale brown powders in good yields. Methylation reactions using DMF-DMA proceed very fast. To avoid formation of by-products the reaction time was kept to a maximum of 5 min. The reaction mixture was placed in a pre-heated oil bath and upon completion immediately placed in an ice bath. Occasionally a small amount of black tar was formed and in this case the reaction mixture was carefully decanted or the concentrated reaction mixture filtered through a thin layer of silica and isolation proceeded as described with only minor loss of yield. Scaling above half-gram scale generally results in slightly lower yields and lower purity of products. Multi-gram scale preparation of ligands **B1**–**B4** can easily be achieved by performing parallel reactions at half-gram scale and combining the ice-cold crude reaction mixtures before the work-up and isolation steps. Rhenium complexes of ligands **B1**–**B4** were prepared according to previously published procedures [27]. The reactions consistently produce pure compounds in high yields. The aqua complex **ReB1Aq** was prepared according to the previously published procedure without modifications [27].

### Crystal structure analysis

**Figure 2:**
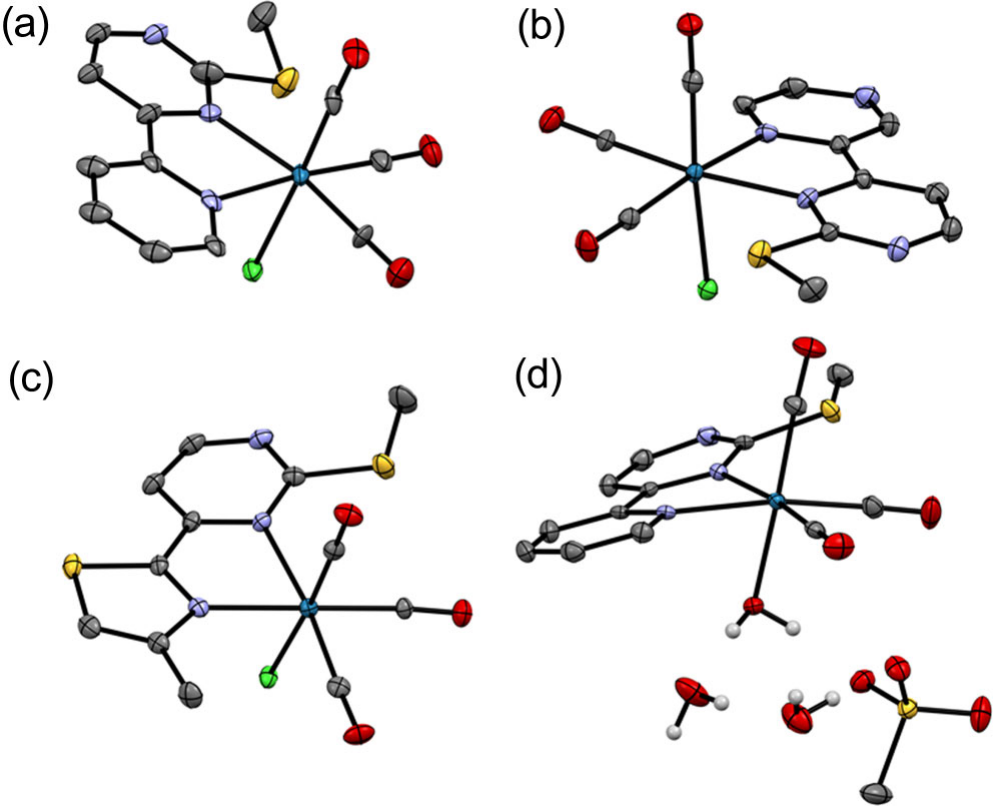
Crystal structures of rhenium complexes (a) **ReB1**, (b) **ReB2**, (c) **ReB4**, and (d) **ReB1Aq**. thermal ellipsoids are drawn at 35% probability level.

The structures of the complexes synthesized show the expected slightly distorted octahedral geometry of the rhenium(I) coordination sphere with the three carbonyl ligands in facial geometry (Fig. 2). All bond length values are within the expected values. While the chlorido complexes are arranged as discrete molecular units the aqua complex **ReB1Aq** crystallizes as a dihydrate in which the aqua ligand, two solvate water molecules and the methanesulfonate anion are interconnected into a two-dimensional hydrogen-bond network. The aqua ligand acts as a double donor, both solvate water molecules as a donor of two and acceptor of one hydrogen bond and the methanesulfonate anion as an acceptor of three hydrogen bonds. Additional crystallographic data, photographs of the analysed crystals and an in-depth analysis of bond lengths in comparison with previously reported analogue complexes with pyridine dicarboxylate ester ligands is given in the SI (Tables S1–S4, Figs S10, S11).

### *In vitro* anticancer activity of the complexes on human cancer cell lines

The cytotoxic activity of the title complexes was studied on Colo205 and Colo320 colon, A549 lung carcinoma and MCF-7 breast adenocarcinoma human cancer cell lines using the colorimetric 3-(4,5-dimethylthiazol-2-yl)-2,5-diphenyl-tetrazolium bromide (MTT) assay. The compounds were also tested against the non-cancerous fibroblast CCD-19Lu cell line to investigate their selectivity for cancer cells. The aqua complex analogue of **ReB1** (**ReB1Aq**) was also evaluated for comparison. Doxorubicin was used as a positive control. IC_50_ values obtained for the complexes in the different cell lines using 72 h incubation time are shown in Table 1. In comparison, the corresponding metal-free ligands (**B1**–**B4**) were also tested on Colo205 and Colo320 cells, but all exhibited IC_50_ values exceeding 100 µM.

**Table 1.** In vitro cytotoxicity of the title complexes expressed in IC_50_ (µM) values, tested in various cancer cell lines (Colo205, Colo320, MCF-7, A549) and in the non-cancerous fibroblast CCD-19Lu cell line using 72 h incubation time. (Positive control: doxorubicin)

IC_50_/µM	Colo205 ^a^	Colo320 ^b^	MCF-7	A549	CCD-19Lu
**ReB1**	10.9 ± 0.1	27.5 ± 2.4	22 ± 2	45 ± 3	37 ± 5
**ReB1Aq**	24.3 ± 0.4	29 ± 1	n.d.	n.d.	n.d.
**ReB2**	29 ± 1	46 ± 1	70 ± 4	>100	85 ± 4
**ReB3**	56 ± 5	78 ± 3	>100	>100	57.0 ± 0.3
**ReB4**	19.3 ± 0.7	24 ± 1	32 ± 3	46 ± 1	27 ± 1
doxorubicin	0.23 ± 0.02	4.7 ± 0.4	0.46 ± 0.01	0.23 ± 0.02	0.20 ± 0.02

a IC_50_ for the reference complex *fac*-[Re(CO)_3_(bpy)Cl] on Colo205: 25.2 µM, and

b on Colo320 cell line: 49.1 µM [27].

**Figure 3:**
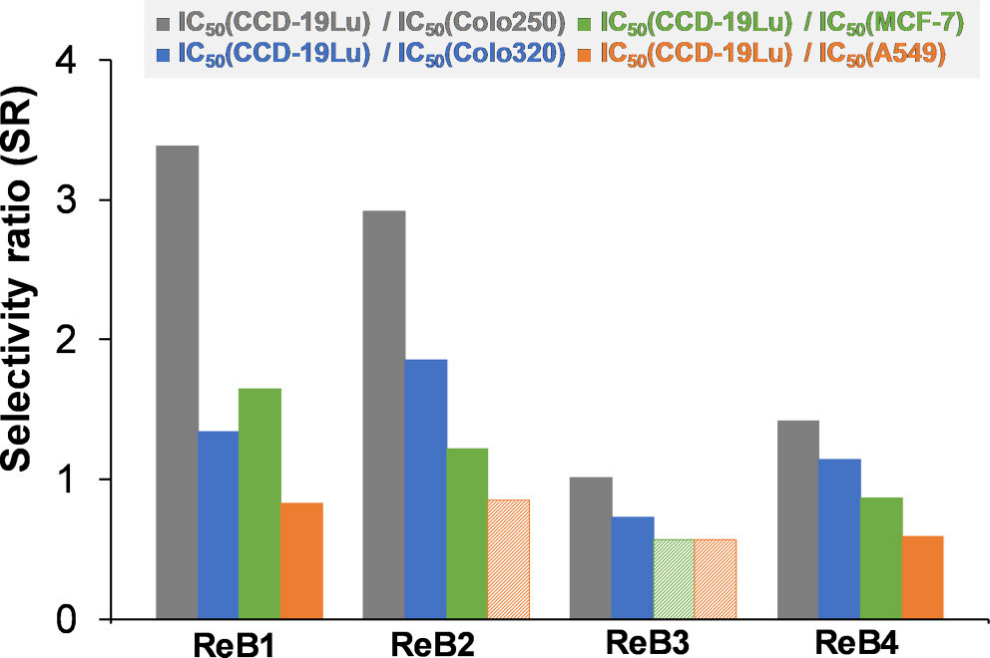
Selectivity ratio (SR) values obtained as quotients of IC_50_ (CCD-19Lu)/IC_50_ (cancer cell line) for the studied organometallic complexes. Striped bars indicate upper limits of the SR values. The IC_50_ values are listed in Table 1.

The complexes displayed moderate anticancer activity with IC_50_ values ranging from 11–78 µM on most of the tested cancer cell lines. However, in some cases (**ReB2** and **ReB3**) IC_50_ values higher than 100 µM were observed. The exchange of the pyridine heterocyclic ring (**ReB1**) to pyrazine (**ReB2**) or thiazole (**ReB3**) decreased the cytotoxicity in all cell lines. Furthermore, the incorporation of a methyl group in the case of **ReB4** considerably decreased the IC_50_ values compared to **ReB3**. Based on the IC_50_ values obtained in the non-cancerous fibroblast cells and the cancer cells, selectivity ratios (SR) were calculated (Fig. 3). Among the tested complexes, **ReB1** and **ReB2** have the highest selectivity (SR > 2.9) against Colo205 cells over the non-cancerous cells. The aqua complex **ReB1Aq** showed similar or slightly weaker cytotoxicity against colon adenocarcinoma cell lines compared to its chlorido counterpart, thus the presence of the chlorido ligand may slightly enhance the overall cytotoxic effect. By comparing these IC_50_ values to those of the reference complex *fac*-[Re(CO)_3_(bpy)Cl] reported previously (Table 1), it can be inferred that the new compounds exhibit comparable activity on Colo205 and Colo320 cell lines, except to **ReB3** [27]. Nevertheless, complexes **ReB1** and **ReB4** demonstrate marginally enhanced cytotoxic activity. In contrast, *fac*-tricarbonylrhenium(I) complexes incorporating mercaptopyrimidine ligands displayed markedly enhanced activity compared to the pyridine-4,5-dicarboxylate ester complexes reported previously [27].

### Antiviral activity of chlorido complexes

**Figure 4:**
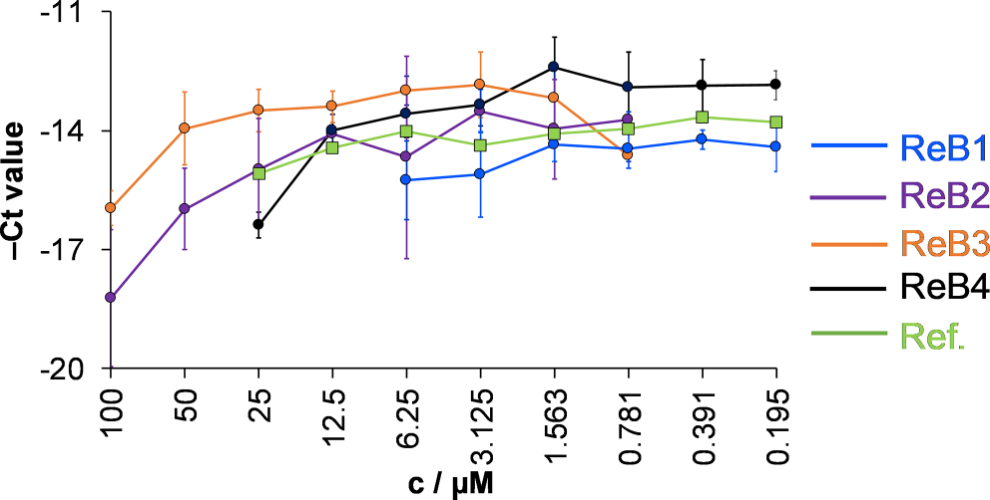
Antiviral activity of the chlorido complexes against HSV-2 at different concentrations. the HSV-2 DNA concentration was measured by direct qPCR. data represent the average − Ct values and their standard deviations. as a comparison, the reference complex (Ref.) *fac*-[Re(CO)_3_(bpy)(Cl)] (in which bpy denotes 2,2′-bipyridine) was also involved, data taken from ref. [27]. {Treatment of the vero cells: 24 h; *n* = 3}

*Herpes simplex* virus type 2 (HSV-2), a double stranded DNA virus that infects mammals, including humans [59], was applied to study the antiviral activity of the chlorido complexes. Vero cells, derived from kidney epithelial cells of the African green monkey, served as host cells for virus propagation. As a first step, the maximum non-toxic concentrations of the complexes were determined on Vero cells using MTT assay (Table S5), which varied in the range of 6.25–100 µM. Then Vero cells were exposed to HSV-2 infection at MOI of 0.1, followed by treatment with the complexes at various concentrations for 24 h. Afterward, the cells were lysed, and the antiviral efficacy of the compounds was assessed by comparing the reduction in viral yield to that observed in untreated Vero cells. Direct quantitative polymerase chain reaction (qPCR) analysis was applied to follow HSV-2 growth. In this assay, the higher cycle threshold (Ct) value indicates that more PCR cycles are required to detect the target nucleic acid, reflecting the presence of lower concentration of vital genetic material, thus stronger antiviral activity. Based on the obtained high Ct values (Fig. 4), all the compounds were able to inhibit the growth of HSV-2 at the concentrations used, and the most effective complex is **ReB2** with a Ct value of 18 ± 2 at 100 µM concentration. Furthermore, the Ct values were compared at 6.25 µM concentration (as this is the highest value where all complexes were tested); and results revealed the highest Ct value (15 ± 1) for **ReB1**. The reference *fac*-[Re(CO)_3_(bpy)Cl] complex and the complexes with the pyridine-4,5-dicarboxylate ester ligands have similar activity [27] compared to the title complexes.

Metal ions and complexes can interact with the polymerase enzyme affecting the qPCR reaction [60]. Therefore, to ensure the reliability of the antiviral assay, direct impact of complexes on DNA polymerase of qPCR was studied (Fig. S12a). Based on the relatively low dCt values (Fig. S12b) obtained for both the treated and untreated samples, there is no significant stimulation or inhibition of the enzyme during the qPCR process.

### Antibacterial effect of the chlorido complexes and their inhibition of biofilm formation

Antibacterial activity of the chlorido complexes was tested in Gram-positive *Staphylococcus aureus* as well as its methicillin-resistant counterpart (MRSA) and in *Enterococcus faecalis* strains. The obtained MIC (minimum inhibitory concentration expressed in µM) values are listed in Table 2.

**Table 2.** Antibacterial activity of the fac-tricarbonylrhenium(I) chlorido complexes expressed as MIC values tested in various Gram-positive bacterial strains.

MIC/μM	*S. aureus* *^a^*	*S. aureus MRSA* *^b^*	*E. faecalis* *^c^*
**ReB1**	50	>100	>100
**ReB2**	100	>100	>100
**ReB3**	50	>100	>100
**ReB4**	12.5	25	50

a ATCC 25923.

b ATCC 43300.

c ATCC 29212.

Complexes **ReB1, ReB2**, and **ReB3** showed weak antimicrobial activity (MIC = 50–100 µM) on the *S. aureus* strain, while no considerable effect (MIC > 100 µM) was observed on the methicillin-resistant *S. aureus* (MRSA) and *E. faecalis* strains. **ReB4** exhibited higher, but moderate activity (MIC = 12.5–50 µM) on the used strains, with a MIC value of 25 µM on MRSA strain known as to be responsible for difficult-to-treat hospital-acquired infections [61]. The compounds were further tested in two Gram-negative bacterial strains, namely, on *Escherichia coli* and *Klebsiella pneumoniae*, however, the complexes showed no activity in these strains, as the MIC exceeded 100 µM in each case. For comparison, the MIC values of the complexes featuring the pyridine-4,5-dicarboxylate ester ligands from our previous study [27] exceed 100 µM against both *S. aureus* and MRSA in all cases.

The **ReB4** complex (MIC < 50 uM) was further tested for ability to prevent the formation and growth of biofilms (communities of microorganisms) of Gram-positive strains. Biofilms contribute to the chronicity of infections, making them harder to eradicate with conventional antibiotics. This complex was found to significantly inhibit the biofilm formation of *S. aureus* MRSA by 44%. Interestingly, no considerable effect was observed for *S. aureus*, while it also showed ∼17% anti-biofilm activity on *E. faecalis* as well.

### Stability and solution chemical properties of the title complexes

As an important part of the characterization of the novel organometallic complexes with multifaceted pharmacological activity, their solution phase chemical properties were studied since these characteristics have a strong impact on the behaviour of the compounds in biofluids, bioavailability and on the mechanism of action. In our previous work, we reported a detailed solution speciation study on *fac*-Re(CO)_3_ complexes formed with various (N,N) bidentate bpy analogues [27]. Based on those results, this type of organometallic complexes could be characterized by high solution stability; the bidentate and the carbonyl ligands remained coordinated in a wide pH range (2–10). However, in the basic pH range (7–10), the proton dissociation of the coordinated aqua co-ligand could be detected, and mixed hydroxido complexes were formed. The halido co-ligand (chlorido or bromido) can undergo slow exchange with water in aqueous solution, and the isolated aqua complexes were found to possess relatively weak chloride ion affinity. To deepen our knowledge of *fac*-Re(CO)_3_ complexes, we aimed to investigate the stability and the possible ligand-exchange processes of the isolated title complexes in solution.

First, UV-vis spectra of the chlorido complexes dissolved in DMSO (Fig. S13) were followed over time. Only the spectra of complexes **ReB1, ReB2**, and **ReB4** were intact over the monitored 60 h, while **ReB3** exhibited a substantial change in its UV-vis spectrum (Fig. S13c), at the applied concentrations (∼60–80 μM).

**Figure 5:**
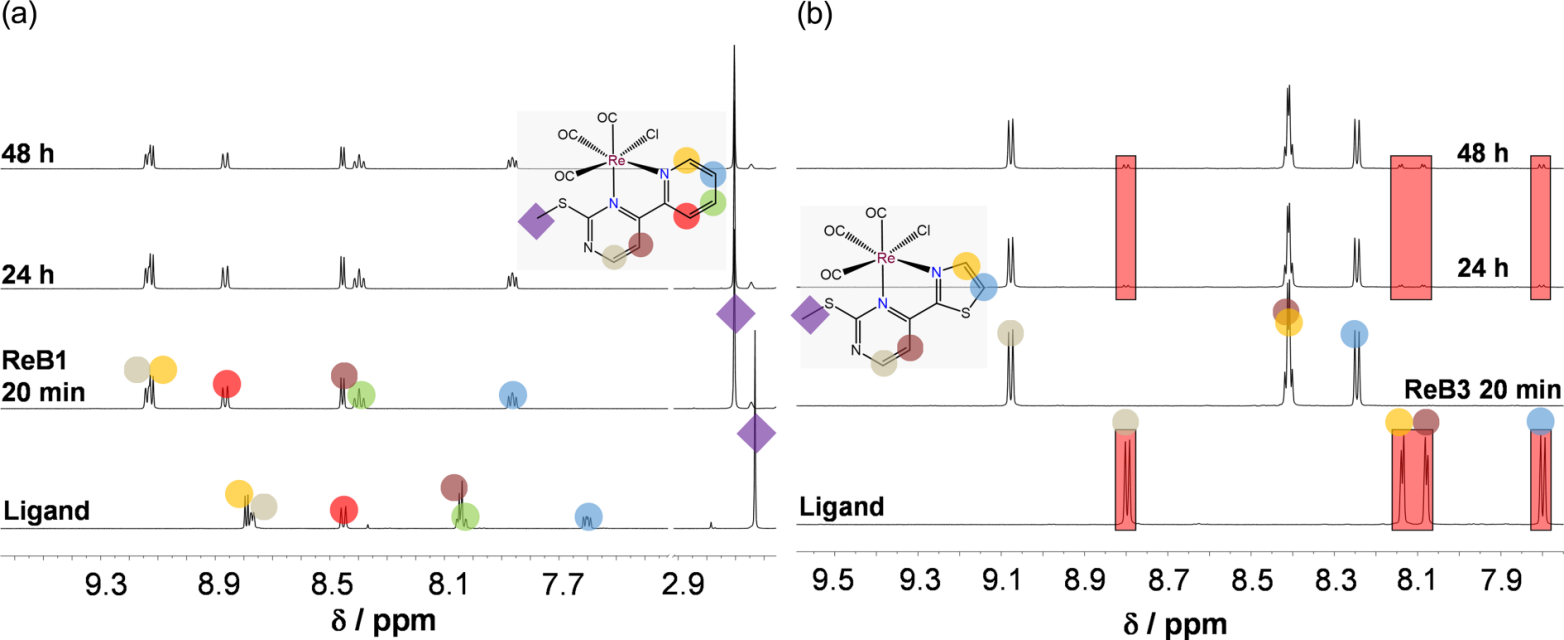
^1^H NMR spectra of a) **ReB1** and b) ReB3 in DMSO-*d*_6_ followed in time and their corresponding (N,N) ligand. Red rectangles indicate the appearance of free ligand. {c_complex_ = 5 mM; T = 25.0°C}

For the better understanding of the changes in the UV-vis spectrum of **ReB3**, ^1^H NMR spectroscopy was also utilized, and ^1^H NMR spectrum of **ReB1** was recorded as well, for comparison (Fig. 5). As expected, ^1^H NMR spectrum of **ReB1** remained unchanged over the 48-h time-period, whereas the appearance of new peaks in the ^1^H NMR spectrum of **ReB3** was observed after 24 h, which evidently belong to the free ligand (Fig. 5b), indicating the complex dissociation and its lower stability. This finding is in line with the lower anticancer activity of **ReB3** compared to the rest of the complexes. Interestingly, the additional methyl group in the case of **ReB4** contributed to its higher stability and higher cytotoxicity and antibacterial activity compared to **ReB4**. The lower stability of **ReB3** cannot be solely attributed to the decreased donor strength of its ligand. According to the predictions obtained with the MarvinSketch software [62] and supported by chemical evidence, the p*K*_a_ values of the uncoordinated heterocyclic nitrogen donors are expected to follow the order: pyrazine (<1) < thiazole (∼1.9) < 4-methylthiazole (2.2) < pyridine (4.0). This trend is consistent with their relative nitrogen donor strength. The higher stability of **ReB4** compared to **ReB3** may arise from the electron-donating effect of the additional methyl substituent. However, **ReB2**, which contains a pyrazine donor, would be expected to be less stable, but this is not the case. This observation suggests that additional factors, such as more favourable π-backbonding or electron delocalization within the coordinated ligand as well as steric effects might contribute to the complex stability.

**Figure 6:**
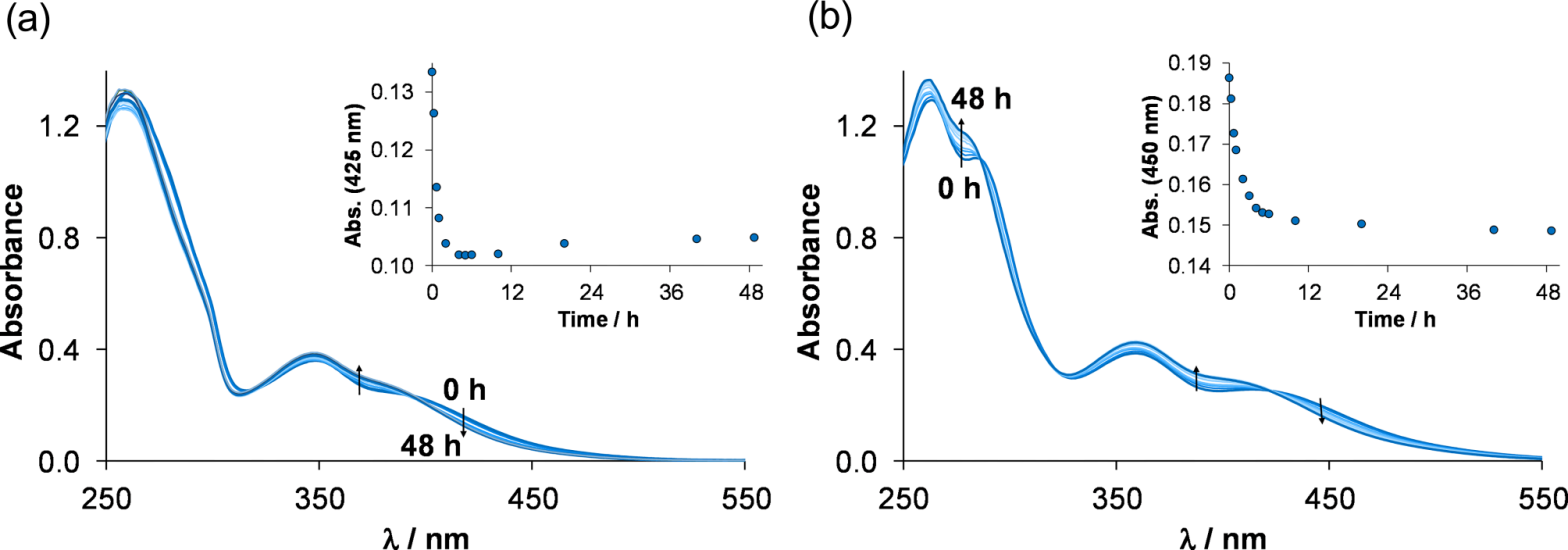
UV-vis spectra of a) **ReB1** and b) **ReB2** in 10% (v/v) DMSO/H_2_O medium followed in time. insets show absorbance values at 425 and 450 nm plotted against time for **ReB1** and **ReB2**, respectively. {c_complex _= 60 and 65 µM; pH ∼ 6.5; ℓ = 1 cm; T = 25.0°C}

As next step, the stability of the chlorido complexes was studied in 10% (*v/v*) DMSO/H_2_O medium at pH ∼6.5 to monitor the impact of solvent H_2_O on the solution speciation. The complexes showed characteristic changes in the UV-vis spectra, which took place within 6 h as it is shown in Fig. 6 and S14; however, these changes indicate only smaller alterations in the coordination sphere. Most probably, the exchange of the coordinated chlorido ligand to H_2_O occurs, as it was previously reported for analogous complexes in our previous work [27]. As a further confirmation, ^1^H NMR spectroscopy was applied for **ReB1** (and **ReB1Aq** was thus prepared for comparison) and showed the appearance of a second peak set after 7 min, indicating the co-presence of aqua and chlorido compounds, and as it was expected, the peaks of the chlorido complex disappeared over time (Fig. S15).

Next, stability of complexes was followed at pH = 7.4 (20 mM HEPES buffer) in the presence of 5% (*v/v*) DMSO. In the case of **ReB1**, the spectral changes are very similar to those obtained without HEPES (in 10% (*v/v*) DMSO/H_2_O). However, for the other complexes (**ReB2, ReB3, ReB4**) the spectral changes are significant, and two processes could be identified over 48 h (Fig. S16). To gain a deeper insight, ^1^H NMR spectroscopy was also applied for **ReB2** and **ReB3**. The exchange of chlorido co-ligand to H_2_O could also be observed in these cases; however, free ligand also appeared after 48 h and 12 h (Fig. S17), respectively, which is line with the UV-vis spectral changes (*vide supra*). The lower stability of **ReB3** was previously observed in DMSO, however, for **ReB2**, dissociation of the complex could only be observed in aqueous environment. Nevertheless, the dissociation of the bidentate ligand under the same conditions is considerably slower for **ReB2** compared to **ReB3**. This can also contribute to the high IC_50_ values of **ReB3**.

The behaviour of complexes was further studied in EMEM (used for the cytotoxicity assays) and in blood serum. The spectral changes in EMEM are also similar to those obtained in HEPES buffer in each case; however, precipitation occurred for **ReB1** (*c* = 60 µM), and especially for **ReB4** (*c* = 73 µM), within the applied 71 h time frame (Fig. S18). It is noteworthy that coordination of EMEM components to the metal centre can be significant, since it contains numerous potential donor molecules, including amino acids and vitamins. In blood serum, the spectral changes also suggest the simultaneous partial dissociation of **ReB2**–**ReB4** and the possible interactions with serum component(s) (Fig. S19) via ligand-exchange.

**Figure 7:**
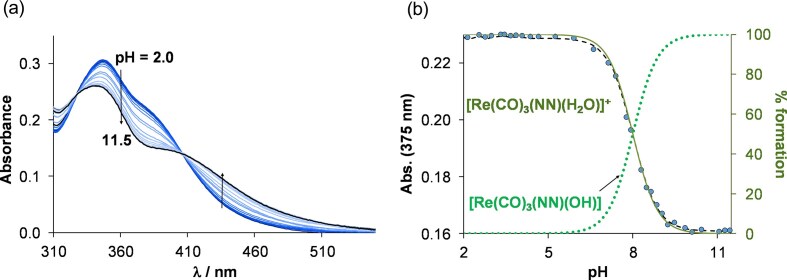
(a) UV-vis spectra of **ReB1Aq** in aqueous solution at increasing pH values (2.0 → 11.5) and (b) absorbance values at 375 nm (●) plotted against pH along with the fitted curve (dashed line) and concentration distribution curves (solid and dotted lines) as a function of pH. {c_complex _= 50 µM; I = 0.1 M KNO_3_; ℓ = 1 cm; T = 25.0°C}

As the chlorido complexes slowly transform into their aqua form, solution chemical properties of the isolated, positively charged aqua complex **ReAq1b** were also investigated, as the aqua form of one of the most promising *fac*-tricarbonylrhenium(I) complex **ReB1**, showing beneficial pharmacological properties (highest cytotoxic activity, selectivity and significant antiviral activity) and aqueous stability. At first, its stability was studied using UV-vis spectroscopy at pH = 7.4 in HEPES buffer and in EMEM over 70 h (Fig. S20). As it was expected, no spectral changes were observed in HEPES, while negligible precipitation occurred after 60 h in EMEM (*c* = 50 µM). Then, UV-vis spectrum of the aqua complex was followed with increasing pH, and the spectra are identical up to pH ∼6.5, then their character was changed between pH 6.5 and 9.5 (Fig. 7).

This process can be attributed to the deprotonation of the coordinated water molecule, based on our previous work where similar *fac-*tricarbonylrhenium(I) complexes were investigated [27]. From the spectral changes, proton dissociation constant (*K*_a_) could be calculated and revealed a similar value (p*K*_a_(H_2_O) = 8.00 ± 0.02 at 0.1 M KNO_3_) compared to those of analogues complexes [27]. Similarly, the pH-dependence of the UV-vis spectra was monitored at 0.1 M KCl ionic strength (using equilibrium time 1 h for the initial sample), and a somewhat higher value (p*K*_a_(H_2_O) = 8.1 ± 0.1) could be obtained due to the weak coordination of the chloride ion to rhenium(I), which slightly suppresses the deprotonation process. Based on the p*K*_a_(H_2_O) values, at pH = 7.4 the estimated fraction of the mixed hydroxido species is ∼20 and ∼15% at ionic strength of 0.1 M KNO_3_ and KCl, respectively.

**Figure 8:**
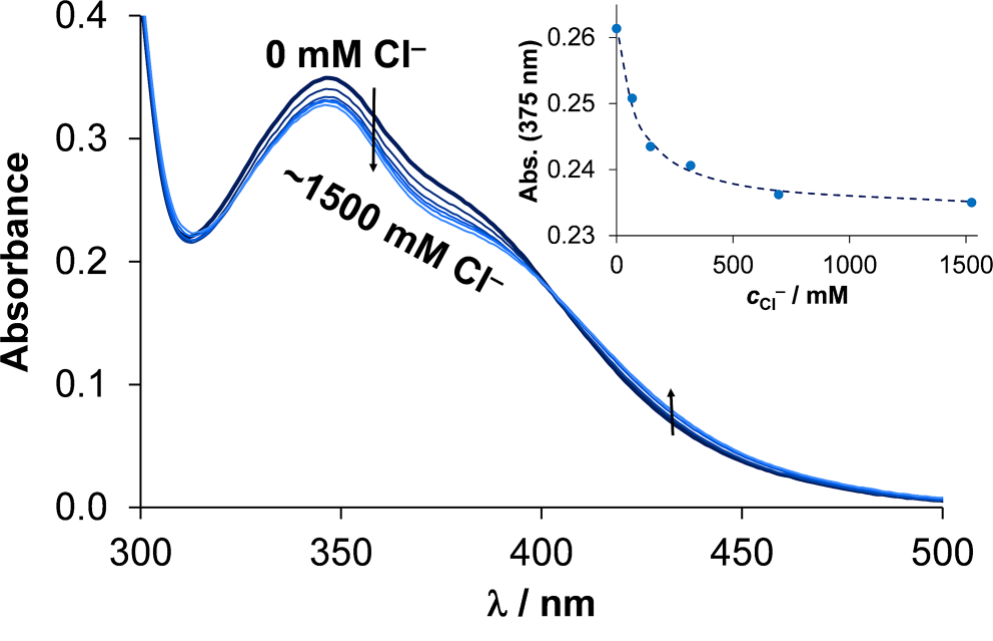
UV-vis spectra of aqua complex **ReB1Aq** (pH = 5.5, 10 mM MES) with increasing chloride ion concentration. inserted figure shows absorbance values at 375 nm (●) plotted against c_Cl^−^_along with the fitted (dashed) line. {c_complex _= 55 µM c_Cl^−^_= 0–1.5 M; ℓ = 1 cm; T = 25.0°C; incubation time: 4.5 h}

The exchange of the aqua co-ligand with chloride ion was investigated by UV-vis spectrophotometry. Based on our previous results [27], analogous complexes have low chloride ion affinity, and the exchange process is not spontaneous (time needed to reach equilibrium is ∼1 h). Therefore, at first, the process was followed in time at a high excess (5000 equivalents) of Cl^−^ at pH 6.0 as indicated in Fig. S21. (At this pH, only the aqua form is present in solution, there are no mixed hydroxido species.) Although the equivalents of Cl^−^ was significantly high, only minor spectral changes could be observed, which suggest low chloride ion affinity and the need of ∼1 h equilibrium time. To determine the chloride ion affinity [expressed as log*K*’ (H_2_O/Cl^−^)] of **ReB1Aq**, UV-vis spectra were recorded for individual samples of the aqua complex at varying chloride ion concentrations, using 4.5 h equilibration period for each batch sample. From the spectral changes shown in Fig. 8, a log*K*’ (H_2_O/Cl^−^) value of 1.03 ± 0.03 value was calculated, which falls within the expected range based on similar investigation of analogous complexes [27].

### Solubility and lipophilicity of the ReB1–ReB4 chlorido complexes

The solubility and lipophilicity of **ReB1**–**ReB4** chlorido complexes were characterized at pH = 7.4 (HEPES buffer). The results (Table 3) revealed low aqueous solubility after 24 h (*S*_7.4_ < 100 µM, at 0.1 M KCl ionic strength) in each case, and the obtained values are even lower compared to the corresponding analogous tricarbonylrhenium(I) complexes containing the pyridine-4,5-dicarboxylate unit instead of the methylthiopyrimidine ring [27].

**Table 3.** Aqueous solubility (S_7.4_, 0.1 M KCl) and lipophilicity (logD_7.4_)^a^ of the complexes **ReB1, ReB2, ReB3**, and **ReB4** at pH = 7.4 (10 mM HEPES buffer). For **ReB3**, the measurements were also done with the addition of chloride ion in 4, 24, and 100 mM concentration. {Equilibration time: 24 h, T = 25.0°C}.

	S_7.4_ (µM)	logD_7.4_
**ReB1**	5.0 ± 0.3	+1.46 ± 0.04
**ReB2**	15 ± 1	+0.99 ± 0.04
**ReB3**	70 ± 7	+1.23 ± 0.08 ^b^
**ReB4**	8 ± 1	+1.72 ± 0.07

a No additional KCl.

b Aqueous stock solution, logD_7.4_ values at different chloride concentrations: +1.22 ± 0.05 (50 μM); +1.25 ± 0.05 (4 mM); +1.38 ± 0.05 (24 mM); +1.43 ± 0.08 (100 mM).

As it is expected, the solubility of **ReB4** is lower than that of **ReB3** due to the additional methyl group in the former complex. It is worth to mention, that the chlorido co-ligand is slowly (not instantaneously) replaced by water after dissolution, resulting in a kinetically hindered aqueous solvation of the complexes, as it was also observed in our previous work for similar complexes [27].

Due to the relatively low aqueous solubility of the complexes, they were dissolved in the presaturated *n*-octanol phase for the partitioning experiment and their log*D*_7.4_ values were determined (Table 3). The complexes possess a lipophilic character; moreover, the additional methyl group present in complex **ReB4** significantly increased the lipophilicity compared to **ReB3**. This increased lipophilicity of **ReB4** most probably contributes to its higher cytotoxicity. The same modification on analogous complexes resulted in a similar trend [27]. The solubility of **ReB3** was sufficient to dissolve the complex in the aqueous phase, therefore, the measurements were repeated starting from the aqueous phase, applying various chloride ion concentrations (4, 24, and 100 mM which are relevant to different biofluids, such as cell nucleus, cytosol and blood plasma, respectively [63]). The experiment was also conducted without the addition of extra chloride ions, and the determined log*D*_7.4_ showed very good agreement with the value obtained from the measurement starting from the *n*-octanol phase (Table 3). It is noteworthy, that these values refer to the corresponding aqua complex as chlorido complexes transform to their aqua form in aqueous environment, as it was also discussed in our previous work [27]. The lipophilicity increases along with *c*(Cl^−^) due to the change in the overall charge (+1 to neutral) when this anion coordinates to the metal centre (Table 3).

### Interaction of the ReB1Aq complex with HSA

HSA is the most abundant serum protein (*ca*. 630 µM) responsible for transporting several endogenous and exogenous (*e.g*. drug molecules) compounds. Consequently, the binding affinity towards HSA is key factor in terms of pharmacokinetic properties of a drug molecule. It has been also shown, that HSA is able to accumulate in tumor tissue due to the enhanced permeability and retention (EPR) effect [64], which can be beneficial in the case of anticancer compounds. Therefore, **ReB1Aq** (due to the favourable pharmacological activity and aqueous stability of its chlorido form **ReB1**) was selected to characterize its HSA binding affinity in detail, using different spectroscopic and separation methods.

Although, tricarbonylrhenium(I) complexes have attracted considerable interest in medicinal chemistry, only a small number of studies have investigated their interactions with serum albumins, including both bovine (BSA) and human variants [65–68]. These studies describe *fac*-Re(CO)_3_ complexes with various coordination environments, including chelating ligands with (N,N), (N,O), and (O,O) donor atom sets. Most of them focus on spectrofluorometric and circular dichroism (CD) spectroscopic experiments aimed at determining the strength of the complex-albumin adduct interactions and assessing conformational changes of the biomolecule. However, the establishment of a coordination bond between the complex and the biomolecule has been less thoroughly explored in these studies, with intermolecular forces being primarily suggested as the driving interaction. In contrast, in our previous work, when the pyrithione ligand with an (O,S) donor atom set was combined with the *fac*-Re(CO)_3_ core, the resulting complex was able to interact with HSA by establishing coordination bond, as well as with 1-methylimidazole (used as a protein binding model) [69]. These findings highlight the significance of the coordination environment around the Re(I) centre, a factor also observed for half-sandwich Ru(II) complexes [50].

**Figure 9:**
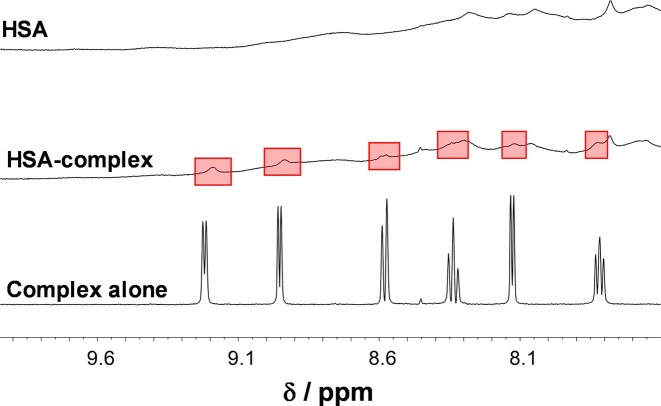
^1^H NMR spectra of **ReB1Aq** complex in the absence and presence of 0.5 equivalent of HSA, and HSA alone in HEPES buffer (pH = 7.4). Red transparent rectangles denote to the HSA-bound complex. {c_HSA_ = 0.25 mM; c_complex_ = 0.5 mM; 10% (v/v) D_2_O; T = 25°C; incubation time: 24 h}

First, overall binding was monitored, and the ^1^H NMR spectrum of the complex in the presence of 0.5 equivalent of HSA was recorded after 24 h (longer incubation time would be pharmacologically irrelevant). The spectrum showed new, more broadened peaks belonging to albumin-bound complex (Fig. 9).

To quantitatively characterize the binding affinity, ultrafiltration-UV-vis method was applied. Regrettably, the compound stuck to the filter to a high degree (∼76%), hindering the quantitative characterization. Therefore, the albumin binding was followed only at 0.5 HSA-to-complex ratio and the UV-vis spectrum of the ultrafiltrated sample was compared to that of ultrafiltrated in the absence of HSA (Fig. S22). Based on the results, approximately, 45% protein-bound fraction is present under these conditions. As ultrafiltration could not be used for trustable quantitative characterization due to the high degree of adhesion on the filter, equilibrium dialysis was applied to further study the global binding of the complex on albumin. In this case, a modified version of the original method was used as a RED insert is placed into a regular 1 cm quartz cuvette without its receiver chamber enabling on-line UV-vis detection of the unbound complex, as detailed in our previous work [70]. In this setup, the cuvette served as the receiver compartment containing HEPES (pH = 7.4) buffer. A special feature of this method is that the originally set analytical concentration ratios (1:1 and 0.5:1 HSA-to-complex) shift in the donor vessel at the end of the distribution, which must be taken into account when evaluating the results. Approximately 3 h were needed for the equilibration in the RED setup, at the end *ca*. 17% of the complex stuck to the cellulose membrane, much less, as it was observed in the ultrafiltration experiments. 56% and 72% of the metal complex bound to the protein at 2.4:1 and 3.5:1 HSA-to-complex final ratios, respectively. Equilibrium dialysis shows moderate binding of **ReB1Aq** to the protein; assuming a 1:1 interaction, a binding constant of log*K*’ = 4.51 ± 0.05 can be calculated based on both ratios used.

In addition, to investigate, whether the interaction is established trough coordination bond or intermolecular forces, UV-vis spectrum of the complex was followed in time in the presence of 0.5 equivalent of HSA as it is shown in Fig. S23. The interaction between the metal complex and albumin is not instantaneous as the rather minor, but detectable spectral changes occur over the investigated 24 h time period. However, this finding does not suggest the simultaneous replacement of aqua co-ligand and coordination of surface-exposed side chain residues (e.g. histidine) of the protein. More probably, intermolecular forces between the complex and HSA are more dominant. To confirm our assumption, further measurements were carried out with 1-metyhlimidazole (MIM) and *N*-acetyl-cysteine (NAC) as histidine (His) and cysteine (Cys) monodentate binding models, respectively. Notably, His residues are considered to be the main coordinating groups in HSA for organometallic Rh(III)(η^5^-pentamethylcyclopentadienyl) and Ru(II)(η^6^-*p*-cymene) complexes [50,51]. As Re(I) cation is a intermediate-to-soft Lewis acid, it prefers sulphur and nitrogen donor atoms as well. Although, HSA contains only one accessible cysteine residue (Cys34), the remaining cysteines are in intramolecular disulfide bridges, and its coordination to **ReB1Aq** is cannot be excluded. The UV-vis spectrum of **ReB1Aq** is intact in the presence of 3 or 10 equiv. MIM over ∼109 min (Fig. S24). As expected, ^1^H NMR spectrum of the complex showed no changes over 24 h (Fig. S25). The UV-vis spectroscopic measurement was also repeated with 10 equiv. NAC and the obtained results were similar (Fig. S26), confirming that coordination of MIM and NAC (and also HSA) to the metal complex is not likely, and albumin binding affinity of the complex is governed by intermolecular forces.

Spectrofluorometric titrations were also conducted to gain more detailed and quantitative insight into the binding affinity within the hydrophobic pockets of HSA. These binding sites, referred to as Sudlow’s sites I and II, are known for hosting a wide range of structurally diverse exogenous and endogenous compounds through intermolecular forces. Tryptophan-214 (Trp-214) quenching measurement (using 24 h incubation time) showed considerable decrease of emission of HSA (∼36%) at ∼110 equivalents of complex (Fig. S27). From the spectral changes a quenching constant of log*K*’_Q_ = 4.7 ± 0.1 could be calculated. However, the partial quenching observed at a high excess of the complex suggests that its binding is not limited to site I. Therefore, as next, site marker displacement experiments were performed with warfarin (WF) and dansylglicine (DG) for site I and II, respectively. The spectrofluorometric experiments revealed also measurable decrease in the emission intensity in both cases after 24 h, when 50–200 equivalents of **ReB1Aq** were used. The necessity of high excess of complex suggests weak-to-moderate affinity toward both binding sites.

To compare the binding strength of **ReB1Aq** complex with its chlorido derivative, spectrofluorometric titration was carried out with **ReB1** as well. In this case, the compound was previously dissolved in DMSO, and the experiment was performed in 4% (*v/v*) DMSO/H_2_O medium as titration. A log*K*’_Q_ = 5.4 ± 0.1 quenching constant was calculated, which is noticeably higher compared to that obtained for the aqua complex. To obtain comparable data, the measurement was repeated as titration in the same medium for the **ReB1Aq** aqua complex as well, however, the difference in the quenching constant is negligible compared to that obtained without DMSO. Most likely, when the complex is in its chlorido form, its higher albumin affinity can be explained by its higher lipophilicity over the aqua complex, allowing stronger binding via hydrophobic interactions.

## Conclusions

In this work, we reported the three-step synthesis and characterization of a series of four 2-(methylthio)pyrimidines with different heteroaromatic substituents on position 4 to produce neutral nitrogen chelating molecules with analogous metal-binding properties to 2,2’-bipyridine as well as their five novel *fac*-tricarbonylrhenium(I) complexes with the general formula *fac*-[Re(CO)_3_(N,N)X]^n+^, in which, the monodentate ligand X is Cl⁻ or H_2_O, and the formed complexes carry an overall charge of *n* = 0 or 1. The synthetic procedures developed allow for a facile gram-scale preparation of this versatile family of bidentate chelators. The synthesis of the respective organorhenium(I) complexes was performed at smaller scale due to the high cost of starting rhenium salts, however the synthetic procedures reliably produce the final metal complexes in both high yield and purity which is promising for potential scaling of production. Single crystal X-ray crystallographic analysis confirmed the anticipated chelating coordination of the bidentate nitrogen-donor ligands, with the coordination sphere of the rhenium(I) ion completed by three carbonyl and a chlorido or aqua ligand.

The synthesized complexes were comprehensively evaluated for their multifaceted pharmacological properties, including cytotoxicity against a panel of cancerous cell lines and a normal human lung fibroblast line (CCD-19Lu) to assess selectivity, as well as antibacterial, and antiviral activities. The findings revealed that these compounds possess notable bioactivity across diverse biological targets. Cytotoxicity assays demonstrated moderate efficacy (IC_50_ = 11–78 µM). Replacing the pyridine ring with either a pyrazine or a thiazole moiety resulted in decreased cytotoxicity due to partial dissociation of the complexes and their reduced lipophilicity. Comparison of the IC_50_ values of **ReB1** and **ReB1Aq** revealed that the presence of the chlorido co-ligand is more favourable than the aqua analogue. Moreover, when compared with the reference complex bearing 2,2’-bipyridine, both **ReB1** and **ReB4**, with increased lipophilicity due to methyl substitution, displayed increased anticancer activity. Notably, **ReB1** and **ReB2** exhibited the highest selectivity ratios (SR > 2.9) for Colo205 colorectal cancer cells over normal fibroblasts. The **ReB4** complex also showed pronounced antibacterial effects against Gram-positive strains, including *Staphylococcus aureus* and its methicillin-resistant variant (*MRSA*), with minimum inhibitory concentrations (MIC) ranging from 12.5 to 50 µM, alongside the inhibition of biofilm formation. Furthermore, complexes also inhibited *Herpes simplex virus 2* replication.

Stability assessments conducted in DMSO for the **ReB1**–**ReB4** complexes, demonstrated the lowest stability of **ReB3**. Importantly, the additional methyl group in **ReB4** significantly enhanced the stability along with increased lipophilicity compared to its, which resulted in higher pharmacological performance. To further evaluate the stability of the complexes, a range of aqueous environments was employed. Notably, **ReB2** and **ReB3** exhibited gradual decomposition at physiological pH (7.4), which was associated with a concomitant reduction in anticancer efficacy. In these aqueous systems, ligand exchange processes were also identified, wherein chlorido ligands were substituted by water molecules, influencing both the solubility and lipophilicity of the complexes. After 24 h of equilibration at pH 7.4, the chlorido complexes displayed limited aqueous solubility (*S*_7.4 _= 5–70 µM) and a lipophilic profile (log*D*_7.4_ = 1–1.7). The aqua complex **ReB1Aq** exhibited low chloride affinity (logK’ (H_2_O/Cl^−^) = 0.60), along with a p*K*_a_ of ~8, belonging to the coordinated aqua ligand.

The interaction between the **ReAq1b** aqua complex and HSA was comprehensively examined using a range of spectroscopic and separation techniques. Global binding studies indicated that the **ReAq1b** complex binds with moderate affinity to the protein. Spectrofluorimetric analyses and protein binding site models experiments employing model ligands such as 1-methylimidazole and *N*-acetyl-cysteine confirmed that the interaction is moderate in strength, characterized by a quenching constant of log*K*’_Q_ = 4.7 ± 0.1, and the binding is primarily mediated by non-covalent interactions, with no evidence of coordination bond formation. Moreover, spectrofluorimetric data indicated that the **ReB1** chlorido complex exhibits a significantly higher quenching constant (log*K*’_Q_ = 5.4 ± 0.1) relative to the **ReB1Aq** aqua analogue, likely attributable to the greater initial lipophilicity of the former one. It is noteworthy, however, that in aqueous environments, the chlorido complexes gradually convert into their corresponding aqua forms over time.

Among the complexes bearing (N,N) donor mercaptopyrimidines, ReB1 with the pyridine moiety proved to be the most promising in terms of both solution phase and biological properties. This complex displays high stability in solution, pronounced lipophilicity, and reversible binding to serum albumin via secondary interactions. We propose that the combination of all these properties contributes to its higher anticancer and antiviral activity.

## Supplementary Material

mfag002_Supplemental_File

## Data Availability

The data underlying this article will be shared on reasonable request to the corresponding author.

## References

[1] Schindler K, Zobi F. Anticancer and antibiotic rhenium tri- and dicarbonyl complexes: current research and future perspectives. Molecules. 2022;27:539. 10.3390/molecules27020539 35056856 PMC8777860

[2] Leonidova A, Gasser G. Underestimated potential of organometallic rhenium complexes as anticancer agents. ACS Chem Biol. 2014;9:2180–93. 10.1021/cb500528c 25137157

[3] Gasser G, Ott I, Metzler-Nolte N. Organometallic anticancer compounds. J Med Chem. 2011;54:3–25. 10.1021/jm100020w 21077686 PMC3018145

[4] Liew HS, Mai C-W, Zulkefeli M et al. Recent emergence of rhenium(I) tricarbonyl complexes as photosensitisers for cancer therapy. Molecules. 2020;25:4176. 10.3390/molecules25184176 32932573 PMC7571230

[5] Konkankit CC, King AP, Knopf KM et al. In vivo anticancer activity of a rhenium(I) tricarbonyl complex. ACS Med Chem Lett. 2019;10:822–7. 10.1021/acsmedchemlett.9b00128 31098006 PMC6512002

[6] Knopf KM, Murphy BL, MacMillan SM et al. In vitro anticancer activity and in vivo biodistribution of rhenium(I) tricarbonyl aqua complexes. J Am Chem Soc. 2017;139:14302–14. 10.1021/jacs.7b08640 28948792 PMC8091166

[7] Collery P, Desmaele D, Vijaykumar V. Design of rhenium compounds in targeted anticancer therapeutics. CPD. 2019;25:3306–22. 10.2174/1381612825666190902161400 31475892

[8] Marker SC, King AP, Granja S et al. Exploring the in vivo and in vitro anticancer activity of rhenium isonitrile complexes. Inorg Chem. 2020;59:10285–303. 10.1021/acs.inorgchem.0c01442 32633531 PMC8114230

[9] Marker SC, MacMillan SN, Zipfel WR et al. Photoactivated in vitro anticancer activity of rhenium(I) tricarbonyl complexes bearing water-soluble phosphines. Inorg Chem. 2018;57:1311–31. 10.1021/acs.inorgchem.7b02747 29323880 PMC8117114

[10] King AP, Marker SC, Swanda RV et al. A rhenium isonitrile complex induces unfolded protein response-mediated apoptosis in cancer cells. Chemistry A European J. 2019;25:9206–10. 10.1002/chem.201902223 PMC6625872 31090971

[11] Karges J, Cohen SM. Metal complexes as antiviral agents for SARS-CoV-2. ChemBioChem. 2021;22:2600–7. 10.1002/cbic.202100186 34002456 PMC8239769

[12] Karges J, Kalaj M, Gembicky M et al. Re^I^ tricarbonyl complexes as coordinate covalent inhibitors for the SARS-CoV-2 main cysteine protease. Angew Chem Int Ed. 2021;60:10716–23. 10.1002/anie.202016768 PMC8014511 33606889

[13] Dilworth JR . Rhenium chemistry—then and now. Coord Chem Rev. 2021;436:213822. 10.1016/J.CCR.2021.213822

[14] Faizan M, Muhammad N, Niazi KUK et al. CO-releasing materials: an emphasis on therapeutic implications, as release and subsequent cytotoxicity are the part of therapy. Materials. 2019;12:1643. 10.3390/MA12101643 31137526 PMC6566563

[15] Sovari SN, Kolly I, Schindler K et al. Efficient direct nitrosylation of α-diimine rhenium tricarbonyl complexes to structurally nearly identical higher charge congeners activable towards photo-CO release. Molecules. 2021;26:5302. 10.3390/MOLECULES26175302 34500734 PMC8434269

[16] Weiss VC, Farias G, Amorim SM et al. Theoretical and experimental mechanistic insights of CO release by water soluble Re(I) PhotoCORMs. Inorg Chim Acta. 2025;587:122788. 10.1016/J.ICA.2025.122788

[17] Sovari SN, Vojnovic S, Bogojevic SS et al. Design, synthesis and in vivo evaluation of 3-arylcoumarin derivatives of rhenium(I) tricarbonyl complexes as potent antibacterial agents against methicillin-resistant Staphylococcus aureus (MRSA). Eur J Med Chem. 2020;205:112533. 10.1016/J.EJMECH.2020.112533 32739550

[18] Schindler K, Demirci G, Tran B et al. Boosting antibiotic efficacy of azole drugs against methicillin-resistant staphylococcus aureus by coordination to rhenium carbonyl complexes. ChemBioChem. 2025;26:e202500368. 10.1002/CBIC.202500368 40557850 PMC12432483

[19] Álvarez D, Menéndez MI, López R. Computational design of rhenium(I) carbonyl complexes for anticancer photodynamic therapy. Inorg. Chem. 2022;61:439–55. 10.1021/acs.inorgchem.1c03130 34913679 PMC8753654

[20] Bezenšek J, Prek B, Grošelj U et al. A simple metal-free synthesis of 2,4,5-trisubstituted pyridines and pyridine N-oxides by [2+2] cycloaddition of enaminones to propyne iminium salts. Z Naturforsch. 2014;69:554–66. 10.5560/znb.2014-4021

[21] Traven K, Sinreih M, Stojan J et al. Ruthenium complexes as inhibitors of the Aldo—Keto Reductases AKR1C1–1C3. Chem Biol Interact. 2015;234:349–59. 10.1016/J.CBI.2014.11.005 25446855

[22] Traven K, Eleftheriadis N, Seršen S et al. Dekker, F. J. Ruthenium complexes as inhibitors of 15-Lipoxygenase-1. Polyhedron. 2015;101:306–13. 10.1016/J.POLY.2015.09.019

[23] Andrejevic TP, Milivojevic D, Glišic B et al. Silver(I) complexes with different pyridine-4,5-dicarboxylate ligands as efficient agents for the control of cow mastitis associated pathogens. Dalton Trans. 2020;49:6084–96. 10.1039/D0DT00518E 32319493

[24] Andrejević TP, Aleksic I, Počkaj M et al. Tailoring copper(II) complexes with pyridine-4,5-dicarboxylate esters for anti-candida activity. Dalton Trans. 2021;50:2627–38. 10.1039/D0DT04061D 33523054

[25] Andrejević TP, Aleksic I, Kljun J et al. Zinc(II) complexes with dimethyl 2,2′-bipyridine-4,5-dicarboxylate: structure, antimicrobial activity and DNA/BSA binding study. Inorganics. 2022;10:71. 10.3390/inorganics10060071

[26] Andrejević TP, Aleksic I, Kljun J et al. Copper(II) and silver(I) complexes with dimethyl 6-(pyrazine-2-yl)pyridine-3,4-dicarboxylate (py-2pz): the influence of the metal ion on the antimicrobial potential of the complex. RSC Adv. 2023;13:4376–93. 10.1039/D2RA07401J 36744286 PMC9890663

[27] Pivarcsik T, Kljun J, Rodriguez SC et al. Structural and solution speciation studies on fac-tricarbonylrhenium(i) complexes of 2,2′-bipyridine analogues. ACS Omega. 2024;9:44601–15. 10.1021/acsomega.4c07117 39524659 PMC11541514

[28] Sharma V, Chitranshi N, Agarwal AK. Significance and biological importance of pyrimidine in the microbial world. Int J Med Chem. 2014;2014:1–31. 10.1155/2014/202784 PMC4207407 25383216

[29] Rashid H, Martines MAU, Duarte AP et al. Research developments in the syntheses, anti-inflammatory activities and structure—activity relationships of pyrimidines. RSC Adv. 2021;11:6060–98. 10.1039/D0RA10657G 35423143 PMC8694831

[30] Sun C, Zhang S, Qian P et al. Synthesis and fungicidal activity of novel 2-(2-alkylthio-6-phenylpyrimidin-4-yl)-1H-benzimidazoles. Bioorg Med Chem Lett. 2021;47:128210. 10.1016/j.bmcl.2021.128210 34157391

[31] Salem MM, Gerges MN, Noser AA. Synthesis, molecular docking, and in-vitro studies of pyrimidine-2-thione derivatives as antineoplastic agents via potential RAS/PI3K/Akt/JNK inhibition in breast carcinoma cells. Sci Rep. 2022;12:22146. 10.1038/s41598-022-26571-7 36550279 PMC9780203

[32] Ray U, Gopinatha VK, Sharma S et al. Identification and characterization of mercaptopyrimidine-based small molecules as inhibitors of nonhomologous DNA end joining. FEBS J. 2023;290:796–820. 10.1111/febs.16615 36048168

[33] Wang F, Schwabacher AW. A convenient set of bidentate pyridine ligands for combinatorial synthesis. Tetrahedron Lett. 1999;40:4779–82. 10.1016/S0040-4039(99)00885-0

[34] Yu MY, Liu JH, Yang J et al. A family of polyoxometalate-resorcin[4]arene-based metal—organic materials: assemblies, structures and lithium ion battery properties. J Alloys Compd. 2021;868:159009. 10.1016/J.JALLCOM.2021.159009

[35] Gannam ZTK, Jamali H, Kweon OS et al. Defining the structure-activity relationship for a novel class of allosteric MKP5 inhibitors. Eur J Med Chem. 2022;243:114712. 10.1016/J.EJMECH.2022.114712 36116232 PMC9830533

[36] Zhu L, Yang X, Luo X et al. A highly selective fluorescent probe based on coumarin and pyrimidine hydrazide for Cu^2+^ ion detection. Inorg Chem Commun. 2020;114:107823. 10.1016/J.INOCHE.2020.107823

[37] Dong H, Ling B, Xie D et al. Method for synthesizing 4-pyrazinyl pyrimidine-2-sodium sulfonate. CN105153123A. 2015.

[38] Zhu H-B, Zhang S-Y, Lu X et al. Syntheses, crystal structures, and luminescence properties of dinuclear metal (Ag^+^ and Cu^+^) Complexes with the Ligand 2-MTPP [2-MTPP = 2-(Methylthio)-4-(Pyridin-2-Yl)Pyrimidine]. Zeitschrift anorg allge chemie. 2011;637:1423–6. 10.1002/ZAAC.201100124

[39] Schneider TW, Ertem MZ, Muckerman JT et al. Mechanism of photocatalytic reduction of CO_2_ by Re(bpy)(CO)_3_Cl from differences in carbon isotope discrimination. ACS Catal. 2016;6:5473–81. 10.1021/acscatal.6b01208

[40] Menges F . Spectragryph-Optical Spectroscopy Software. http://www.effemm2.de/spectragryph/ (accessed on 02.12.2025).

[41] Bezenšek J, Prek B, Grošelj U et al. A simple metal-free synthesis of 2-substituted pyridine-4,5-dicarboxylates and their N-oxides. Tetrahedron. 2012;68:4719–31. 10.1016/J.TET.2012.04.013

[42] Stanovnik B, Tišler M, Hribar A et al. Methylation of Heterocyclic Compounds Containing NH, SH and/or OH Groups by Means of N, N-Dimethylformamide Dimethyl Acetal. Aust J Chem. 1981;34:1729–38. 10.1071/CH9811729

[43] CrysAlis PRO; Oxford Diffraction Ltd: Yarnton, Oxfordshire, UK, 2011;.

[44] Dolomanov OV, Bourhis LJ, Gildea RJ et al. OLEX2: a complete structure solution, refinement and analysis program. J Appl Crystallogr. 2009;42:339–41. 10.1107/S0021889808042726

[45] Sheldrick GM . Shelxl 2018/3, Program for Crystal Structure Refinement; University of Göttingen: Germany, 2018;.

[46] Macrae CF, Edgington PR, McCabe P et al. Mercury: visualization and analysis of crystal structures. J Appl Crystallogr. 2006;39:453–7. 10.1107/S002188980600731X

[47] Beaven GH, Chen S-H, D’albis A et al. A spectroscopic study of the haemin—human-serum-albumin system. Eur J Biochem. 1974;41:539–46. 10.1111/j.1432-1033.1974.tb03295.x 4817561

[48] Gans P, Sabatini A, Vacca A. Investigation of Equilibria in Solution. Determination of Equilibrium Constants with the HYPERQUAD Suite of Programs. Talanta. 1996;43:1739–53. 10.1016/0039-9140(96)01958-3 18966661

[49] Principles of Fluorescence Spectroscopy, 3rd ed.; Lakowicz JR. (ed.); Springer: New York, 2006;. 10.1007/978-0-387-46312-4

[50] Dömötör O, Pivarcsik T, Mészáros JP et al. Critical Factors Affecting the Albumin Binding of Half-Sandwich Ru(II) and Rh(III) Complexes of 8-Hydroxyquinolines and Oligopyridines. Dalton Trans. 2021;50:11918–30. 10.1039/D1DT01700D 34374386

[51] Dömötör O, Enyedy ÉA. Binding Mechanisms of Half-Sandwich Rh(III) and Ru(II) Arene Complexes on Human Serum Albumin: a Comparative Study. J Biol Inorg Chem. 2019;24:703–19. 10.1007/s00775-019-01683-0 31300922 PMC6682546

[52] Dömötör O, Hartinger CG, Bytzek AK et al. Characterization of the Binding Sites of the Anticancer Ruthenium(III) Complexes KP1019 and KP1339 on Human Serum Albumin via Competition Studies. J Biol Inorg Chem. 2013;18:9–17. 10.1007/s00775-012-0944-6 23076343

[53] GraphPad Prism Version 7.00 for Windows, Graph Pad Software, La Jolla, California USA, 2018. **License Serial number:GPS-2615994-LAV1-F3C99 Available online**: https://www.graphpad.com (accessed on 05.12.2025).

[54] CLSI . Susceptibility testing process. In: Christopher PJ, Polgar EP (eds.), Methods for Dilution Antimicrobial Susceptibility Tests for Bacteria that Grow Aerobically, 10th ed.; Clinical and Laboratory Standards Institute: Wayne, MI, USA, 2015;32, pp.15–9.].

[55] Mansoury M, Hamed M, Karmustaji R et al. The Edge Effect: a Global Problem. The Trouble with Culturing Cells in 96-Well Plates. Biochemistry and Biophysics Reports. 2021;26:100987. 10.1016/j.bbrep.2021.100987 33855228 PMC8024881

[56] Abdelmageed AA, Ferran MC. The Propagation, Quantification, and Storage of Vesicular Stomatitis Virus. CP Microbiology. 2020;58:e110. 10.1002/cpmc.110 PMC7449588 32833351

[57] Mucsi I, Molnár J, Motohashi N. Combination of Benzo[a]Phenothiazines with Acyclovir against Herpes Simplex Virus. Int J Antimicrob Agents. 2001;18:67–72. 10.1016/S0924-8579(01)00323-5 11463529

[58] Virók DP, Eszik I, Mosolygó T et al. A Direct Quantitative PCR-Based Measurement of Herpes Simplex Virus Susceptibility to Antiviral Drugs and Neutralizing Antibodies. J Virol Methods. 2017;242:46–52. 10.1016/j.jviromet.2017.01.007 28093274

[59] Mettenleiter TC, Ehlers B, Müller T et al. Zimmermann JJ, Karriker LA, Ramirez A, Schwartz KJ, Stevenson GW, Zhang J, Herpesviruses. In: Diseases of Swine. 11th ed.; Wiley, Hoboken, NJ, 2019.

[60] Zhang X, Guo J, Song B et al. Spatiotemporal Regulation of Metal Ions in the Polymerase Chain Reaction. ACS Omega. 2022;7:33530–6. 10.1021/acsomega.2c04507 36157739 PMC9494670

[61] Bal AM, David MZ, Garau J et al. Future trends in the treatment of methicillin-resistant staphylococcus aureus (mrsa) infection: an in-depth review of newer antibiotics active against an enduring pathogen. J Global Antimicrob Resist. 2017;10:295–303. 10.1016/j.jgar.2017.05.019 28732783

[62] ChemAxon, Ltd. Instant J. Chem./MarwinSketch; ChemAxon Ltd: Budapest, Hungary, 2012.

[63] Martin RB Lippert B, ed. Cisplatin: Chemistry and Biochemistry of a Leading Anticancer Drug. VHCA & Wiley-VCH, Zürich, Switzerland, 1999; pp.181–205. 10.1002/9783906390420

[64] Elsadek B, Kratz F. Impact of albumin on drug delivery ─ new applications on the horizon. J Controlled Release. 2012;157:4–28. 10.1016/j.jconrel.2011.09.069 21959118

[65] Ragone F, Saavedra HHM, García PF et al. Association studies to transporting proteins of fac-Re^I^(CO)_3_(pterin)(H_2_O) complex. J Biol Inorg Chem. 2017;22:99–108. 10.1007/s00775-016-1410-7 27815627

[66] Balakrishnan G, Rajendran T, Murugan KS et al. Synthesis, photophysics and the binding studies of rhenium(I) diimine surfactant complexes with serum albumins: a spectroscopic and docking study approach. J Lumin. 2019;205:51–60. 10.1016/j.jlumin.2018.08.078

[67] Pagoni C-C, Xylouri V-S, Kaiafas GC et al. Organometallic rhenium tricarbonyl—enrofloxacin and—levofloxacin complexes: synthesis, albumin-binding, DNA-interaction and cell viability studies. J Biol Inorg Chem. 2019;24:609–19. 10.1007/s00775-019-01666-1 31111234

[68] Lavor TS, Teixeira MHS, Matos PA et al. The impact of biomolecule interactions on the cytotoxic effects of rhenium(I) tricarbonyl complexes. J Inorg Biochem. 2024;257:112600. 10.1016/j.jinorgbio.2024.112600 38759261

[69] Rapuš U, Pivarcsik T, Mitrović A et al. Synthesis of a fac-tricarbonylrhenium(i) complex with pyrithione, its physicochemical characterization, and assessment of biological effects. ACS Omega. 2025;10:38272–91. 10.1021/acsomega.5c06647 40893321 PMC12391994

[70] Várkonyi EF, Tóth S, Pivarcsik T et al. Organometallic half-sandwich complexes of 1,10-phenanthroline derivatives with improved solubility, albumin-binding, and nanoformulation potential targeting drug resistance in cancer. Inorg. Chem. 2025;64:14914–32. 10.1021/acs.inorgchem.5c01556 40644622 PMC12308784

