# Supplementary material for: Structure and solution biospeciation on tricarbonylrhenium(I) complexes of mercaptopyrimidines with multifaceted biological activity
Source: Metallomics. 2026 Jan 16;18(1):mfag002. doi: 10.1093/mtomcs/mfag002 (PMC13055886; doi:10.1093/mtomcs/mfag002)
Supplement: mfag002_Supplemental_File [file mfag002_supplemental_file.pdf]

SUPPORTING MATERIAL

Structure and solution biospeciation on tricarbonylrhenium(I) complexes of mercaptopyrimidines with multifaceted biological activity

Uroš Rapuš[1], Tamás Pivarcsik[2], Orsolya Dömötör[2], Márta Nové[2,3], József Nyári[3], Anita Bogdanov[3], Gabriella Spengler[2,3], Iztok Turel[1], Jakob Kljun[1,*], Éva A. Enyedy[2,*]

[1] Faculty of Chemistry and Chemical Technology, University of Ljubljana, Večna pot 113, SI-1000 Ljubljana, Slovenia.
[2] Department of Molecular and Analytical Chemistry, University of Szeged, Dóm tér 7-8., H-6720 Szeged, Hungary.
[3] Department of Medical Microbiology, Albert Szent-Györgyi Health Center and Albert Szent-Györgyi Medical School, University of Szeged, Semmelweis u. 6, H-6725 Szeged, Hungary

*Corresponding authors. *Jakob Kljun*, Faculty of Chemistry and Chemical Technology, University of Ljubljana, Večna pot 113, SI-1000 Ljubljana, Slovenia. E-mail: jakob.kljun@fkkt.uni-lj.si; *Éva A. Enyedy*, Department of Molecular and Analytical Chemistry, University of Szeged, Dóm tér 7-8., H-6720 Szeged, Hungary. E-mail: enyedy@chem.u-szeged.hu

Contents

^{1}H NMR spectra of **B1**−**B4** SI-2
^{1}H NMR spectra of **ReB1**−**ReB4, ReB1Aq** SI-6
Crystallographic data for complexes **ReB1**, **ReB2** and **ReB4** SI-11
Maximum non-toxic concentrations of the chlorido complexes in Vero cells SI-15
DNA polymerase inhibition of the chlorido complexes: Ct and dCt values SI-15
UV-vis spectra of the chlorido complexes in DMSO SI-16
UV-vis spectra of **ReB3** and **ReB4** in 10% (*v*/*v*) DMSO/H_2O SI-17
^{1}H NMR spectra of **ReB1** and **ReB1Aq** in 10% (*v*/*v*) DMSO-d_6/H_2O SI-17
UV-vis spectra of **ReB1**−**ReB4** in 5% (*v*/*v*) DMSO/HEPES SI-18
^{1}H NMR spectra of **ReB2** and **ReB3** and their ligands in 10% (*v*/*v*) DMSO-d_6/HEPES SI-18
UV-vis spectra of **ReB1**−**ReB4** in 5% (*v*/*v*) DMSO/EMEM medium SI-19
UV-vis spectra of **ReB1**−**ReB4** in 5% (*v*/*v*) DMSO/blood serum SI-20
UV-vis spectra of **ReB1Aq** in HEPES buffer and in EMEM SI-20
UV-vis spectra of **ReB1Aq** in the presence of chloride ions at pH 6 .... SI-21
UV-vis spectra of ultrafiltrated **ReB1Aq** in the absence and presence of HSA SI-21
UV-vis spectra of **ReB1Aq** in the presence of HSA, MIM and NAC SI-22
Fluorescence emission spectra of HSA with **ReB1Aq** SI-24
References SI-24

Spectroscopic data for compound B1

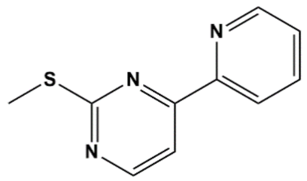

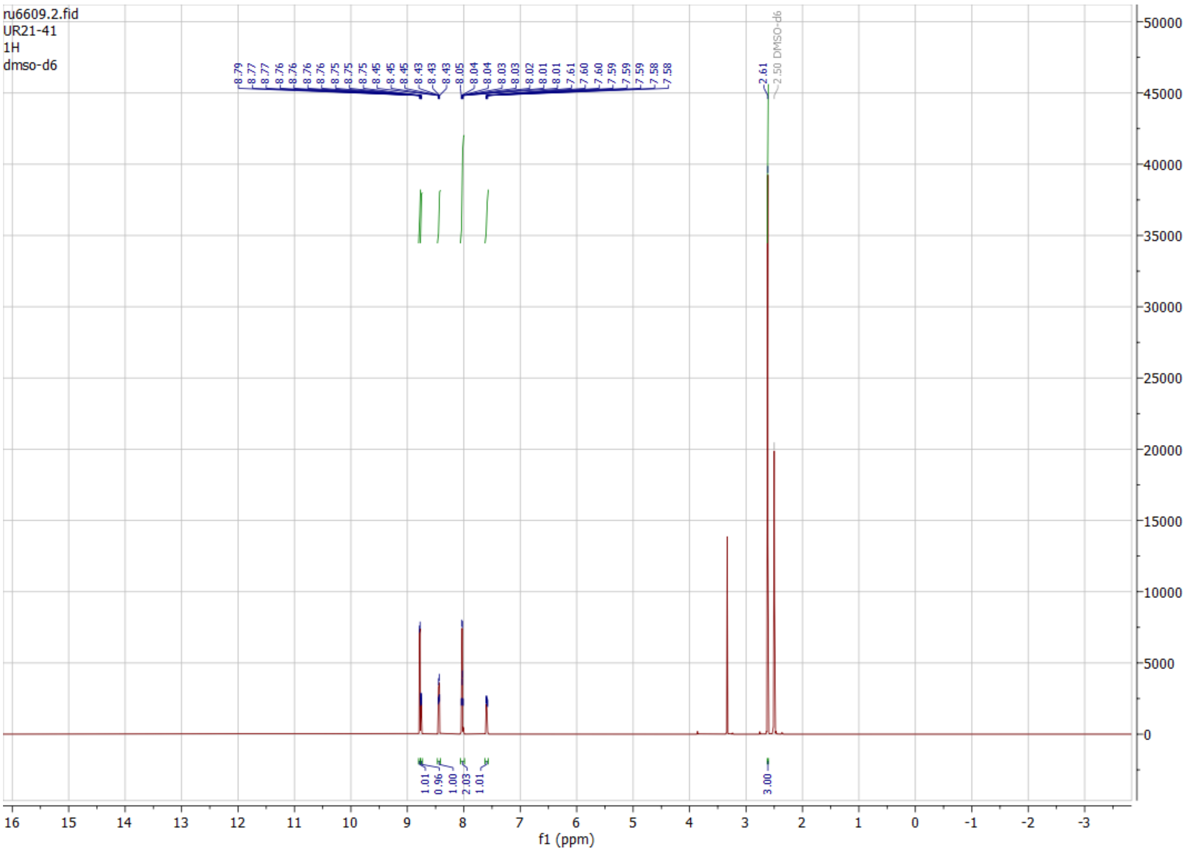

Figure S1. ^{1}H NMR spectrum of **B1** in DMSO-d_6.

Spectroscopic data for compound B2

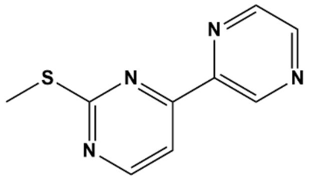

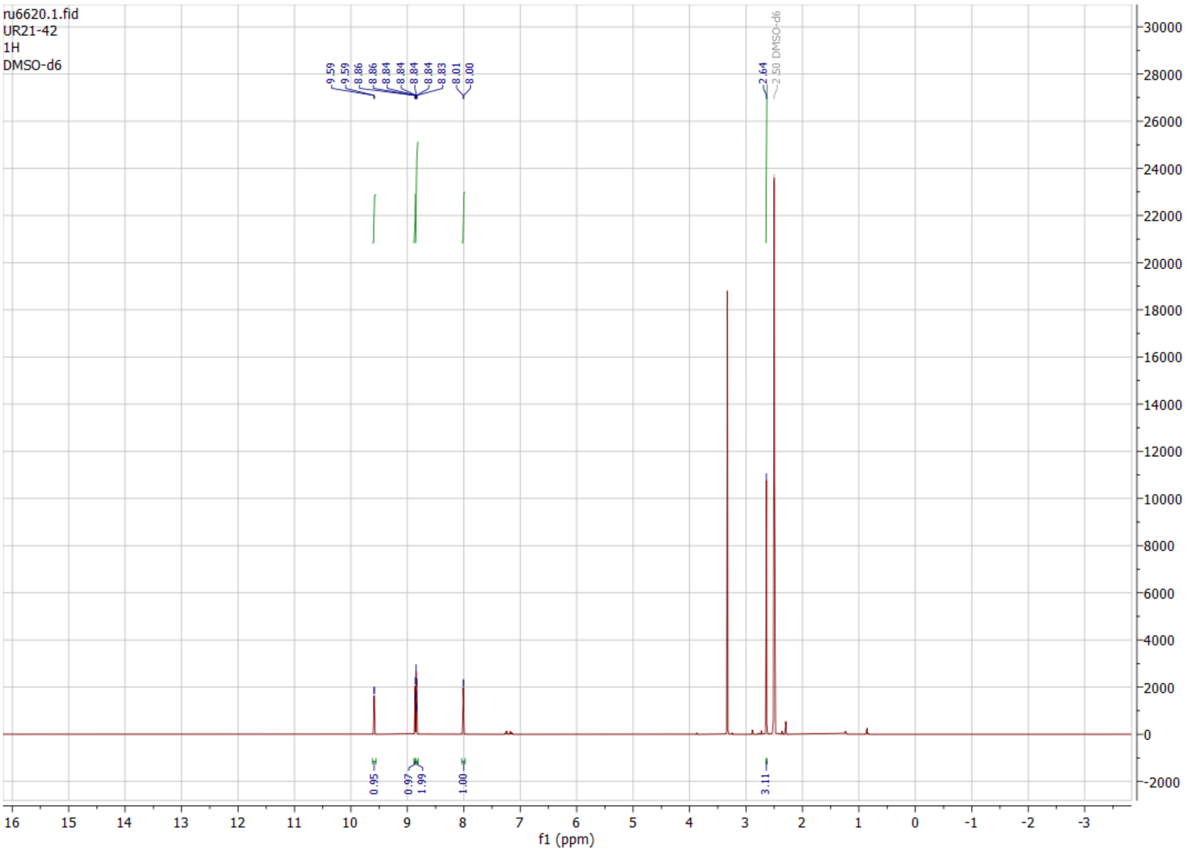

Figure S2. ^{1}H NMR spectrum of **B2** in DMSO-d_6.

Spectroscopic data for compound B3

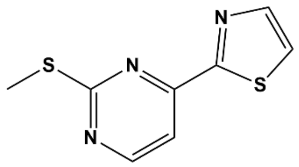

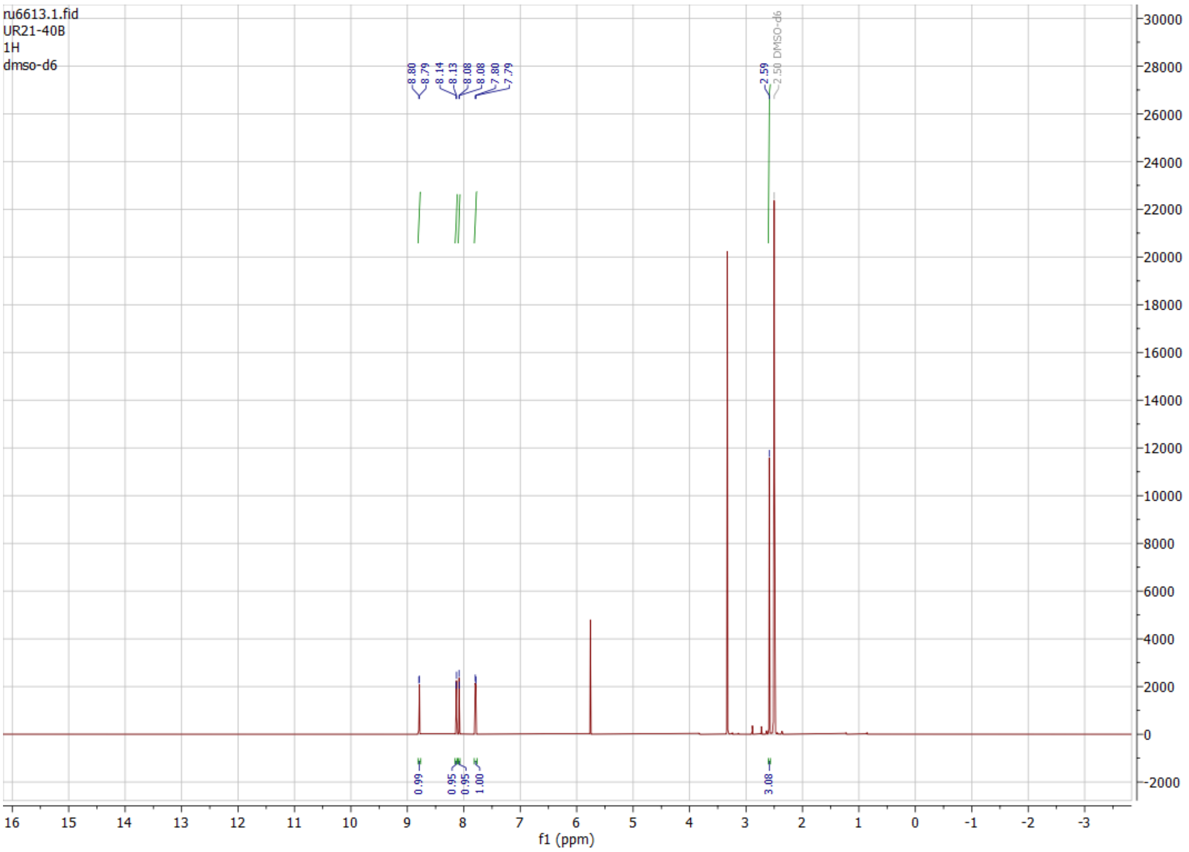

Figure S3. ^{1}H NMR spectrum of **B3** in DMSO-d_6.

Spectroscopic data for compound B4

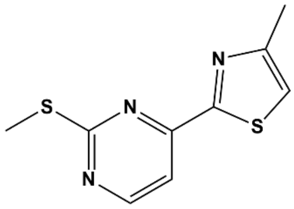

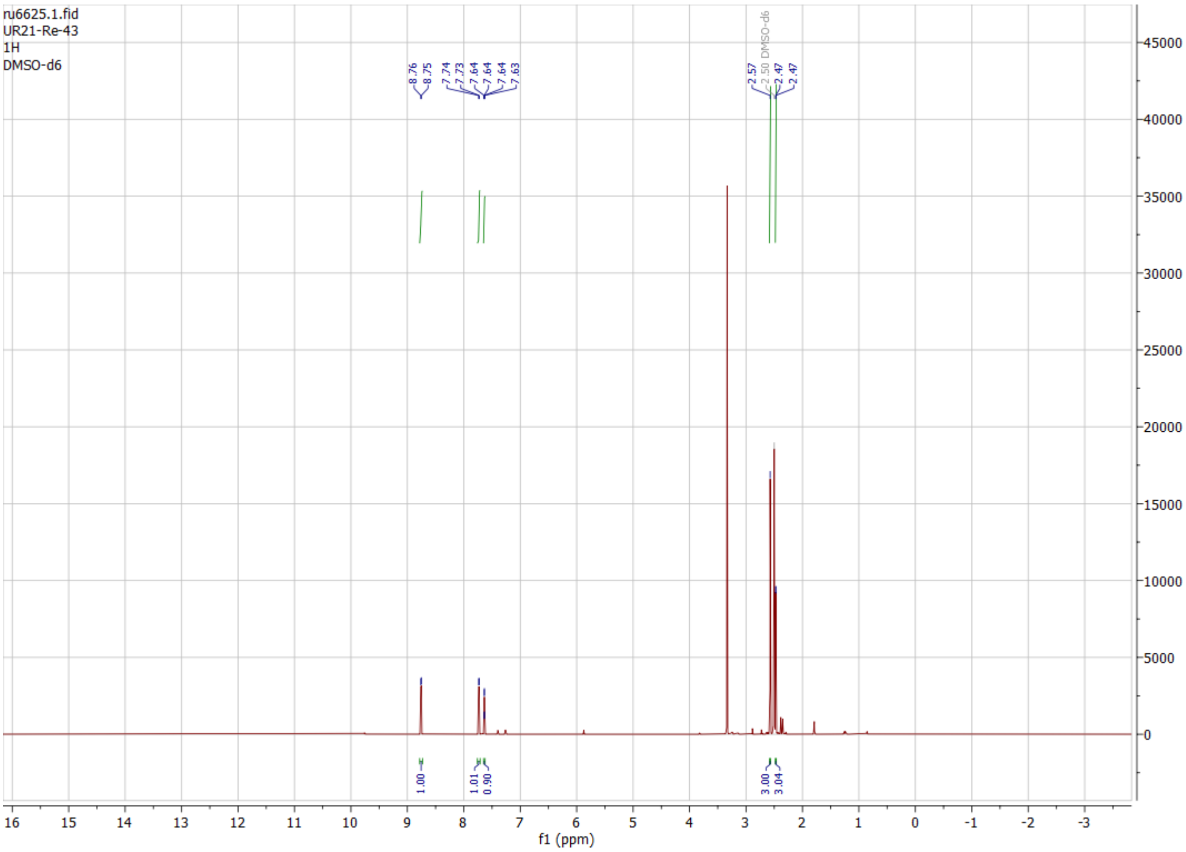

Figure S4. ^{1}H NMR spectrum of **B4** in DMSO-d_6.

Spectroscopic data for compound ReB1

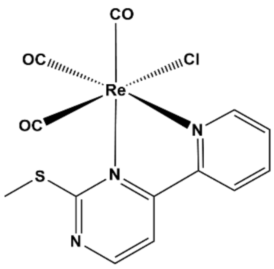

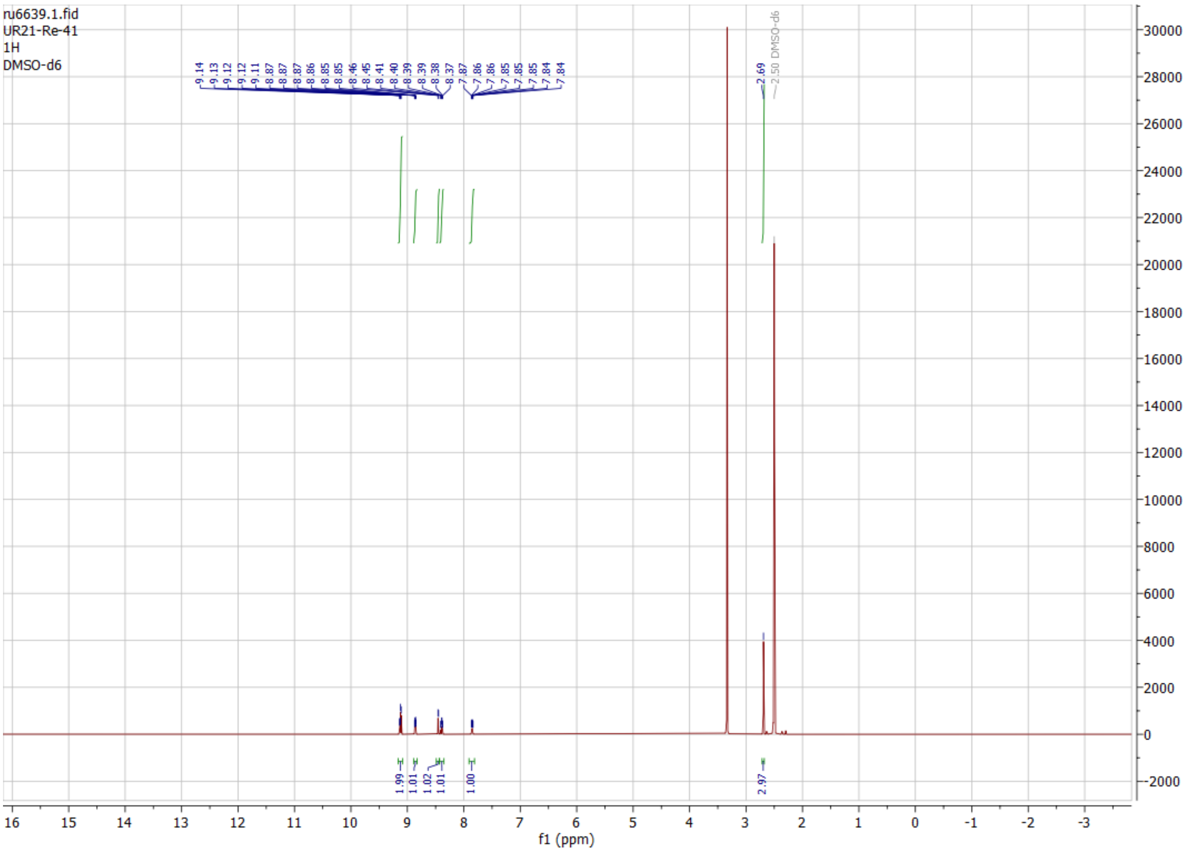

Figure S5. ^{1}H NMR spectrum of **ReB1** in DMSO-d_6.

Spectroscopic data for compound ReB2

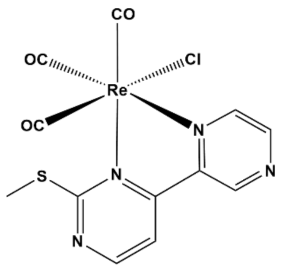

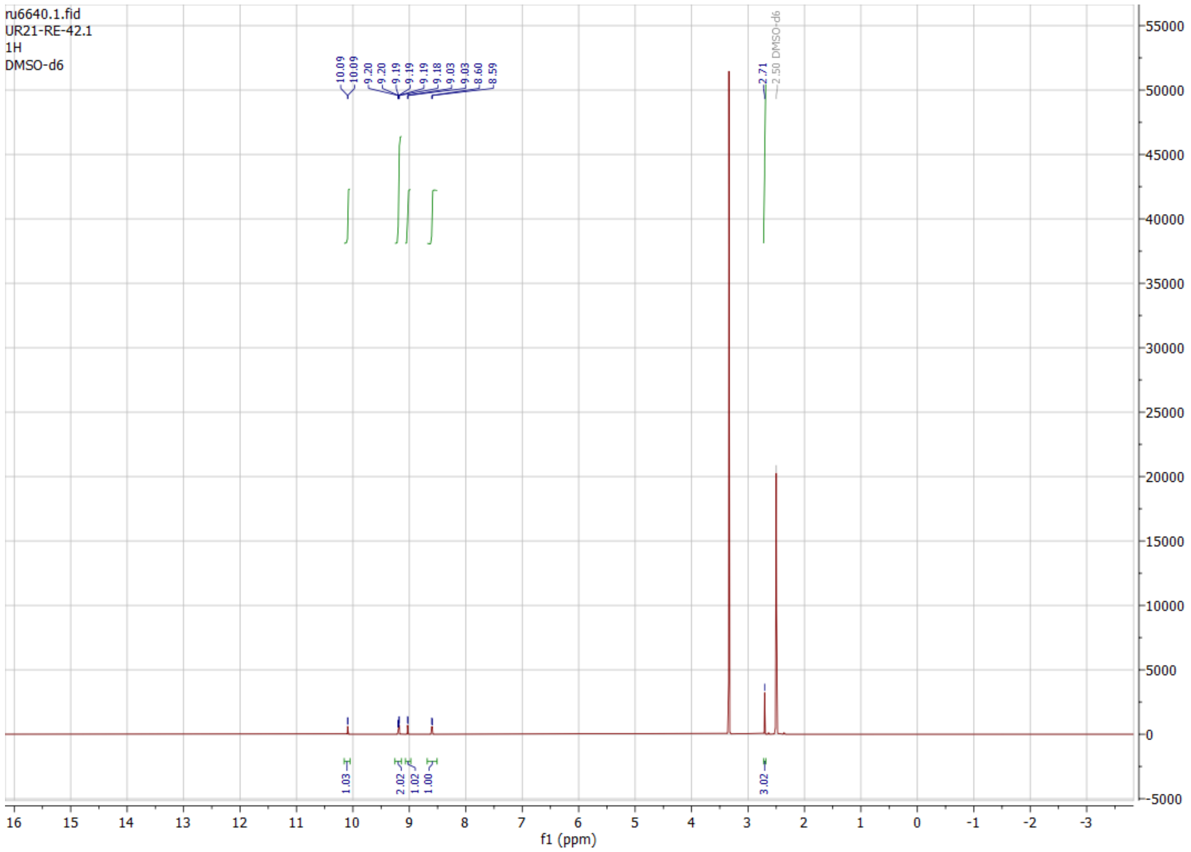

Figure S6. ^{1}H NMR spectrum of **ReB2** in DMSO-d_6.

Spectroscopic data for compound ReB3

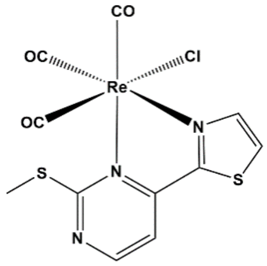

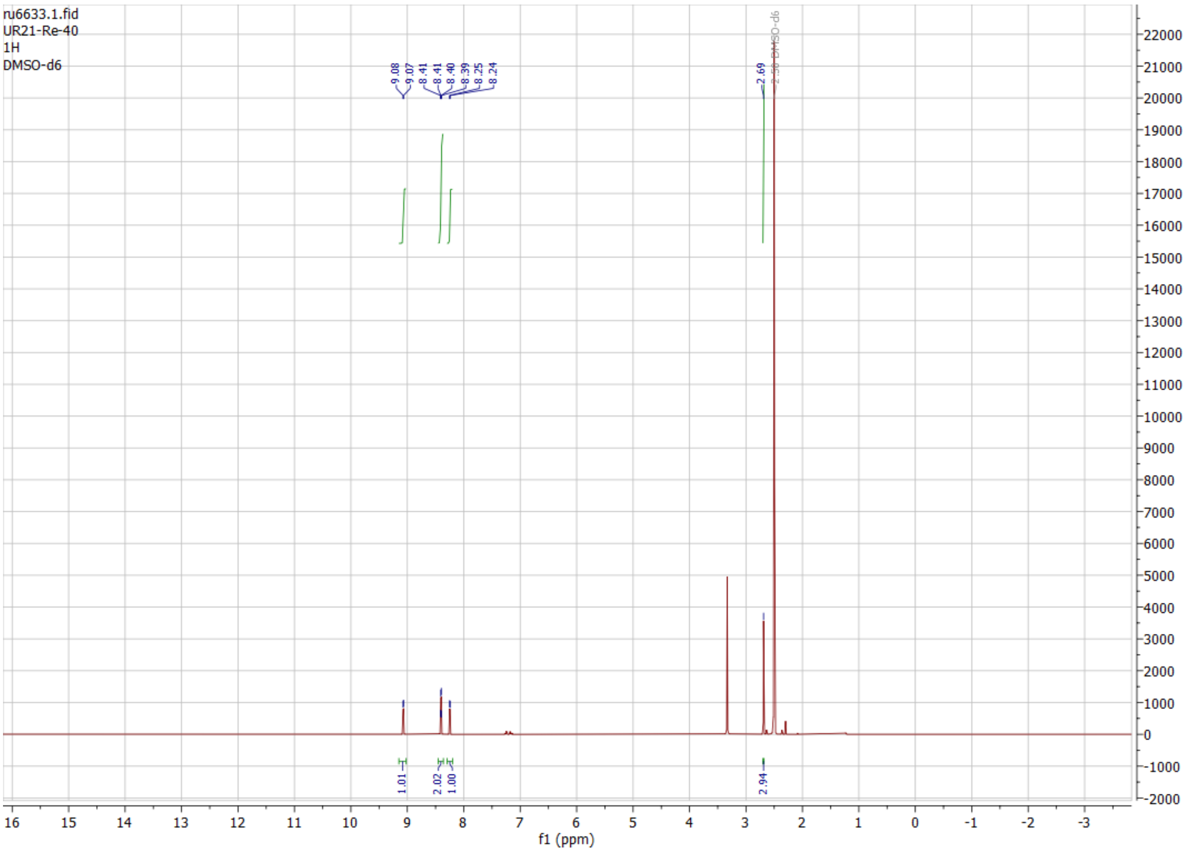

Figure S7. ^{1}H NMR spectrum of **ReB3** in DMSO-d_6.

Spectroscopic data for compound ReB4

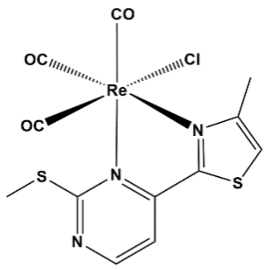

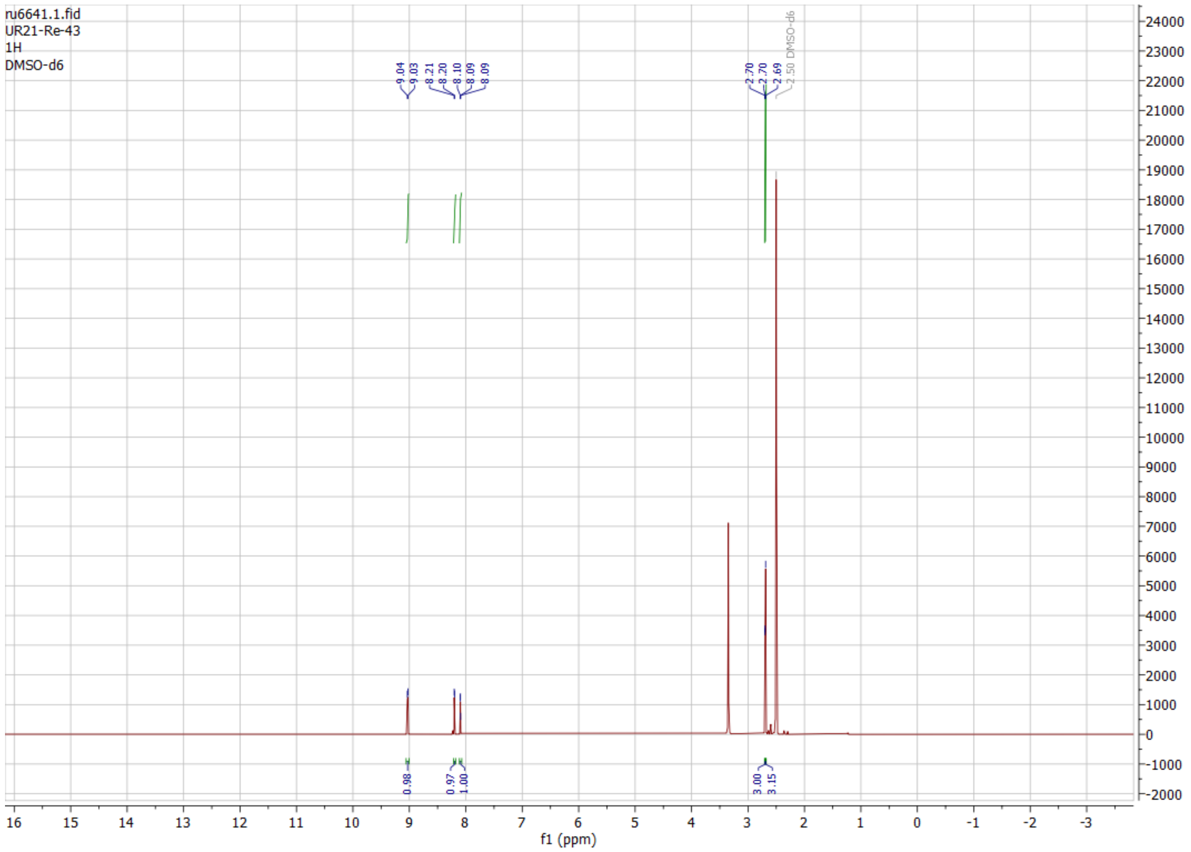

Figure S8. ^{1}H NMR spectrum of **ReB4** in DMSO-d_6.

Spectroscopic data for compound ReB1Aq

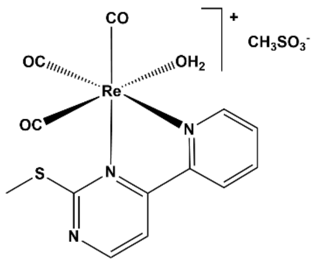

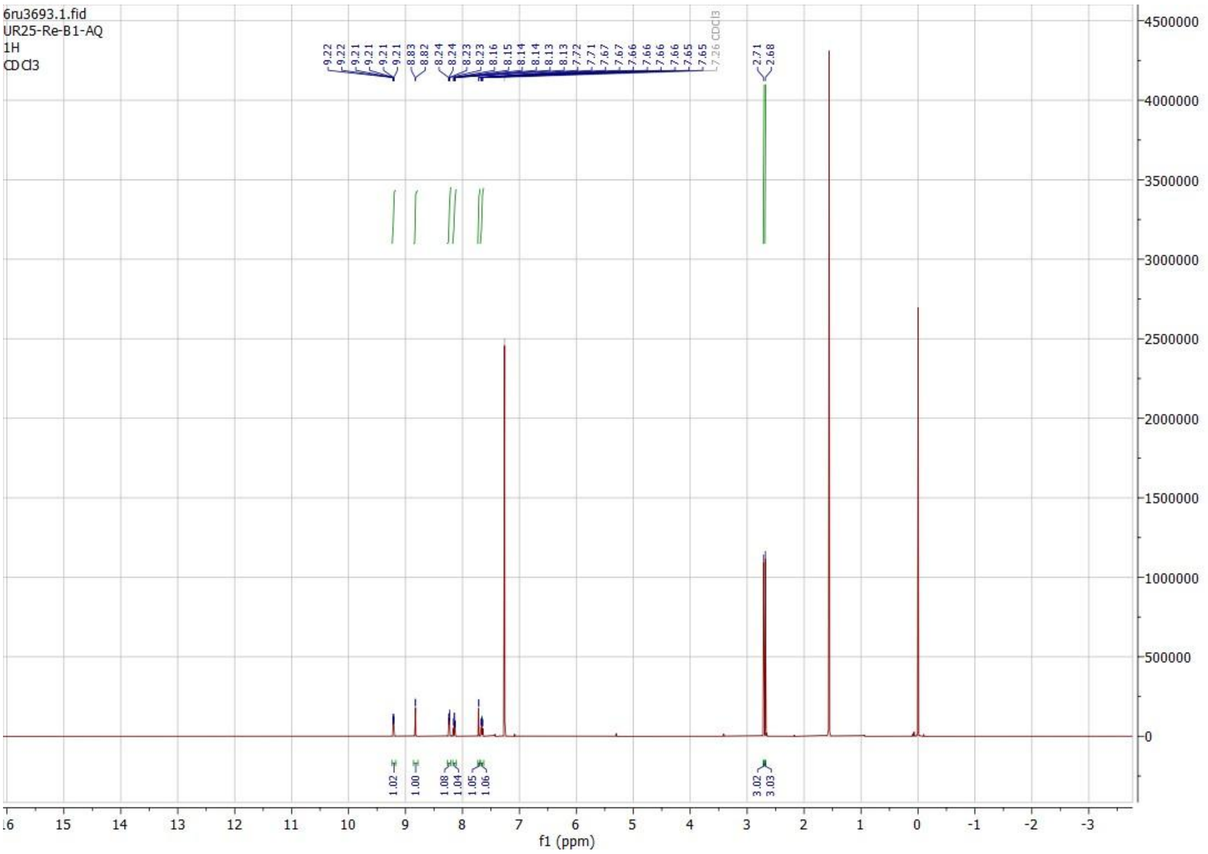

Figure S9. ^{1}H NMR spectrum of **ReB1Aq** in $CDCl_3$.

Additional crystallographic data

Table S1. Crystallographic data for complexes **ReB1**, **ReB2** and **ReB4**.

Compound	**ReB1**	**ReB2**	**ReB4**
CCDC deposition number	2509118	2509119	2509120
Identification code	moc418	moc216	moc219
Empirical formula	$C_{13}H_9ClN_3O_3ReS$	$C_{12}H_8ClN_4O_3ReS$	$C_{12}H_9ClN_3O_3ReS_2$
Formula weight	508.94	509.93	528.99
Temperature/K	150.00(10)	150.00(10)	150.00(10)
Crystal system	orthorhombic	triclinic	triclinic
Space group	$Pna2_1$	P-1	P-1
a/Å	17.6652(7)	9.9928(2)	7.8696(2)
b/Å	7.9485(3)	10.8281(2)	8.0531(3)
c/Å	10.7936(6)	13.9688(3)	12.9480(4)
α/°	90	77.308(2)	88.177(3)
β/°	90	84.533(2)	73.157(3)
γ/°	90	89.385(2)	87.614(3)
Volume/$Å^3$	1515.55(12)	1467.75(5)	784.53(4)
Z	4	4	2
$\rho_{calc}g/cm^3$	2.231	2.308	2.239
μ/mm^{-1}	8.346	8.620	8.194
F(000)	960.0	960.0	500.0
Crystal size/mm^3	0.1 × 0.1 × 0.03	0.3 × 0.2 × 0.1	0.2 × 0.15 × 0.1
Radiation	Mo Kα (λ = 0.71073)	Mo Kα (λ = 0.71073)	Mo Kα (λ = 0.71073)
2Θ range for data collection/°	5.62 to 54.96	4.842 to 54.968	5.064 to 54.968
Index ranges	$-22 \le h \le 21$, $-10 \le k \le 9$, $-13 \le l \le 14$	$-12 \le h \le 12$, $-14 \le k \le 14$, $-18 \le l \le 18$	$-10 \le h \le 10$, $-10 \le k \le 10$, $-16 \le l \le 16$
Reflections collected	11685	36896	18900
Independent reflections	3337 [R_{int} = 0.0335, R_{sigma} = 0.0328]	6682 [R_{int} = 0.0507, R_{sigma} = 0.0354]	3566 [R_{int} = 0.0541, R_{sigma} = 0.0380]
Data/restraints/parameters	3337/1/158	6682/0/399	3566/0/201
Goodness-of-fit on F^2	1.073	1.082	1.056
Final R indexes [I>=2σ (I)]	R_1 = 0.0341, wR_2 = 0.0717	R_1 = 0.0241, wR_2 = 0.0505	R_1 = 0.0228, wR_2 = 0.0476
Final R indexes [all data]	R_1 = 0.0390, wR_2 = 0.0740	R_1 = 0.0323, wR_2 = 0.0549	R_1 = 0.0267, wR_2 = 0.0497
Largest diff. peak/hole / e $Å^{-3}$	2.19/-0.97	1.24/-1.26	0.96/-1.06

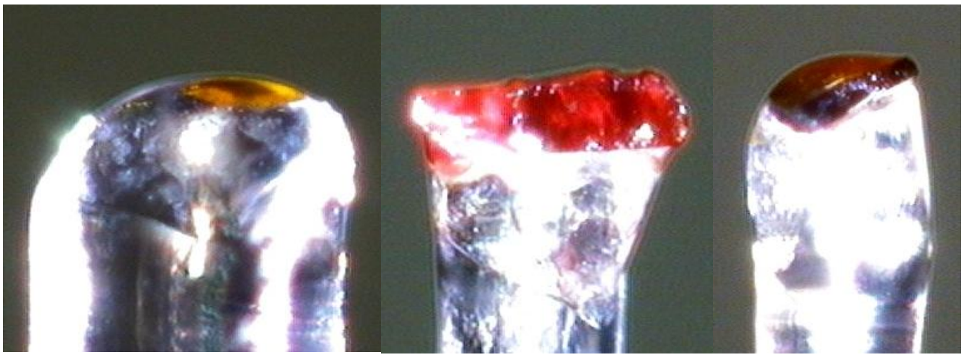

Figure S10. Photographs of the analyzed crystals of complexes **ReB1**, **ReB2** and **ReB4**.

Table S2. Crystallographic data for complexes **ReB1Aq**.

Compound	**(ReB1Aq)(CH_3SO_3)·2H_2O**
CCDC deposition number	2509121
Identification code	mod127
Empirical formula	$C_{14}H_{18}N_3O_9ReS_2$
Formula weight	622.63
Temperature/K	149.7(6)
Crystal system	monoclinic
Space group	$P2_1/c$
a/Å	7.3312(2)
b/Å	28.9602(6)
c/Å	9.4869(2)
α/°	90
β/°	96.803(2)
γ/°	90
Volume/$Å^3$	2000.01(8)
Z	4
ρ_{calc}g/cm^3	2.068
μ/mm^{-1}	6.337
F(000)	1208.0
Crystal size/mm^3	0.4 × 0.4 × 0.2
Radiation	Mo Kα (λ = 0.71073)
2Θ range for data collection/°	5.158 to 59.39
Index ranges	$-10 \leq h \leq 10$, $-40 \leq k \leq 40$, $-12 \leq l \leq 13$
Reflections collected	90368
Independent reflections	5483 [R_{int} = 0.0983, R_{sigma} = 0.0339]
Data/restraints/parameters	5483/0/271
Goodness-of-fit on F^2	1.119
Final R indexes [I>=2σ (I)]	R_1 = 0.0323, wR_2 = 0.0756
Final R indexes [all data]	R_1 = 0.0375, wR_2 = 0.0787
Largest diff. peak/hole / e Å^{-3}	2.04/-2.38

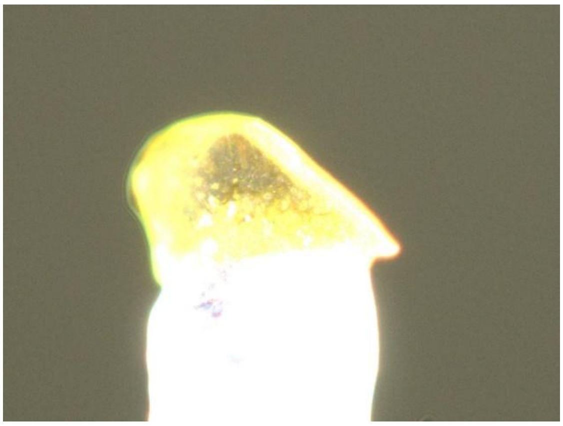

Figure S11. Photographs of the analyzed crystal of complex **ReB1Aq**.

Table S3. Selected bond lengths (Å) in *fac*-[Re(CO)$_3$(N,N)(Cl)] complexes and a diagram of atom notation. Comparison with previously reported rhenium complexes with pyridine-3,4-dicarboxylate methyl ester ligands **L1-L4**.

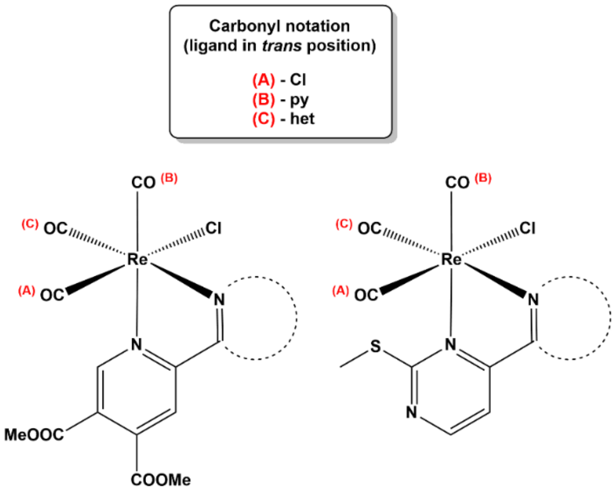

	**ReL2**[a,b]	**ReL3**[a]	**ReL4**[a]	**ReB1**	**ReB2**[b]	**ReB4**
Re1-C1A	1.916(6)<br>1.940(6)	1.906(7)	1.949(5)	1.909(9)	1.900(4)<br>1.899(4)	1.904(4)
C1A-O2A	1.153(6)<br>1.120(6)	1.143(8)	1.087(5)	1.151(11)	1.156(5)<br>1.160(5)	1.147(4)
Re1-C1B	1.915(5)<br>1.917(5)	1.906(6)	1.920(5)	1.936(12)	1.906(4)<br>1.906(4)	1.910(4)
C1B-O2B	1.151(6)<br>1.147(6)	1.167(7)	1.150(5)	1.175(14)	1.150(4)<br>1.151(5)	1.151(5)
Re1-C1C	1.921(5)<br>1.918(5)	1.920(6)	1.905(5)	1.881(13)	1.940(4)<br>1.926(4)	1.921(4)
C1C-O2C	1.142(6)<br>1.146(6)	1.149(7)	1.155(5)	1.139(15)	1.134(5)<br>1.150(5)	1.150(5)
Re1-N1$_{(pyridine/pyrimidine)}$	2.182(4)<br>2.180(4)	2.185(4)	2.194(3)	2.199(15)	2.216(3)<br>2.209(3)	2.226(3)
Re1-N$_{het}$	2.156(4)<br>2.166(4)	2.154(5)	2.186(4)	2.186(15)	2.151(3)<br>2.163(3)	2.169(3)
Re-Cl	2.4758(13)<br>2.4637(14)	2.4773(15)	2.4621(13)	2.471(2)	2.4811(9)<br>2.4899(10)	2.4970(8)

[a] Structural data taken from reference [SI1]
[b] These structures contain two molecules per asymmetric unit.

Table S4. Selected bond lengths (Å) in *fac*-[Re(CO)$_3$(N,N)(OH$_2$)]$^+$X$^-$ complexes and a diagram of atom notation. Comparison with previously reported rhenium complexes with pyridine-3,4-dicarboxylate methyl ester ligands **L1-L4**.

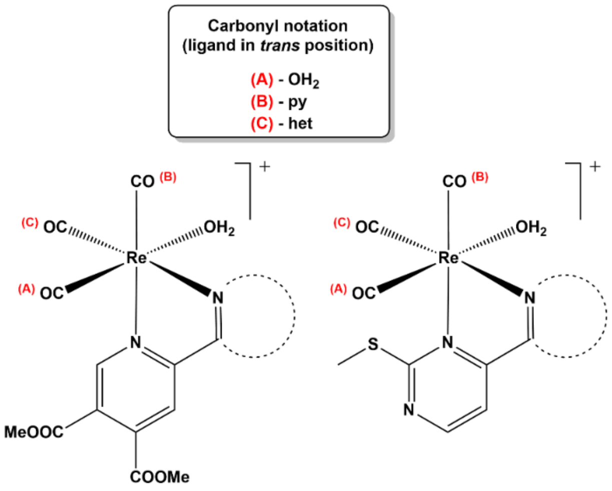

	**ReL1Aq**[a]	**ReL3Aq**[a]	**ReL4Aq**[a]	**ReB1Aq**
Re1-C1A	1.896(4)	1.903(4)	1.905(6)	1.902(4)
C1A-O2A	1.158(4)	1.151(5)	1.151(7)	1.153(5)
Re1-C1B	1.918(4)	1.920(4)	1.909(6)	1.916(4)
C1B-O2B	1.148(4)	1.143(5)	1.153(7)	1.151(5)
Re1-C1C	1.916(3)	1.916(4)	1.914(6)	1.916(4)
C1C-O2C	1.153(4)	1.151(5)	1.145(7)	1.152(5)
Re1-N1$_{(pyridine/pyrimidine)}$	2.172(2)	2.176(3)	2.186(4)	2.191(3)
Re1-N$_{het}$	2.164(3)	2.179(3)	2.166(4)	2.178(3)
Re-O$_{Aq}$	2.173(2)	2.170(3)	2.174(4)	2.177(3)

[a] Structural data taken from reference [SI1]
[b] These structures contain two molecules per asymmetric unit.

Table S5 Maximum non-toxic concentrations of the chlorido complexes determined in Vero cells (used as host for the evaluation of antiviral activity) by MTT assay. {Incubation time: 24 h}

	Maximum non-toxic concentration (μM) in Vero cells
**ReB1**	6.25
**ReB2**	100
**ReB3**	100
**ReB4**	25
***fac*-[Re(CO)$_3$(bpy)Cl])**	50

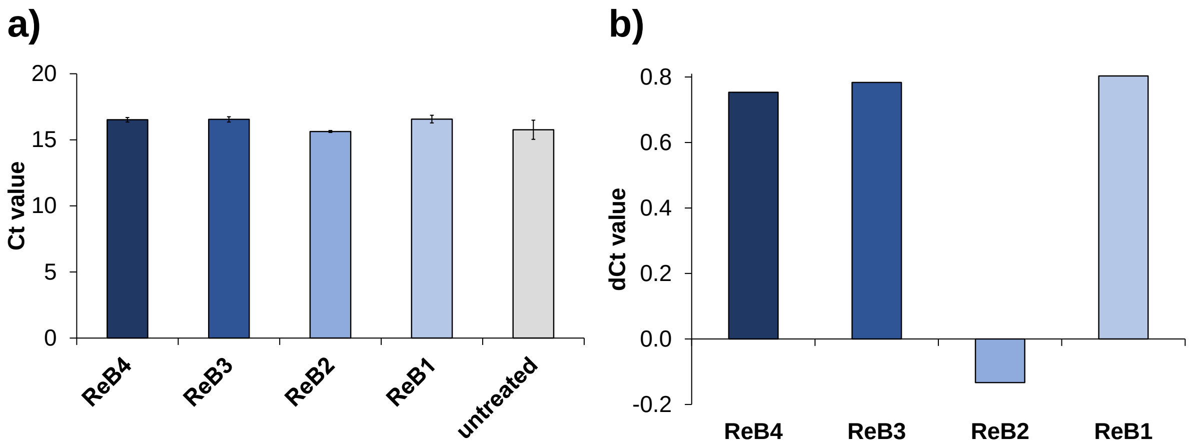

Figure S12. (a) Ct and (b) dCt values when the direct impact of the complexes on the DNA polymerase of qPCR was monitored. Lysates from Vero cells that were treated with the complexes, both infected and uninfected, were mixed in a 1:1 ratio, and the resulting Ct levels were similar to those obtained when lysates from HSV-2 infected cells and uninfected cells were mixed in the same ratio, thus the Ct levels remained consistent regardless of the treatment.

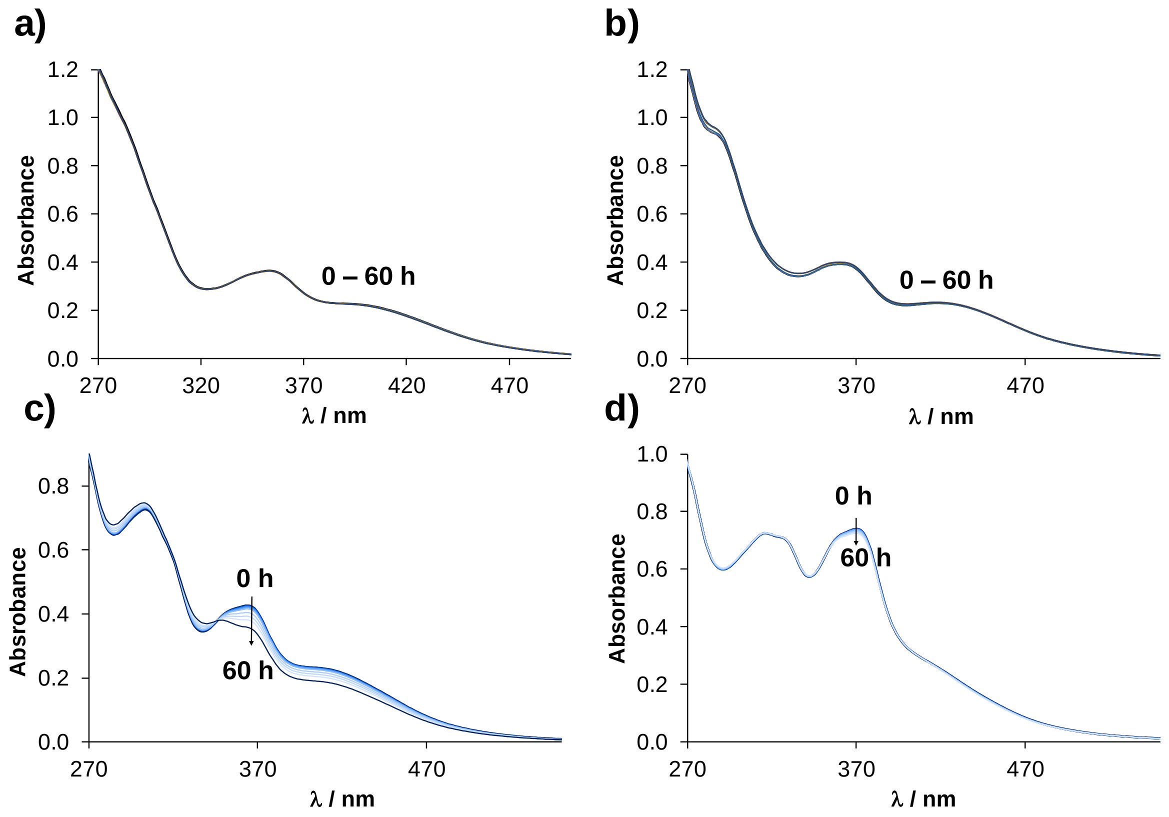

Figure S13. UV-vis spectra of a) **ReB1**, b) **ReB2**, c) **ReB3** and d) **ReB4** in DMSO followed over time. {$c_{complex}$ = 60 μM (**ReB1**), 65 μM (**ReB2**), 56 μM (**ReB3**) or 73 μM (**ReB4**); ℓ = 1 cm; T = 25.0 °C}

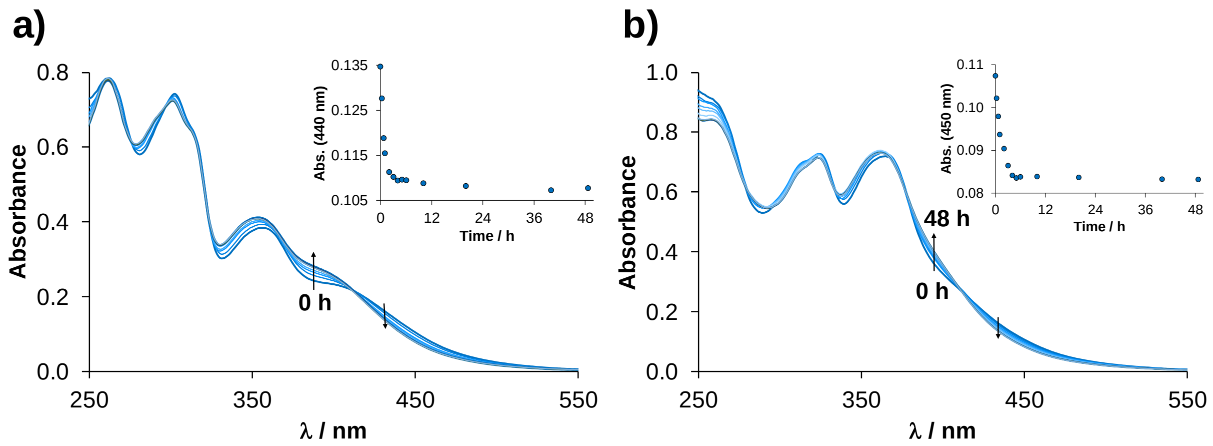

Figure S14. UV-vis spectra of a) **ReB3** and b) **ReB4** in 10% (*v*/*v*) DMSO/H_2O medium followed in time. Insets show absorbance values at 440 and 450 nm plotted against time for **ReB3** and **ReB4**, respectively. {$c_{complex}$ = 56 or 73 μM; pH ~ 6.5; ℓ = 1 cm; T = 25.0 °C}

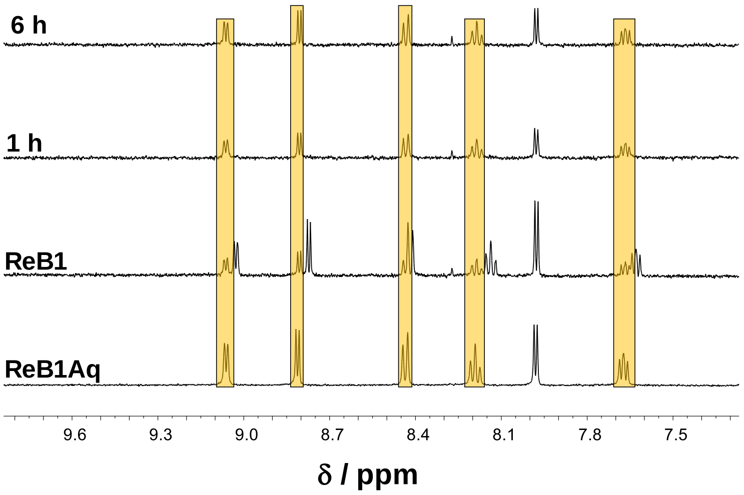

Figure S15. ^{1}H NMR spectra of **ReB1** and **ReB1Aq** in the low-field region in 10% (*v*/*v*) DMSO-d_6/H_2O medium followed in time. Rectangles indicate the appearance of aqua complex. {$c_{complex}$ = 0.5 mM; T = 25.0 °C}

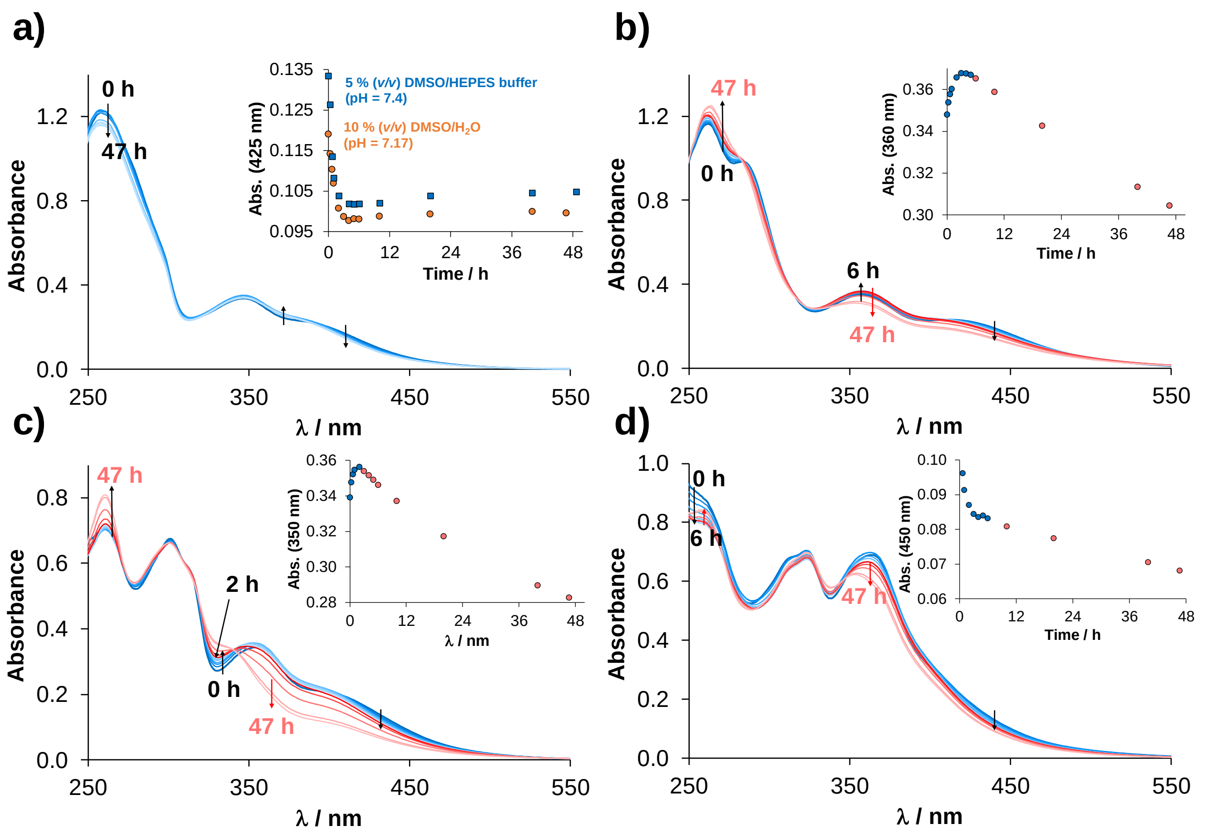

Figure S16. UV-vis spectra of a) **ReB1**, b) **ReB2**, c) **ReB3** and d) **ReB4** in 5% (*v/v*) DMSO/HEPES buffer (pH 7.4) medium followed in time. Insets show absorbance values plotted against time. {$c_{complex}$ = 60 µM (**ReB1**), 65 µM (**ReB2**), 56 µM (**ReB3**) or 73 µM (**ReB4**); pH = 7.4; ℓ = 1 cm; T = 25.0 °C}

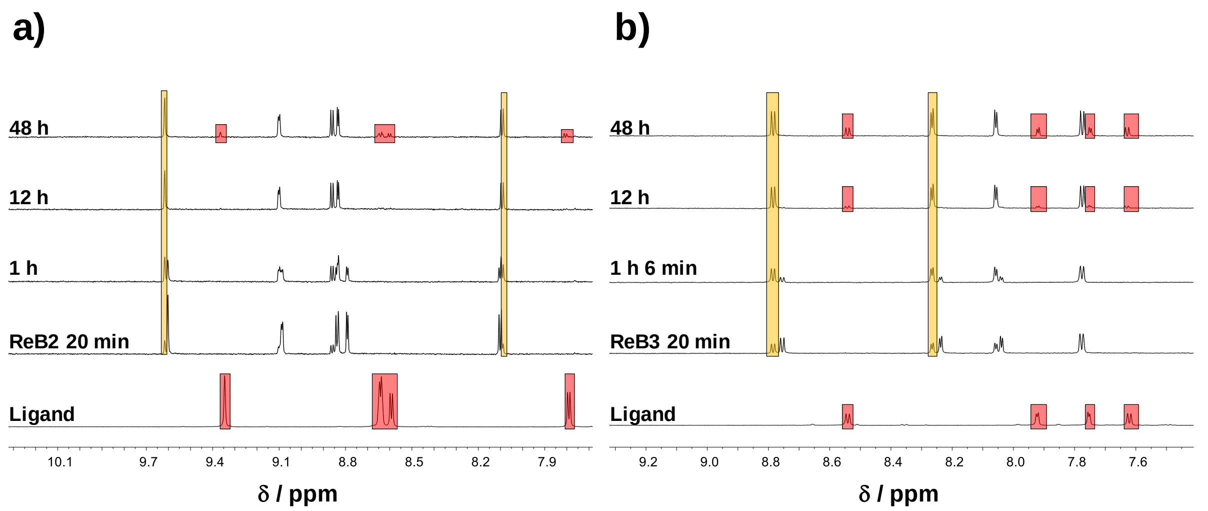

Figure S17. ^{1}H NMR spectra of a) **ReB2** and b) **ReB3** and their corresponding ligand in the low-field region in 10% (*v/v*) DMSO-d_6/HEPES followed in time. Yellow rectangles indicate the peaks belonging to the aqua complexes. Red rectangles indicate the appearance of free ligand {$c_{complex}$ = 0.5 mM; T = 25.0 °C}

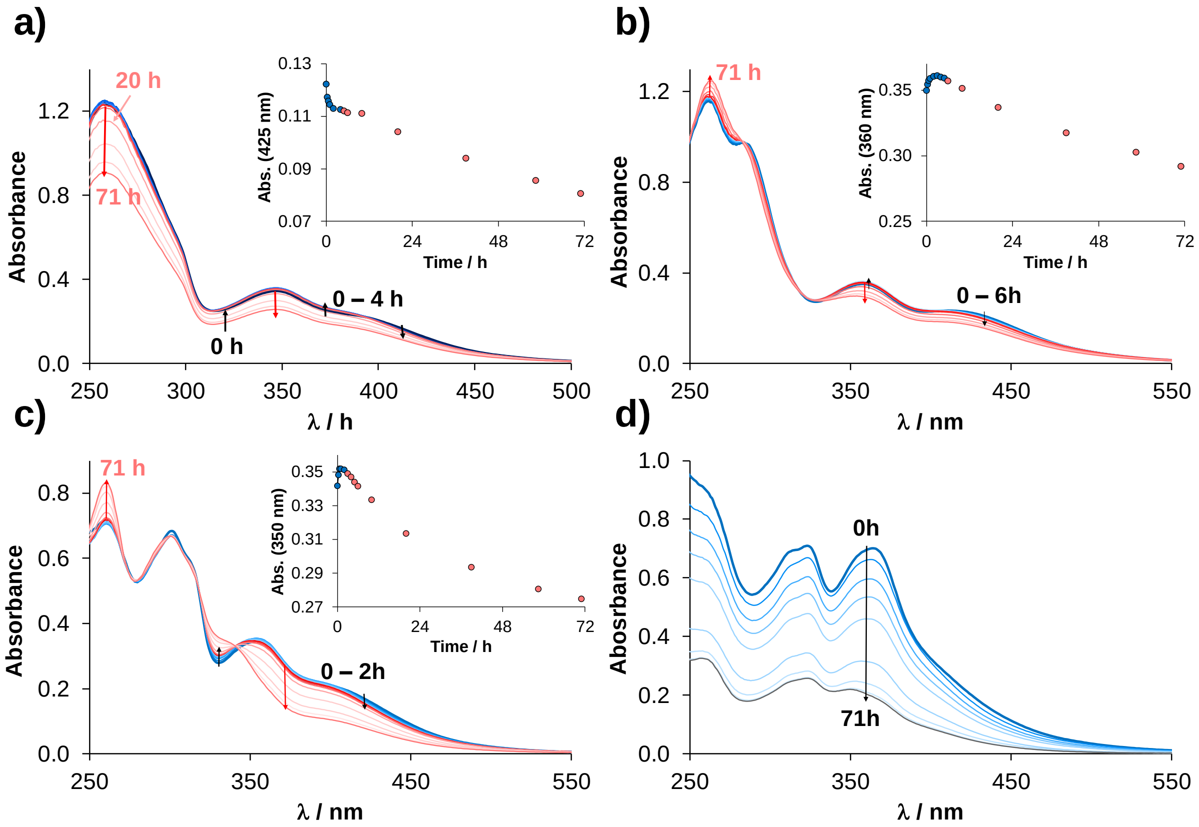

Figure S18. UV-vis spectra of a) **ReB1**, b) **ReB2**, c) **ReB3** and d) **ReB4** in 5% (*v/v*) DMSO/EMEM medium followed in time. Insets show absorbance values at plotted against time. {$c_{complex}$ = 60 μM (**ReB1**), 65 μM (**ReB2**), 56 μM (**ReB3**) or 73 μM (**ReB4**); pH = 7.4; ℓ = 1 cm; T = 25.0 °C}

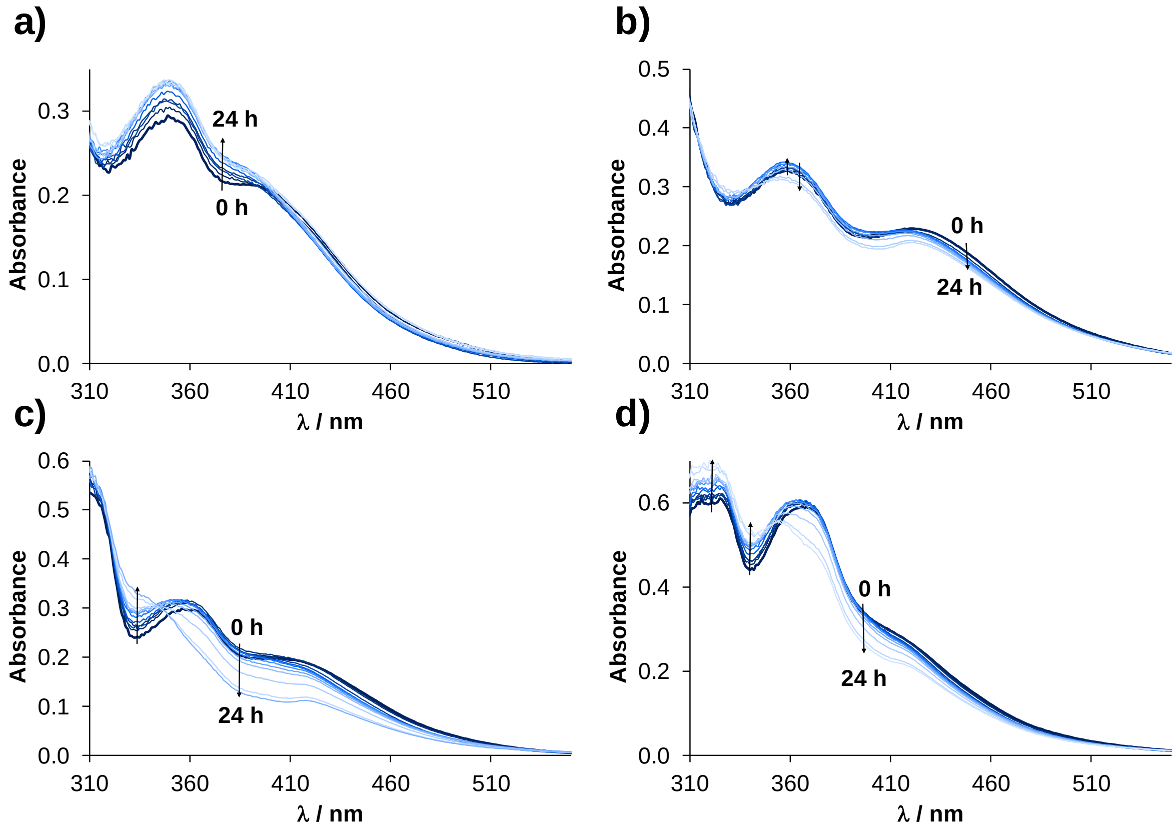

Figure S19. UV-vis spectra of a) **ReB1**, b) **ReB2**, c) **ReB3** and d) **ReB4** in 5% (*v/v*) DMSO/blood serum followed in time. The spectra are background (= diluted serum without the complexes) corrected. {$c_{complex}$ = 60 μM (**ReB1**), 65 μM (**ReB2**), 56 μM (**ReB3**) or 73 μM (**ReB4**); 4-fold diluted blood serum with HEPES buffer, pH = 7.4; ℓ = 1 cm; T = 25.0 °C}

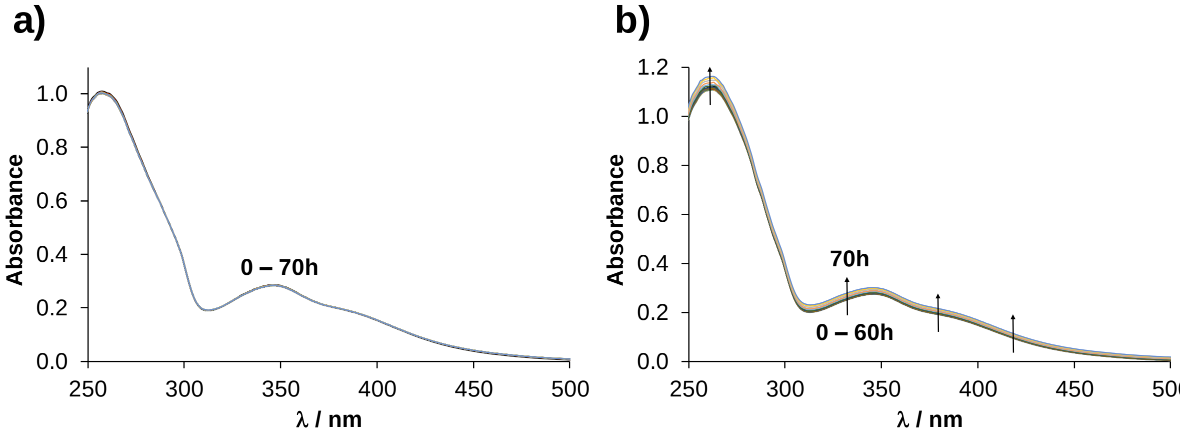

Figure S20. UV-vis spectra of **ReB1Aq** in a) HEPES buffer and in b) EMEM medium followed in time. {$c_{complex}$ = 50 μM; pH = 7.4; ℓ = 1 cm; T = 25.0 °C}

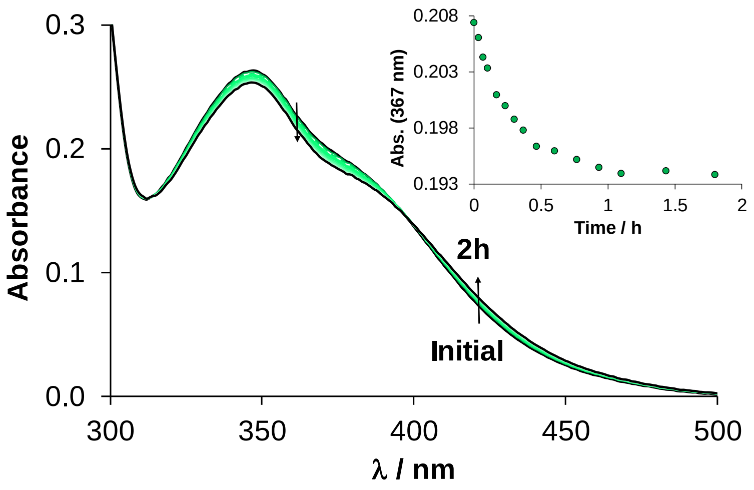

Figure S21. UV-vis spectra of **ReB1Aq** complex in the presence of chloride ions in HEPES buffer (pH = 6.0) followed over time. Inset shows the absorbance values at 367 nm (●) plotted against time. {c_{complex} = 50 μM; c_{Cl^-} = 250 mM; pH = 6.0; ℓ = 1 cm; T = 25.0 °C}

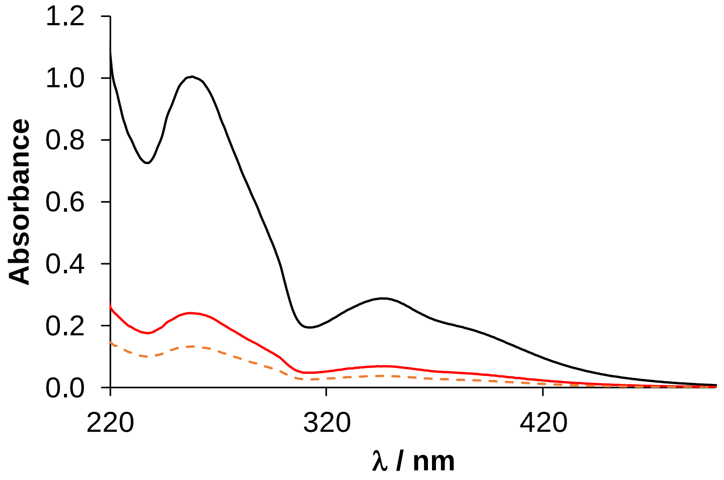

Figure S22. UV-vis spectra of ultrafiltrated **ReB1Aq** complex in the absence (red solid line) and presence of HSA (orange dashed line) along with the reference spectrum of the nonfiltered sample (black solid line). {c_{complex} = 50 μM; c_{HSA} = 25 μM; pH = 7.4 (10 mM HEPES buffer); ℓ = 1 cm; T = 25.0 °C}

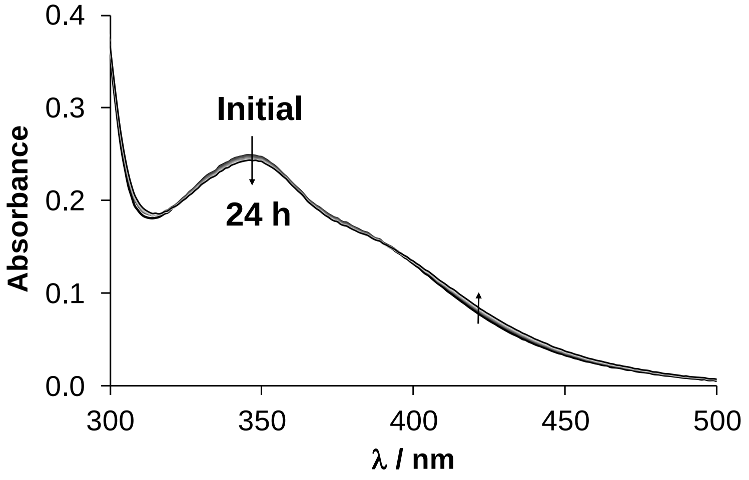

Figure S23. UV-vis spectra of **ReB1Aq** complex in the presence of 0.5 equivalent of HSA in HEPES buffer (pH = 7.4) followed over time. {c_{complex} = 50 μM; c_{HSA} = 25 μM; pH = 7.4; ℓ = 1 cm; T = 25.0 °C}

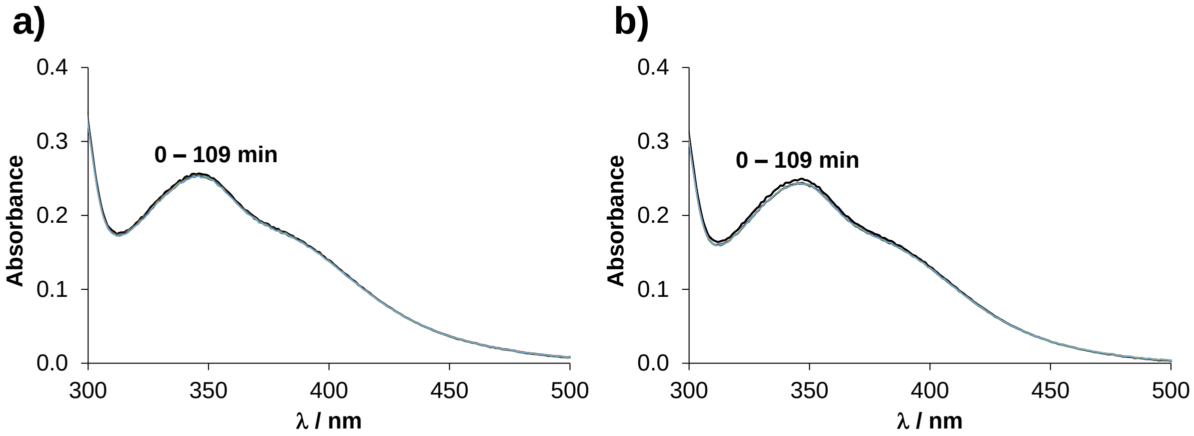

Figure S24. UV-vis spectra of **ReB1Aq** complex in the presence of a) 3 and b) 10 equivalents of MIM in HEPES buffer followed over time. {c_{complex} = 50 μM; c_{MIM} = 150 (a) or 500 μM (b); pH = 7.4; ℓ = 1 cm; T = 25.0 °C}

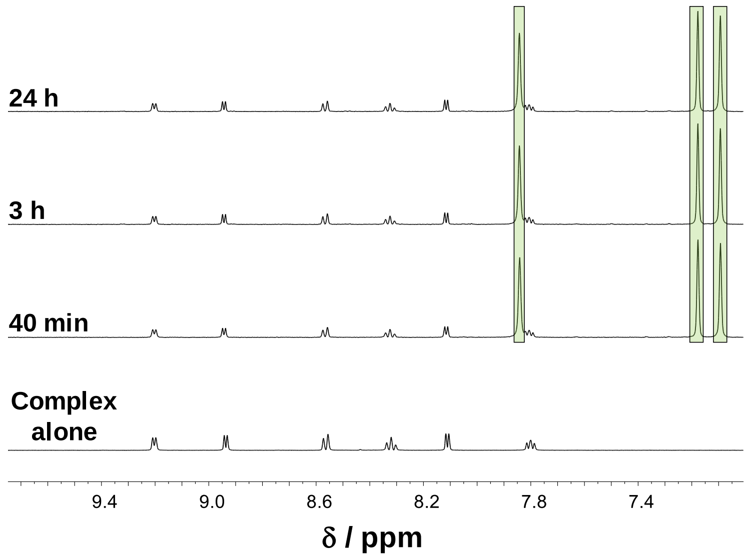

Figure S25. ^{1}H NMR spectra of **ReB1Aq** complex in the absence and presence of 10 equivalents of MIM in HEPES buffer followed over time. Green rectangles indicate the peaks of free MIM. {c_{complex} = 200 µM; c_{MIM} = 2 mM; 10% (*v/v*) D_2O/H_2O pH = 7.4; *T* = 25.0 °C}

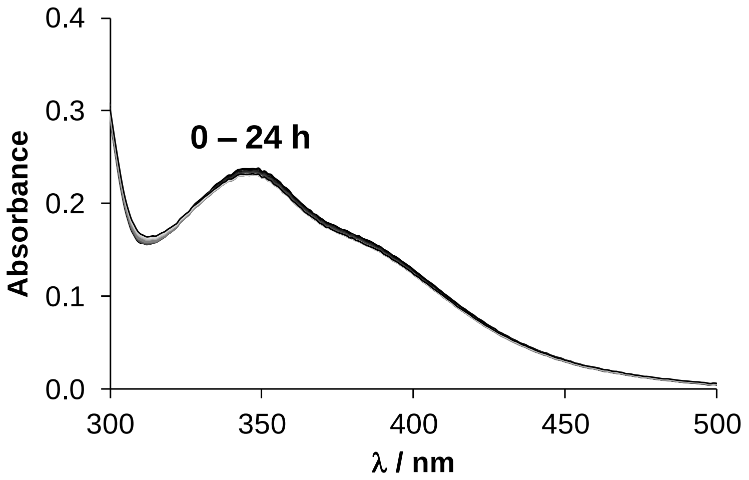

Figure S26. UV-vis spectra of **ReB1Aq** complex in the presence of 10 equivalents of NAC in HEPES buffer followed over time. {c_{complex} = 50 µM; $c_{\text{Ac-Cys}}$ = 500 µM; pH = 7.4; ℓ = 1 cm; *T* = 25.0 °C}

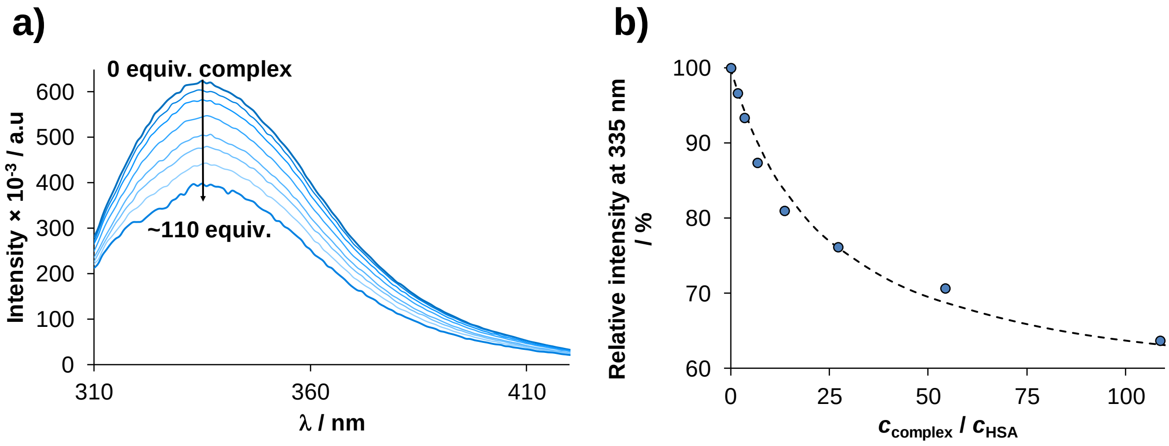

Figure S27. Fluorescence emission spectra of (a) HSA in the absence and presence of various amount of **ReB1Aq** complex. Experimental and calculated (dashed lines) relative emission intensities of (b) HSA at the corresponding wavelength in the presence of various amounts of **ReB1Aq** aqua complex. {c_{HSA} = 1 μM; $c_{complex}$ = 0 – 110 μM; λ_{EX} = 295 nm; pH = 7.4 (HEPES buffer); T = 25 °C; ℓ = 1×1 cm; incubation time: 24 h}

References

[SI1] Pivarcsik T, Kljun J, Rodriguez, S.C, et al. Structural and Solution Speciation Studies on *fac*-Tricarbonylrhenium(I) Complexes of 2,2′-Bipyridine Analogues. *ACS Omega* 2024; **9**: 44601–44615. https://doi.org/10.1021/acsomega.4c07117
